# Structural Fine-Tuning of Desmuramylpeptide NOD2 Agonists Defines Their *In Vivo* Adjuvant Activity

**DOI:** 10.1021/acs.jmedchem.1c00644

**Published:** 2021-05-27

**Authors:** Samo Guzelj, Sanja Nabergoj, Martina Gobec, Stane Pajk, Veronika Klančič, Bram Slütter, Ruža Frkanec, Adela Štimac, Primož Šket, Janez Plavec, Irena Mlinarič-Raščan, Žiga Jakopin

**Affiliations:** † Faculty of Pharmacy, University of Ljubljana, SI-1000 Ljubljana, Slovenia; ‡ Div. BioTherapeutics, Leiden Academic Centre for Drug Research, Leiden University, 2333 CC Leiden, The Netherlands; § Centre for Research and Knowledge Transfer in Biotechnology, University of Zagreb, 10000 Zagreb, Croatia; ∥ Slovenian NMR Centre, National Institute of Chemistry, SI-1000 Ljubljana, Slovenia

## Abstract

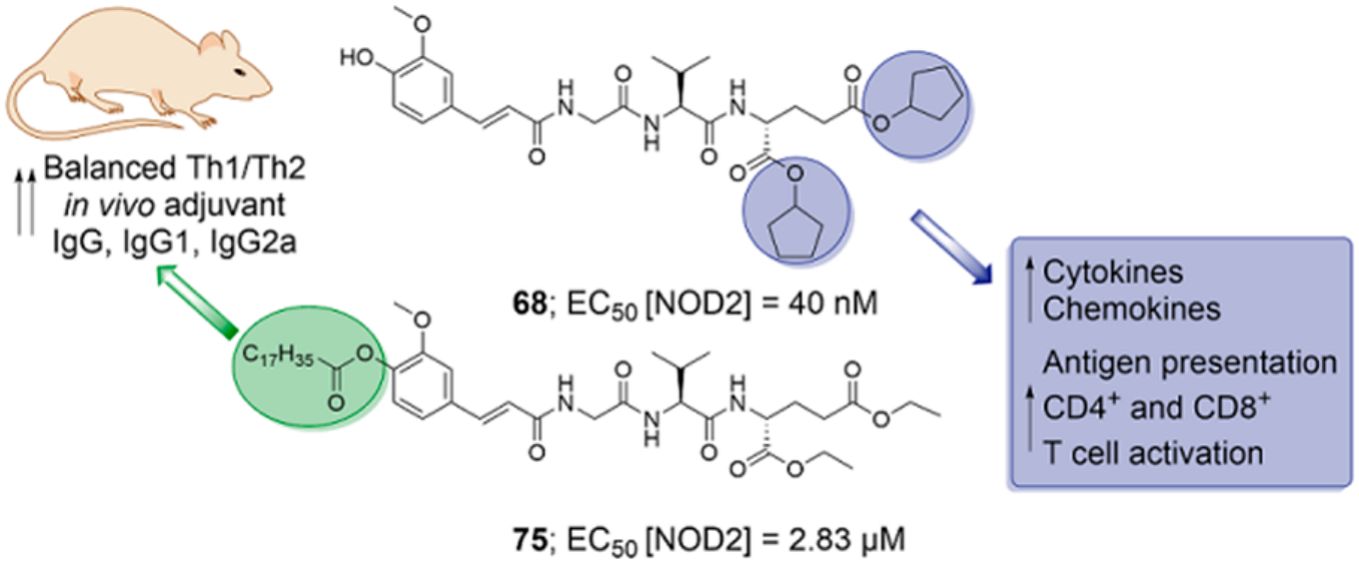

We report on the design, synthesis, and biological evaluation of a series of nucleotide-binding oligomerization-domain-containing protein 2 (NOD2) desmuramylpeptide agonists with improved *in vitro* and *in vivo* adjuvant properties. We identified two promising compounds: **68**, a potent nanomolar *in vitro* NOD2 agonist, and the more lipophilic **75**, which shows superior adjuvant activity *in vivo*. Both compounds had immunostimulatory effects on peripheral blood mononuclear cells at the protein and transcriptional levels, and augmented dendritic-cell-mediated activation of T cells, while **75** additionally enhanced the cytotoxic activity of peripheral blood mononuclear cells against malignant cells. The C_18_ lipophilic tail of **75** is identified as a pivotal structural element that confers *in vivo* adjuvant activity in conjunction with a liposomal delivery system. Accordingly, liposome-encapsulated **75** showed promising adjuvant activity in mice, surpassing that of muramyl dipeptide, while achieving a more balanced Th1/Th2 immune response, thus highlighting its potential as a vaccine adjuvant.

## 1 Introduction

Defense against invading pathogens in vertebrates is accomplished through coordinated responses of the nonspecific innate and the antigen-specific adaptive immune systems. The former orchestrates the first line of defense through the action of a superfamily of pattern recognition receptors (PRRs). PRRs are responsible for recognition of “nonself” features, which are conserved microbial components that are also known as pathogen-associated molecular patterns.^1,2^ As well as promotion of the immediate innate immune response, PRRs are involved in shaping of the gradually forming adaptive immune responses through engagement of antigen-presenting cells.^3^ Thus, both naturally and synthetically derived modulators of PRRs have been of considerable interest for medicinal chemists for development as vaccine adjuvants.^4−7^

Nucleotide-binding oligomerization-domain-containing protein 2 (NOD2) belongs to the intracellular NOD-like receptor family of PRRs, and it is composed of three motifs: (i) two effector N-terminal caspase recruitment domains (CARDs); (ii) a centrally located nucleotide-binding domain that is required for oligomerization; and (iii) a C-terminal leucine-rich repeat domain that is implicated in ligand recognition.^8^ NOD2 is primarily expressed in leukocytes and intestinal epithelial cells (especially Paneth cells), where it is required to sense bacterial cell wall peptidoglycan fragments that enter the cytosol.^8,9^ The minimal essential peptidoglycan substructure that can still activate NOD2 is muramyl dipeptide (MDP), a glycopeptide in the cell wall of both Gram-positive and Gram-negative bacteria.^10−13^ MDP comprises *N*-acetylmuramic acid (MurNAc) and an l-alanine-d-isoglutamine dipeptide, which is attached to the MurNAc via a lactic acid spacer. Recognition of MDP is followed by self-oligomerization, through which NOD2 recruits the adaptor protein receptor-interacting serine/threonine kinase 2, RIP2, via CARD–CARD interactions. This triggers the downstream signaling cascades that include the mitogen-activated protein kinase and nuclear factor κB (NF-κB) pathways, which results in a wide array of immune responses.^14^ These are characterized by release of pro-inflammatory cytokines, chemokines, and antimicrobial factors (including defensins), generation of reactive nitrogen species, and recruitment and priming of neutrophils, inflammatory monocytes, and dendritic cells (DCs).^15−17^ Of note, NOD2 activation also facilitates autophagy, an essential process for efficient antigen processing and activation of the adaptive immune system.^18,19^

Due to their broad immunomodulatory effects, NOD2 agonists are of significant clinical relevance. They have been highlighted for providing nonspecific protective effects against bacterial and viral infections.^20^ Furthermore, NOD2 is an enticing target for expansion of the currently limited selection of vaccine adjuvants.^21^ As well as generating robust and sustainable systemic immune responses, recent reports have additionally extended the potential of NOD2-activating adjuvants to mucosal vaccines.^22,23^ These represent an attractive alternative to conventional vaccines, as they generate mucosal immune responses, which are essential for protection against pathogens transmitted through mucosal surfaces. Finally, the developing field of cancer immunotherapy has highlighted NOD2 agonists as potential immunotherapeutics, either as adjuvants in cancer vaccines, or by directly enhancing immune cell antitumor activity.^7^

**Figure 1:**
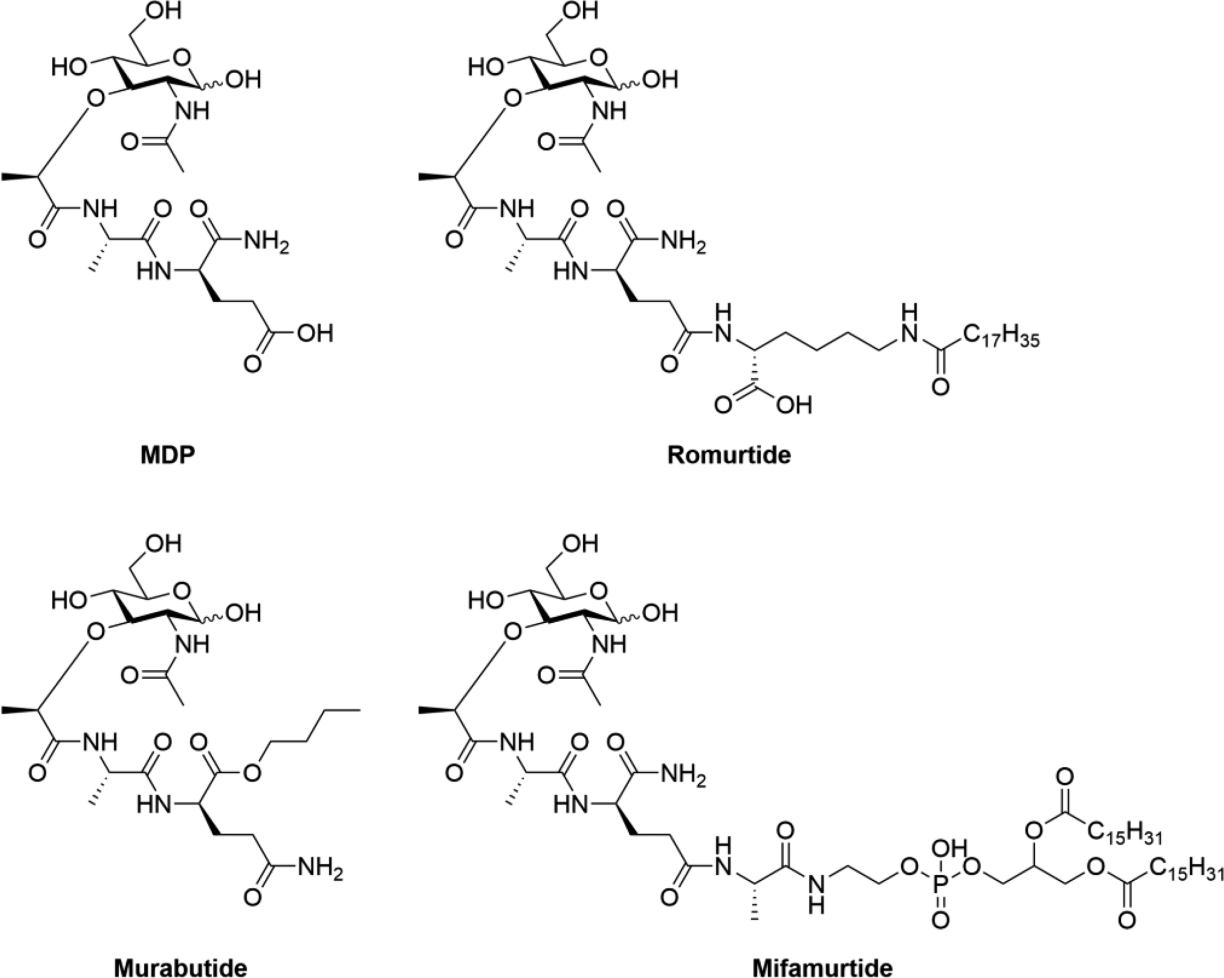
MDP and its representative derivatives.

MDP was first recognized as the minimal effective component of Freund’s complete adjuvant.^24^ While MDP promotes both innate and adaptive immune responses, its use in the clinic is hindered by its strong pyrogenicity,^25,26^ rapid elimination,^27^ and metabolic instability.^28^ To circumvent these issues and to improve its clinical utility, structural modifications of MDP have been studied extensively, with several reviews available that have comprehensively described their structure–activity relationships.^29−31^ Notably, two lipophilic derivatives of MDP, known as romurtide^32^ and mifamurtide,^33^ are currently in use for the treatment of leukopenia and osteosarcoma, respectively, while a hydrophilic MDP derivative, murabutide, has been investigated in several clinical trials as a vaccine adjuvant (Figure 1).^34,35^

The discovery that the full glycopeptide scaffold is not mandatory for NOD2 agonism led to the concept of desmuramylpeptides, a class of compounds that lack the MurNAc moiety. Replacement of this carbohydrate fragment with suitable surrogates can simplify their synthesis and allow for easier manipulation of their lipophilicity. Most desmuramylpeptides comprise the preserved or slightly varied MDP dipeptide motif plus various lipophilic groups, which have included carbocycles,^36^ adamantane,^37−39^ and diverse aromatic moieties.^40−46^ Here, we describe the design, synthesis, and biological evaluation of a series of novel acylated desmuramyltripeptides that are based on the structure of **1**, which is a potent NOD2 agonist that was previously reported by our group and contains a *trans*-feruloyl-glycine MurNAc mimetic.^46^ Our current study identified the structural requirements for *in vitro* and *in vivo* immunostimulatory activity of **1**, which led to the surprising observation that NOD2 activation *in vitro* does not necessarily coincide with its adjuvant activity *in vivo*. We identified **68** as a potent *in vitro* NOD2 agonist with more than 2-fold improved potency over **1** and **75** with superior adjuvant activity *in vivo*. Compounds **68** and **75** induced proinflammatory transcriptional changes and cytokine production in peripheral blood mononuclear cells (PBMCs), both alone and in combination with lipopolysaccharide (LPS), and enhanced antigen presentation of DCs. Furthermore, **75** stimulated the cytotoxic activity of PBMCs against malignant cells and, importantly, had promising *in vivo* adjuvant activity with a balanced Th1/Th2 immune response in a mouse model of adjuvanticity.

## 2 Results and Discussion

### 2.1 Design

Compound **1** comprises a *trans*-feruloyl-glycine MurNAc mimetic attached to the l-valine-d-glutamate dipeptide, and it showed good NOD2 agonistic activity with twice the potency of MDP in a HEK293 cell assay with overexpressed NOD2. It also enhanced the LPS-induced release of proinflammatory cytokines and was devoid of pyrogenicity, although it showed weak adjuvant activity in a mouse model of adjuvanticity.

**Figure 2:**
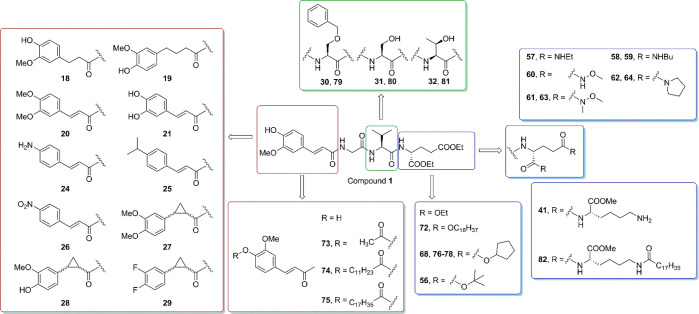
Design of novel desmuramylpeptides based on **1**.

Recent biophysical data have suggested that MDP interacts with NOD2 through the leucine-rich repeat domain.^47^ Maekawa et al. (2016) solved the crystal structure of NOD2 apoprotein in its ADP-bound inactive form.^48^ To date, however, no crystal structure of NOD2 with a bound ligand has been reported, and thus the exact binding mechanisms of MDP and its related compounds (including **1**) remain to be defined. Previous studies of **1** have suggested that the aromatic ring of the *trans*-feruloyl moiety contributes to NOD2 binding with π–π stacking and cation−π interactions, while both the 4-hydroxy and 3-methoxy groups form H-bonds with residues in the putative binding site.^46^ To determine whether these interactions can be optimized, we designed several derivatives with modified substitutions of the aromatic ring (Figure 2). Furthermore, we evaluated both increased flexibility of (i.e., reduction of the feruloyl alkene bond) and conformational restrictions to (i.e., introduction of the cyclopropyl ring) the *trans* geometry. Notably, cyclopropyl fragments have an established track record in drug design, in part due to their “locking” of *E*/*Z* isomerizable alkene bonds in favorable conformations.^49^ Exploration of the chemical space around the MurNAc surrogate moiety thus yielded compounds **18**–**21** and **24**–**28** (Figure 2, Table 1).

**Table 1:**
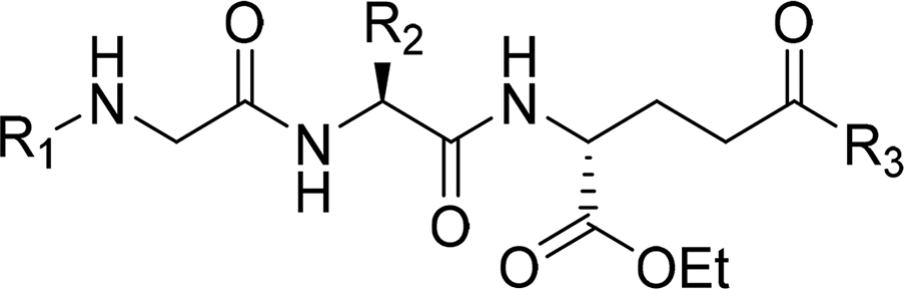

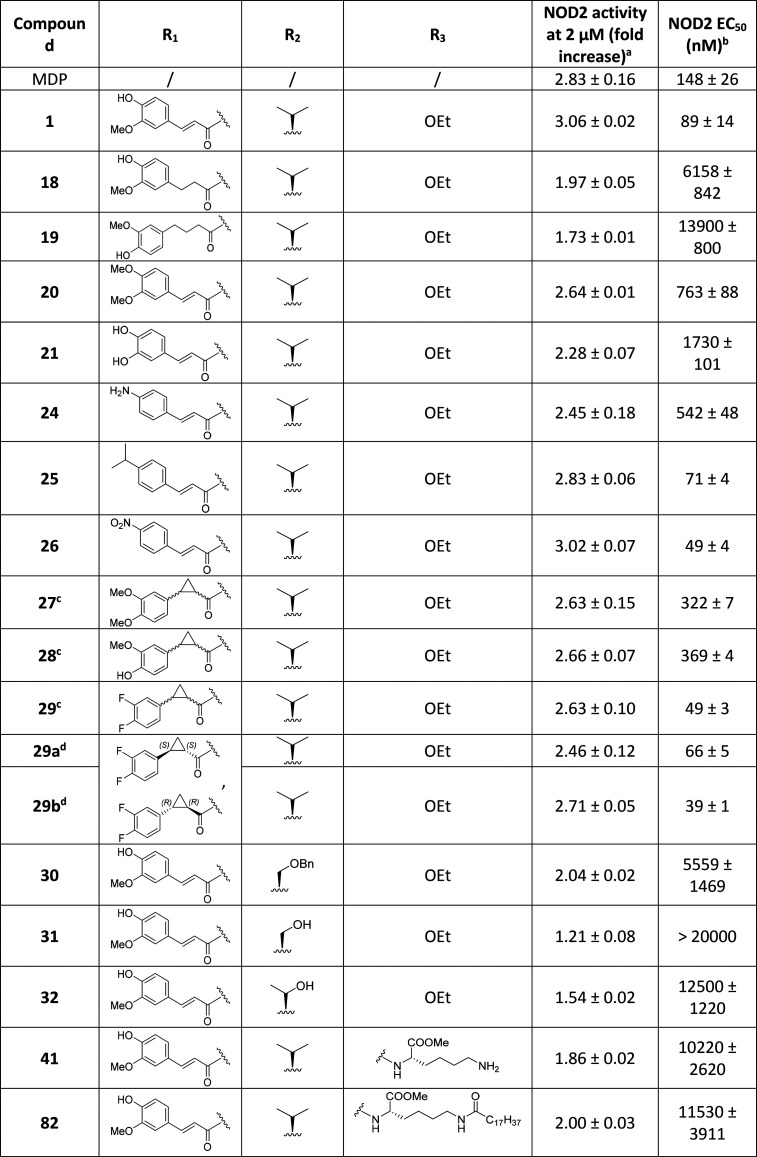
NOD2 Agonistic Activities of Compounds with Structural Modifications of the MurNAc Mimetic, the Central Amino Acid, and the Effect of Chain Elongation by l-Lysine

a SEAP activities were measured in NOD2-specific HEK-Blue cell supernatants after incubation for 18 h with MDP (2 μM) or the compounds of interest (2 μM). The data are shown as fold increases of NF-κB transcriptional activity relative to the negative control (0.1% DMSO) and are expressed as mean ± SEM of at least two independent experiments.

b EC_50_ values are expressed as mean ± SEM of at least two independent experiments with 7 or 8 concentrations used (from 1 nM to 20 μM).

c Mixture of two diastereomers (*R*,*R* and *S*,*S* configurations of substituents on the cyclopropyl ring).

d It was not possible to conclusively determine the absolute stereochemical configurations of **29a** and **29b**; however, both contained a *trans-*configured cyclopropyl ring (*R*,*R* and *S*,*S* configurations).

Recognition of MDP by NOD2 is highly stereospecific. While deviations from the l–d conformation of the l-alanine-d-isoglutamine pharmacophore can result in reduced or lack of activity, slight variations of the amino acids are permissible. For example, replacement of l-alanine with l-valine or l-serine showed comparable activity.^50−52^ Additionally, the l-threonine derivative decoupled the pyrogenic activity of MDP from its adjuvanticity.^53^ Likewise, chain elongation with l-lysine at the C-terminus, which mimics the structure of peptidoglycan from Gram-positive bacteria, achieved a similar immunostimulatory effect compared to that of MDP.^54−56^ By applying both principles to **1** (see Figure 2), derivatives were designed where l-valine was replaced by l-serine and by its more lipophilic congeners *O*-benzyl-l-serine and l-threonine (compounds **30**–**32**; Table 1), as well as by compounds where the peptide was elongated at the ω-carboxyl group of d-glutamic acid with a methyl ester of either l-lysine or *N*^6^-stearoyl-l-lysine; this latter closely resembles the structure of romurtide (compounds **41** and **82**; Table 1).

While **1** had potent activity in *in vitro* assays, it induced less pronounced increases in mouse serum titers of antigen-specific IgG upon ovalbumin immunization. We hypothesized that the disparity between the *in vitro* and *in vivo* data was a consequence of poor pharmacokinetic properties, including the metabolic instability of the ester groups. Compound **1** was suggested to be a prodrug by *in vitro* experiments, which was supported by *in silico* experiments, with the need for hydrolysis of the ethyl esters for its activation. However, if this occurs prior to **1** reaching its effector cells, this will be detrimental to its effects. We also demonstrated that the hydrolyzed free acid was less able to cross the cell membrane compared to its parent diester compound.^46^ Additionally, while MDP enters cells through the SLC15 peptide transporters and endocytosis,^57−60^ desmuramylpeptides cross the cell membrane by passive absorption to reach their target receptor NOD2 in the cytoplasm.^61^

Taking these aspects into account, we set out to modify the pharmacokinetic properties of the parent molecule, according to two different approaches. First, the introduction of lipophilic acyl groups to the carbohydrate^62^ and d-isoglutamine^63^ moieties of MDP was previously shown not only to improve the adjuvant and immunoprotective properties but also to decrease the pyrogenicity of these derivatives. Analogous transformations were applied to **1** by acylation of the phenolic hydroxyl group on the *trans*-feruloyl moiety, as well as by replacing the ethyl esters with bulkier groups, to yield a library of prodrug derivatives (**56**, **68**, **72**–**81**; Table 3). Among these, the cyclopentyl derivatives might serve a dual role. In addition to increasing the lipophilicity, chemical motifs that incorporate cyclopentyl esters have been reported to be selectively cleaved by human carboxylesterase-1, an enzyme that is restricted in expression to hepatocytes and cells of monocyte–macrophage lineage.^64^ As these latter cells express high levels of NOD2, implementation of these esterase-sensitive chemical motifs might lead to beneficial buildup of the hydrolyzed active compound only in these cells, given that charged acids would have little possibility to leave the cells.

Second, we explored the chemical space of the d-glutamic acid moiety with various mimetics of the carboxylic acid functionality. Namely, we introduced bioisosteric replacements of ester moieties with amides and esters of hydroxamic acid (compounds **57**–**64**; Table 2). Hydroxamates have previously been used as successful bioisosteric replacements of carboxylic acid groups.^65^ Amides, on the other hand, are well established for their potential as prodrugs of carboxylic acids.^66^

**Table 2:**
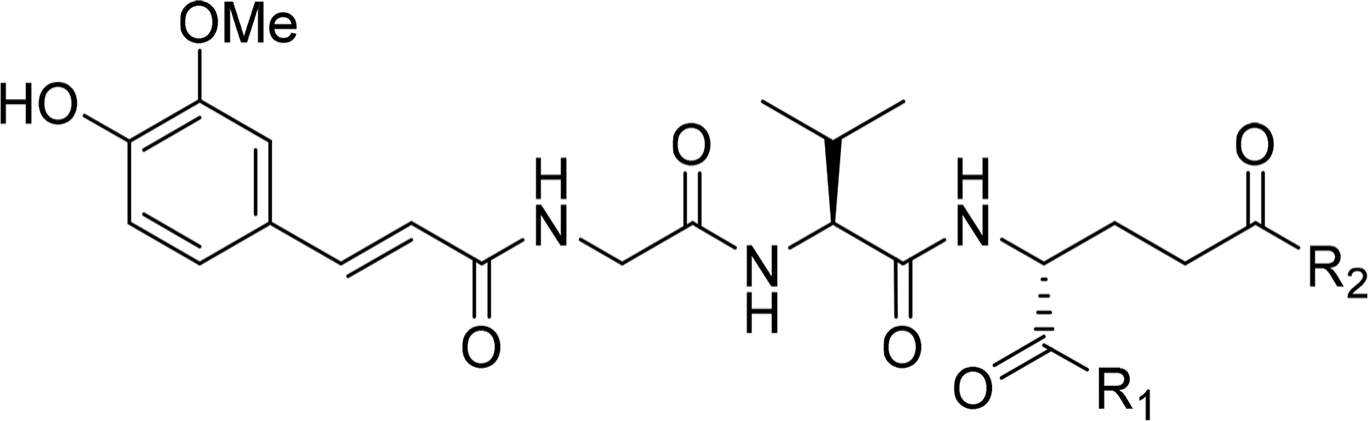

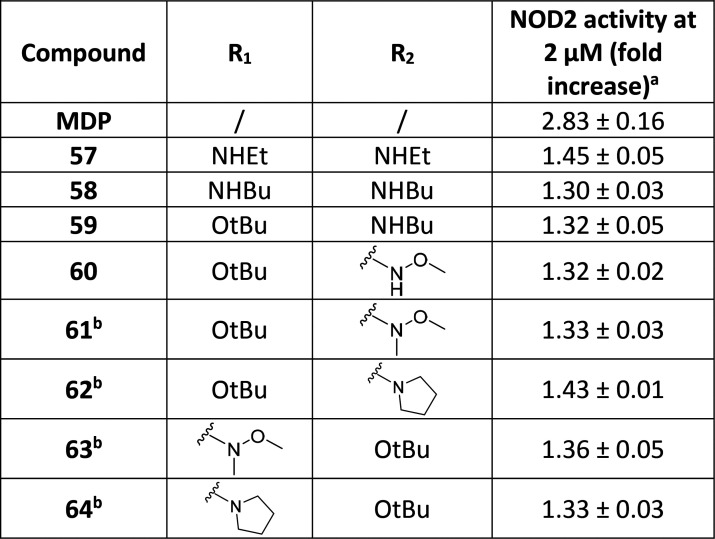
NOD2 Agonistic Activities of the Desmuramylpeptides with a Modified d-Glutamic Acid Moiety

a SEAP activities were measured in NOD2-specific HEK-Blue cell supernatants after incubation for 18 h with MDP (2 μM) or the compounds of interest (2 μM). The data are shown as fold increases of NF-κB transcriptional activity relative to the negative control (0.1% DMSO) and are expressed as mean ± SEM of at least two independent experiments.

b Mixture of two diastereomers with l and d configurations of valine.

### 2.2 Chemistry

**Scheme 1:**
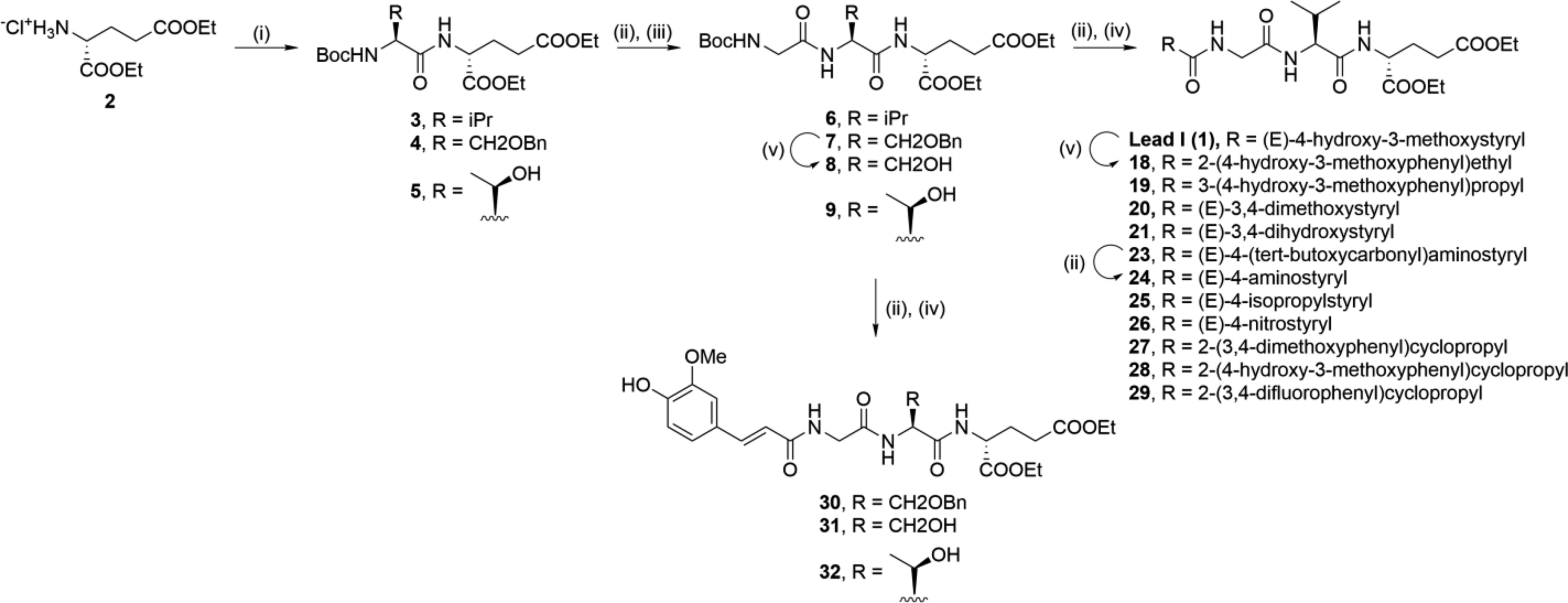
Synthesis of Compounds **1**–**32** Reagents and conditions: (i) Boc-l-Val or Boc-*O*-benzyl-l-Ser or Boc-l-Thr, DCC, HOBt, Et_3_N, DMAP, EtOAc, rt; (ii) TFA/DCM (1:5), rt; (iii) Boc-Gly, DCC, HOBt, Et_3_N, DMAP, EtOAc, rt; (iv) RCOOH, EDC, HOBt, DIPEA, DMAP, DMF, rt; (v) H_2_, Pd/C, CH_3_COOH, rt.

To prepare compounds with modifications of the *trans*-ferulic acid moiety, we designed a scalable divergent synthetic route that comprised the sequential deprotection and amide bond formation steps shown in Scheme 1. d-Glutamic acid was first esterified with thionyl chloride in ethanol, to produce the diester **2**. Coupling of **2** to commercially available Boc-protected l-valine with dicyclohexylcarbodiimide (DCC)/1-hydroxybenzotriazole (HOBt) produced the dipeptide **3**. Boc deprotection of **3** with trifluoroacetic acid (TFA) in dichloromethane (DCM) produced the deprotected TFA salt, which was immediately coupled to Boc-glycine using DCC/HOBt. Treatment of the resulting **6** with TFA produced the deprotected salt, which allowed diversification via coupling to various carboxylic acids using the 1-ethyl-3-(3-(dimethylamino)propyl)carbodiimide (EDC)/HOBt coupling strategy, to produce acyl tripeptides of **1**, **19**–**23**, and **25**–**29**. Compound **1** was additionally reduced by catalytic hydrogenation to produce the more flexible congener **18**, while **23** was deprotected with TFA to produce the free amine **24**.

Analogously, desmuramylpeptides with a modified central amino acid were synthesized by coupling **2** to commercially available Boc-protected l-serine (*O*-benzyl protected) and l-threonine, to produce dipeptides **4** and **5**. Following deprotection with TFA, DCC/HOBt coupling to Boc-glycine produced the tripeptides **7** and **9**. Catalytic hydrogenation of **7** produced the deprotected **8**, after which compounds **7**, **8**, and **9** were deprotected with TFA and coupled with *trans*-ferulic acid using the EDC/HOBt coupling strategy, to produce, in turn, the desmuramylpeptides **30**, **31**, and **32**.

**Scheme 2:**
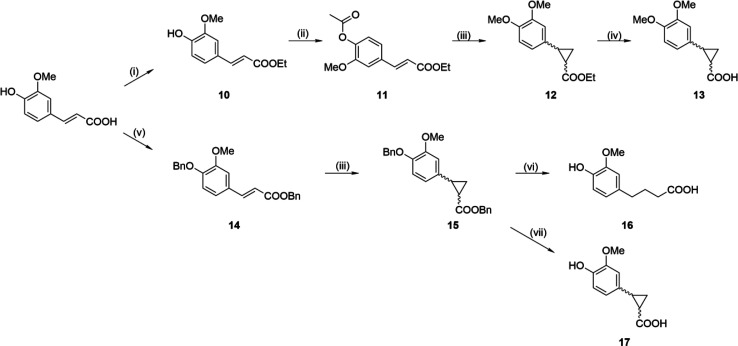
Synthesis of Cyclopropane Carrying *trans-*Ferulic Acid Derivatives Reagents and conditions: (i) SOCl_2_, EtOH, reflux; (ii) CH_3_COCl, Et_3_N, THF, rt; (iii) NaH, trimethylsulfoxonium iodide, DMSO, 50 °C; (iv) 1 M NaOH, EtOH, rt; (v) BnCl, K_2_CO_3_, DMF, 80 °C; (vi) H_2_, Pd/C, rt; (vii) Pd(OAc)_2_, Et_3_SiH, Et_3_N, DCM, rt.

Cyclopropane mimetics of *trans*-ferulic acid were synthesized as shown in Scheme 2. *trans*-Ferulic acid was protected in sequential steps, first with thionyl chloride in ethanol to produce the ethyl ester **10**, which was reacted with acetyl chloride to produce the doubly protected compound **11**. Cyclopropanation of the double bond of **11** using the Johnson–Corey–Chaykovsky reaction resulted in cleavage of the acetyl group, along with subsequent methylation of the *in situ* liberated 4-phenol group, to produce **12**, which then underwent alkaline hydrolysis, to produce the 3,4-dimethoxy derivative **13**. To avoid deprotection of the hydroxyl group, the less labile double benzyl protection was used, with *trans*-ferulic acid reacted with benzyl chloride, to produce **14**. Cyclopropanation of **14** produced **15**. Removal of the benzyl groups from **15** by catalytic hydrogenation over palladium/carbon unexpectedly resulted in the opening of the cyclopropyl ring (**16**), while a milder debenzylation method using palladium acetate, triethylsilane, and triethylamine was used to produce the desired compound, **17**.^67^

**Scheme 3:**
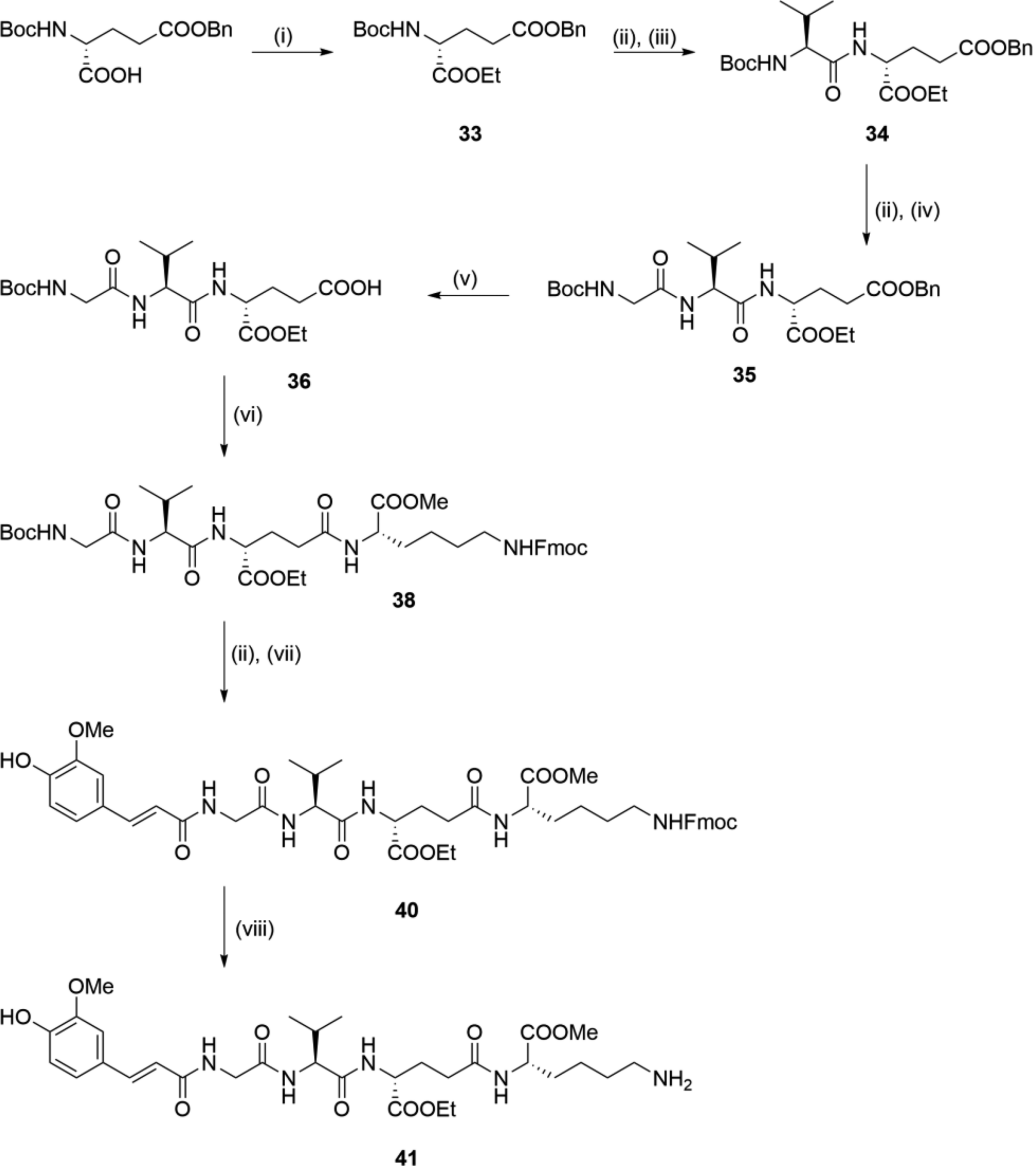
Synthesis of Compound **41** Reagents and conditions: (i) EtOH, DCC, DMAP, DCM, rt; (ii) TFA/DCM (1:5), rt; (iii) Boc-l-Val, DCC, HOBt, Et_3_N, DMAP, EtOAc, rt; (iv) Boc-Gly, DCC, HOBt, Et_3_N, DMAP, EtOAc, rt; (v) H_2_, Pd/C, EtOH, rt; (vi) **37**, COMU, DIPEA, DMF, rt; (vii) **39**, NaHCO_3_, THF/H_2_O, rt; (viii) DBU, 1-octanethiol, THF, rt.

The synthesis of compounds with an additional l-lysine residue incorporated is shown in Scheme 3. First, the 5-benzyl ester of Boc-d-glutamic acid was subjected to DCC/HOBt-mediated coupling with ethanol, to produce compound **33**. TFA-mediated cleavage of the Boc protecting group and subsequent DCC/HOBt coupling was performed twice, first with Boc-l-valine, and then with Boc-glycine, to produce the tripeptide **35**. Following cleavage of the benzyl ester of **35** with catalytic hydrogenation, the free acid **36** was coupled to Fmoc-protected l-lysine (**37**; Supporting Information, Scheme S1) using (1-cyano-2-ethoxy-2-oxoethylidenaminooxy)dimethylamino-morpholino-carbenium hexafluorophosphate (COMU) as the coupling reagent, to produce **38**. Then **38** was converted to **40** via a two-step sequence that involved cleavage of the Boc protecting group with TFA, and subsequent coupling to the *N*-hydroxysuccinimide-activated *trans*-ferulic acid **39** (Supporting Information, Scheme S2). Finally, Fmoc deprotection of the tetrapeptide **40** under alkaline conditions generated by 1,8-diazabicyclo[5.4.0]undec-7-ene (DBU) in tetrahydrofuran (THF), with 1-octanethiol acting as the dibenzofulvene scavenger,^68^ produced the desired desmuramylpeptide **41**.

**Scheme 4:**
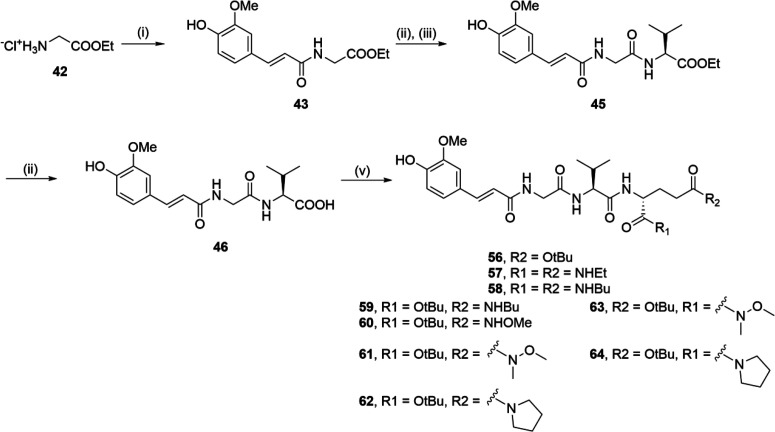
Synthesis of Compounds **56**–**64** Reagents and conditions: (i) *trans*-ferulic acid, EDC, HOBt, DIPEA, DMAP, DMF, rt; (ii) 1 M NaOH, EtOH, rt; (iii) **44**, EDC, HOBt, DIPEA, DMAP, DMF, rt; (v) **47**–**55**, EDC, HOBt, DIPEA, DMAP, DMF, rt.

Assembly of the desmuramylpeptides with modifications of the d-glutamic acid moiety was carried out as shown in Scheme 4. Glycine was first esterified with thionyl chloride in ethanol to produce the ethyl ester **42**, which was then coupled to *trans*-ferulic acid using the EDC/HOBt coupling strategy. Next, the *N*-feruloyl-glycine **43** produced was deprotected with alkaline hydrolysis, and subsequently coupled with the ethyl ester of l-valine (**44**) using EDC/HOBt, to produce the acyl dipeptide **45**. Alkaline hydrolysis of **45** produced the free acid **46**. Diversification by EDC/HOBt-mediated coupling to various d-glutamic acid derivatives (**47**–**55**; Supporting Information, Scheme S3) produced the desmuramylpeptides **56**–**64**, with incorporated carboxylic acid bioisosteres.

**Scheme 5:**
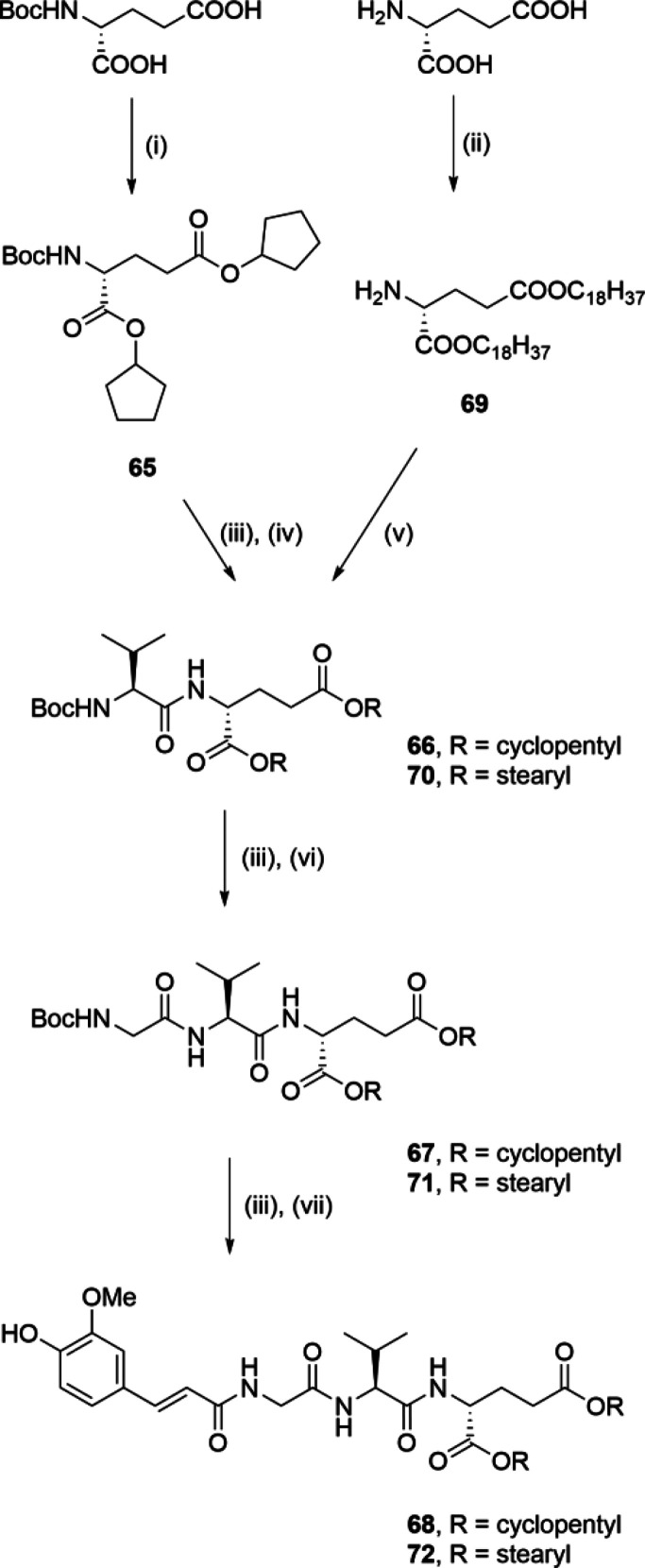
Synthesis of Compounds **68** and **72** Reagents and conditions: (i) cyclopentanol, EDC, DMAP, DCM, rt; (ii) 1-octadecanol, pTsOH, toluene, reflux; (iii) TFA/DCM (1:5), rt; (iv) Boc-l-Val, DCC, HOBt, Et_3_N, DMAP, EtOAc, rt; (v) Boc-l-Val, EDC, HOBt, DIPEA, DMAP, DCM, rt; (vi) Boc-Gly, DCC, HOBt, Et_3_N, DMAP, EtOAc, rt, or Boc-Gly, EDC, HOBt, DIPEA, DMAP, DCM, rt; (vii) *trans*-ferulic acid, EDC, HOBt, DIPEA, DMAP, DMF or DCM, rt.

A synthetic procedure similar to that described in Scheme 1 was used for the synthesis of lipophilic esters **68** and **72** (shown in Scheme 5). Boc-d-glutamic acid was first esterified with cyclopentanol using EDC to produce the corresponding diester **65**. Following Boc deprotection with TFA, the ensuing coupling to Boc-protected l-valine produced the dipeptide **66**. Similarly, acid-catalyzed esterification of d-glutamic acid with 1-octadecanol produced the diester **69**, which was coupled to Boc-l-valine to produce the dipeptide **70**. Compounds **66** and **70** were then subjected to two iterative cycles of TFA-mediated Boc deprotection with consecutive coupling, first to Boc-glycine, to produce **67** and **71**, and ultimately to *trans*-ferulic acid, to produce the ester congeners of **1**, **68** and **72**.

**Scheme 6:**
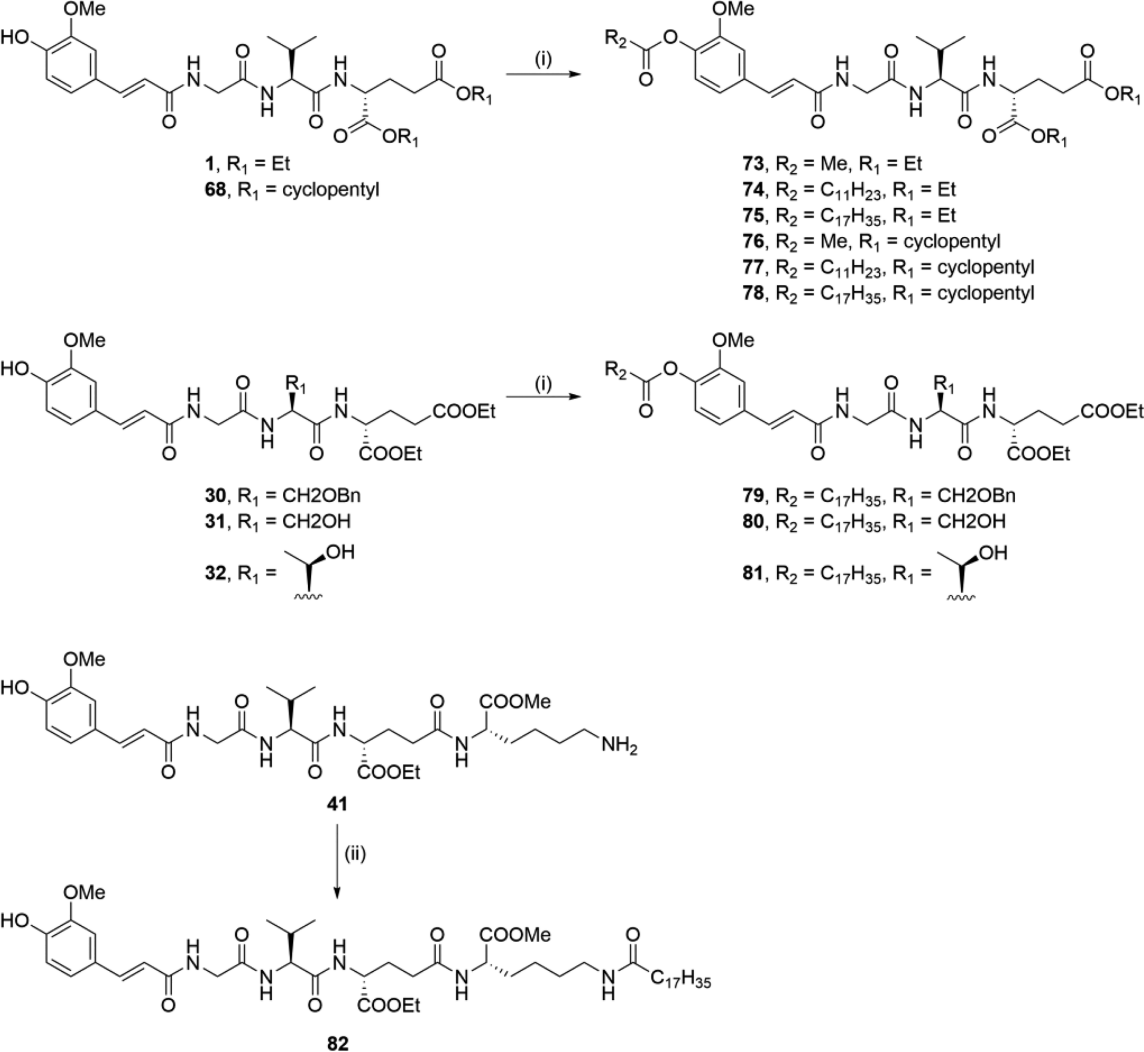
Synthesis of Compounds **73**–**82** Reagents and conditions: (i) RCOCl, Et_3_N, THF, rt; (ii) C_17_H_35_COCl, Et_3_N, DMF, rt.

Compounds **1**, **30**, **31**, **32**, **41**, and **68** were further acylated with acyl chlorides of varying chain lengths in the presence of triethylamine to produce the lipophilic ester derivatives **73**–**82** (Scheme 6).

### 2.3 Biological Studies

#### 2.3.1 NOD2 Agonistic Activity of Synthesized Desmuramylpeptides

To determine the NOD2 agonistic potential of the synthesized desmuramylpeptides at the cellular level, we used the validated and commercially available HEK-Blue NOD2 cell line reporter assay. HEK-Blue NOD2 cells were first treated for 18 h with MDP, **1**, or the novel desmuramylpeptides at 2 μM. The NF-κB transcriptional activity measured was normalized to that of the vehicle treated (0.1% DMSO) control HEK-Blue NOD2 cells. The compounds that showed significant activity at 2 μM were further assayed for their dose-dependent activities, for determination of their EC_50_ values. None of the tested compounds were cytotoxic toward the HEK-Blue NOD2 cells at the highest tested concentration (20 μM), as determined by the (3-(4,5-dimethylthiazol-2-yl)-5-(3-carboxymethoxyphenyl)-2-(4-sulfophenyl)-2*H*-tetrazolium) (i.e., MTS) cell viability assay (Supporting Information, Figure S1).

Exploration around the chemical space of the cinnamoyl moiety of the parent compound **1** (EC_50_ = 89 nM) produced compounds **18**–**21** and **24**–**28** (Table 1). Increased flexibility through the reduction of the double bond (**18**; EC_50_ = 6.16 μM) resulted in decreased NOD2 agonistic activity by a factor of 70. Similarly, the NOD2 agonistic activity was decreased by a factor of 156 with the spacer prolonged to a propylene group (**19**; EC_50_ = 13.9 μM), which indicated that **1** provides the optimal positioning of the aromatic ring. The 4-hydroxy-3-methoxy substitution pattern of the aromatic ring in **1** was also seen to be advantageous; namely, the 3,4-dimethoxy (**20**; EC_50_ = 763 nM), 3,4-dihydroxy (**21**; EC_50_ = 1.73 μM), and 4-amino (**24**; EC_50_ = 542 nM) derivatives all showed lower activities. Interestingly, the 4-isopropyl (**25**; EC_50_ = 71 nM) and the 4-nitro (**26**; EC_50_ = 49 nM) derivatives showed similar NOD2 agonistic activity to **1**, despite their contrasting electronic properties, which indicated that the interactions with the protein of the group in the 4-position are primarily of a hydrophobic nature and are related to the size of the substituent. A nitro-substituted cinnamic acid was previously used in the design of MDP-C, which is a potent *in vivo* NOD2 agonist; however, in contrast to **26**, the nitro group was attached to the d-glutamic acid moiety.^69^ Cyclopropanation of the alkene moiety in the structure of the 3,4-dimethoxy derivative **20** produced **27** (EC_50_ = 322 nM) with a 2.4-fold improved potency over **20**, which suggests that the cyclopropane ring assists in directing the aromatic ring to a more favorable position. Surprisingly, by applying the same concept to **1**, which thus produced **28** (EC_50_ = 369 nM), the potency was reduced by a factor of 4. The ^1^H–^1^H NOESY spectra revealed that both **27** and **28** retained the *trans* orientation of the substituents on the cyclopropane ring (Supporting Information, Figures S7 and S8). This analysis, however, did not differentiate between the two *trans*-configured diastereomers (i.e., carrying the *R*,*R* and *S*,*S* configurations on the cyclopropyl ring), and the relative levels of those two species in the mixture could not be determined. Considering that it is likely that only one of them binds ideally to NOD2, changes in their proportions would influence the EC_50_ determined, which provides a possible explanation of the disparate data obtained after cyclopropanation of **1** and **20**. Our previous study also identified **29**, a cyclopropyl derivative with a 3,4-difluoro substituted aromatic ring.^46^ With an EC_50_ of 49 nM, **29** was the most potent derivative in the cyclopropyl series. With the aim to ascertain the potencies of individual diastereomers, we subjected **29** to chiral HPLC resolution (Supporting Information, Figures S4, S5, and S6), which yielded very low quantities of its pure diastereomers **29a** (EC_50_ = 66 nM) and **29b** (EC_50_ = 39 nM), with a relatively small difference between their NOD2 activities. ^1^H–^1^H NOESY analysis revealed that both **29a** and **29b** contained a *trans*-configured cyclopropyl ring (Supporting Information, Figure S9); however, as for **27** and **28**, it was not possible to determine their absolute stereochemical configurations here. Nonetheless, as this approach provided only marginal improvements in the NOD2 activity, we posited that their chiral resolution would serve no direct purpose here and that the diastereomeric mixtures of cyclopropane featuring derivatives do not need to be separated.

Next, we examined the effects of modifications to the amino-acid structure (**30**–**32**, **41**, **82**; Table 1). The MDP analogs with l-threonine and l-serine were previously shown to be suitable substitutes for the l-alanine, as demonstrated by their retained *in vivo* adjuvant activities;^50,51^ however, these substitutions have yet to be evaluated in the context of desmuramylpeptides. Interestingly, desmuramylpeptides with both l-threonine (**32**; EC_50_ = 12.5 μM) and l-serine (**31**; EC_50_ > 20 μM) showed relatively poor activities. *O*-Benzylation of l-serine (**30**; EC_50_ = 5.56 μM) slightly improved the activity, perhaps indicating the importance of the bulky nature of this amino-acid side chain. Lower activity was also seen when the peptide chain was extended by l-lysine (**41**; EC_50_ = 10.2 μM). This might be attributable to decreased membrane permeability at physiological pH, due to the presence of an ionized amine group; however, amidation of this group with a lipophilic stearoyl chain (**82**; EC_50_ = 11.5 μM) did little to improve the activity. Similar data were obtained in a study by Effenberg et al. (2017), where norAbuMDP-Lys-L18 (a derivative of MDP with stearoyl-l-lysine) showed reduced potency compared to MDP, despite the resemblance of both compounds to romurtide.^70^

Exploration of the chemical space around the d-glutamic acid moiety produced desmuramylpeptides **57**–**64** (Table 2). Both functionalized carboxamates and amides were evaluated as potential bioisosteric replacements of the carboxylic acid functionality. When compared to the 2.83-fold and 3.06-fold NOD2 activations of MDP and **1** in a single point assay, with respect to untreated control cells, these compounds showed considerably diminished NOD2 agonistic activity at 2 μM (1.30-fold to 1.45-fold NOD2 activation). These data are in agreement with the previously reported impaired activities of MDP derivatives that have either a diamidated d-isoglutamine moiety^52^ or a lipophilic amide attached to the α position of d-isoglutamine.^71^ Slightly better activities were expected for the derivatives with hydroxamate, given that hydroxamic acids are readily hydrolyzed to their corresponding carboxylic acids.^72^ However, it is worth noting that HEK293 cells have low hydrolytic activity due to low expression of carboxylesterases, the enzymes involved in the hydrolysis of both hydroxamic acids and amides.^73,74^ Higher susceptibility of ester groups to both enzymatic and spontaneous hydrolytic processes might explain the superior effects of **1** and other ester derivatives in the HEK-Blue NOD2 cell assays. Nonetheless, given that direct bioisosteric replacement of both ethyl ester groups with their ethyl amide counterparts resulted in substantially diminished activity of **57**, these data provide confirmation of our previous *in vitro* and docking studies that suggested that the **1** ester moieties predominantly assist in compound internalization and do not contribute to the NOD2 binding.^46^

These data encouraged us to introduce the last group of modifications, in which we retained the original pharmacophore of **1** and increase its lipophilicity by introducing cleavable ester groups of varying sizes to the cinnamoyl and d-glutamic acid moieties. As the cellular assays are defined by both the crossing of the membrane by the compounds and their activation of NOD2, the lipophilicity of these compounds will have a major role in their cellular NOD2 activities. The phenolic hydroxy group served as a useful attachment point for the introduction of acetyl (C_2_), lauroyl (C_12_), and stearoyl (C_18_) groups through esterification. Likewise, the two ethyl ester functional groups were readily replaced with bulkier cyclopentyl, *tert*-butyl, and stearyl groups. The NOD2 agonistic activities of the resulting prodrug derivatives are summarized in Table 3.

**Table 3:**
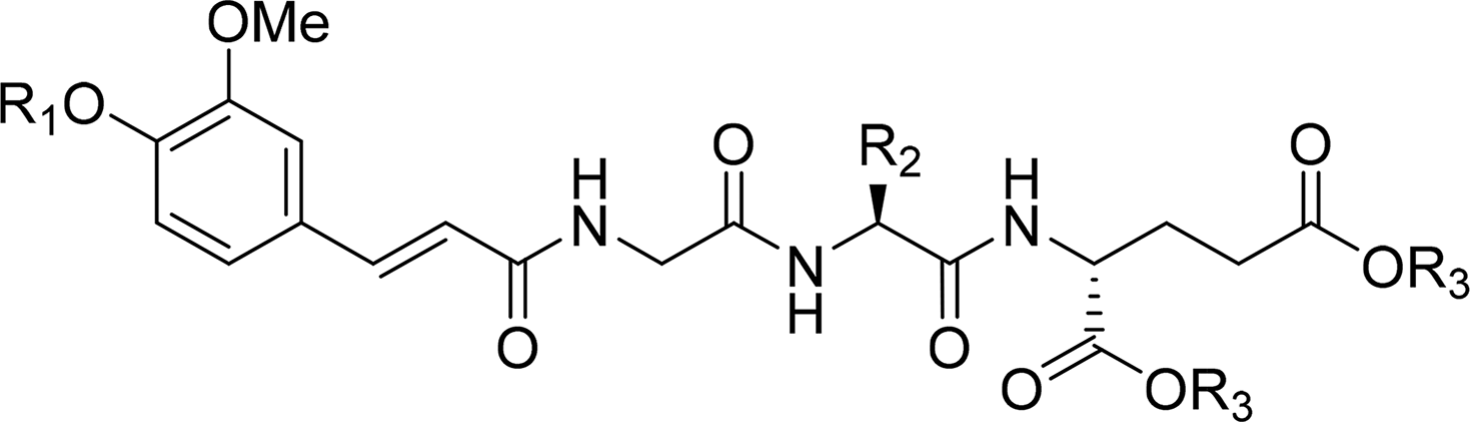

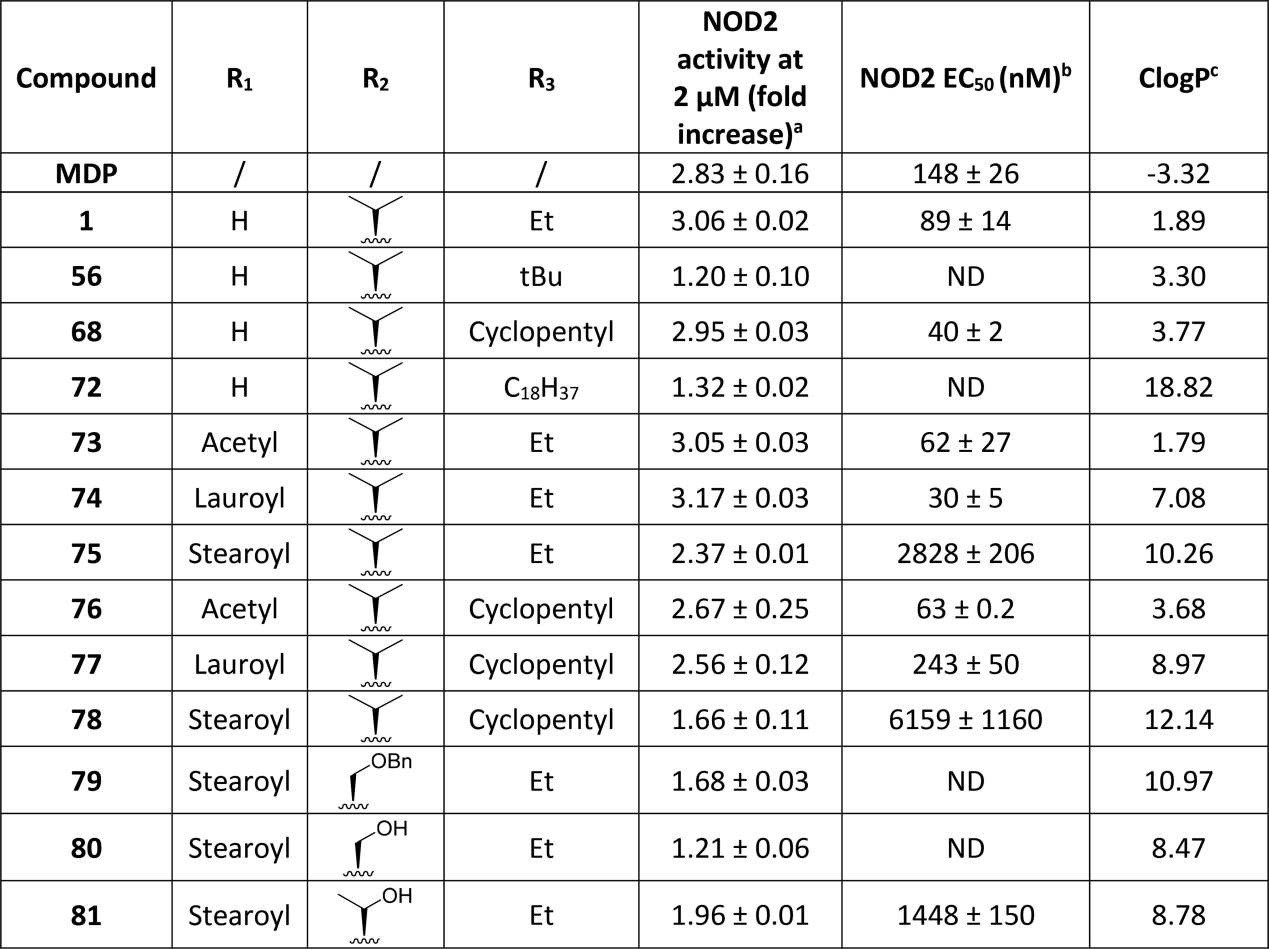
NOD2 Agonistic Activities of the Lipophilic Desmuramylpeptides

a SEAP activities were measured in NOD2-specific HEK-Blue cell supernatants after incubation for 18 h with MDP (2 μM) or the compounds of interest (2 μM). The data are shown as fold increases of NF-κB transcriptional activity relative to the negative control (0.1% DMSO) and are expressed as mean ± SEM of at least two independent experiments.

b EC_50_ values are expressed as mean ± SEM of at least two independent experiments with 7 or 8 concentrations used (from 1 nM to 20 μM). ND, not determined.

c ClogP values as calculated by the ChemDraw software.

**Figure 3:**
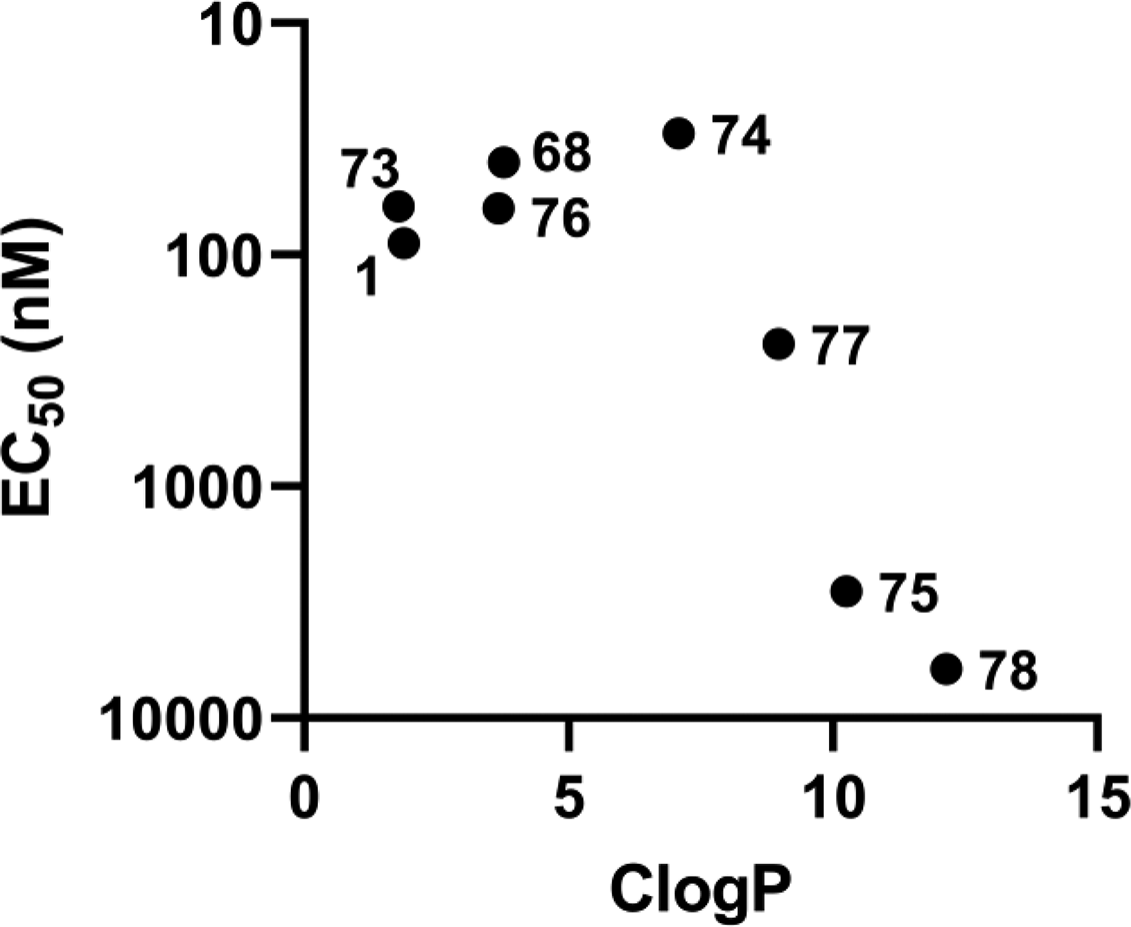
ClogP–EC_50_ relationship of the compound **1** prodrug derivatives.

Of note, there was an inverted U-shape correlation between the compound NOD2 activities and their lipophilicities (as calculated logP [ClogP] values; Figure 3). Namely, the desmuramylpeptides with ClogP in the 1.7 to 3.8 range showed increasing NOD2 activation, where compound **68** had an EC_50_ of 40 nM, an over 2-fold improvement over **1** (EC_50_ = 89 nM). Compound **74**, a derivative with a lauroyl tail on the aromatic ring, was the most active NOD2 agonist of this series (EC_50_ = 30 nM), despite a significantly increased ClogP of 7.08. Interestingly, lauric acid (C_12_) was previously shown to activate NOD2 and induce IL-8 secretion from HCT116 colon epithelial cells.^75^ To determine whether the increased activity of **74** here is a result of the release of lauric acid after hydrolysis, the HEK-Blue NOD2 cells were also treated with lauric acid, both alone and in combination with **1**. In contrast to previous indications, lauric acid did not show any NOD2 activation alone, and it did not enhance the NOD2 agonistic activity of **1** (data not shown). Further increases in the lipophilicity resulted in a sharp drop in the *in vitro* NOD2 agonistic activity. Extending the C_12_ chain of **74** to a longer C_18_ chain, which produced **75** (EC_50_ = 2.83 μM), resulted in markedly diminished activity, by a factor of 90, although the stearoyl group is a structural motif that has been used repeatedly in the preparation of potent MDP derivatives.

We postulated that the differences in NOD2 activation here can be attributed to more facile cleavage of the shorter acetyl and lauroyl esters, compared to the longer stearoyl esters, by the intracellular enzymes in the HEK293 cell line used. We thus expected that the introduction of the acetyl, lauroyl, and stearoyl groups to the structure of the dicyclopentyl congener **68** would follow the same activity trend, with the lauroyl derivative additionally improving the activity of **68**. Unexpectedly, the resulting C_12_ counterpart **77** (EC_50_ = 243 nM) showed a sixth of the activity of **68**. As exemplified by **78** (EC_50_ = 6.16 μM), extending the lipophilic tail to C_18_ further abrogated the NOD2 activation. These data demonstrate that NOD2 activation by these compounds is most likely linked directly to their lipophilicity. This is further supported by the NOD2 agonistic activities of the two acetylated derivatives, the diethyl ester **73** (EC_50_ = 62 nM), and its dicyclopentyl surrogate **76** (EC_50_ = 63 nM). These two maintained similar ClogP values and consequently retained the EC_50_ values of their congeners with a free phenolic hydroxy group. Finally, replacing both of the ethyl groups of **1** with stearyl esters (i.e., **72**) almost completely abrogated the NOD2 agonistic activity. Similar structure–activity relationships were identified previously and attributed to the different interactions of the lipophilic MDP analogs with the biomembranes. While higher lipophilicity facilitated membrane permeability, it also increased the association of the compounds with the membrane.^76^ The retention of the lipophilic derivatives in the membrane might explain their reduced effects in the cell assays here, as they will then not be available to bind to NOD2, which is located in the cytoplasm. Conversely, these same effects might be beneficial and lead to enhanced activities of such derivatives in *in vivo* assays, especially when liposomes are used as the lipophilic delivery system.

As expected, similarly reduced NOD2 activation was shown when a stearoyl chain was introduced into the structure of **30** (2.04-fold activation at 2 μM) to produce **79** (1.68-fold), a derivative with *O*-benzyl-l-serine replacement of the l-valine. However, when a stearoyl chain was introduced into **31** (1.21-fold) to produce **80** (the l-serine based analog; 1.21-fold) there was almost no change to the NOD2 activation, while when a stearoyl chain was introduced into **32** (the l-threonine based analog; EC_50_ = 12.5 μM) to produce **81** (EC_50_ = 1.45 μM), this showed a 10-fold improvement in potency.

Compound **56** provided a notable deviation from the ClogP–EC_50_ relationship, where there were two *tert*-butyl ester groups on the d-glutamic acid. *tert*-Butyl esters are considerably less hydrolyzable by intracellular esterases, and especially by human carboxylesterase-1. On the other hand, cyclopentyl esters were previously shown to be excellent substrates for hydrolysis by human carboxylesterase-1.^64^ Given the low carboxylesterase activity of HEK293 cells, it is no surprise that **56** resulted in markedly reduced NOD2 activation, despite its favorable ClogP (3.30).

As NOD2 and NOD1 agonists share certain structural characteristics, we wanted to determine whether these compounds selectively target NOD2. Selectivity against NOD1 of MDP and all of these synthesized desmuramylpeptides was analyzed in an analogous assay with the HEK-Blue NOD1 cell line. None of the compounds tested showed any significant activity at 2 μM, which thus confirmed their selectivity for NOD2 (Supporting Information, Figure S2).

Furthermore, the specificities were determined by pretreating HEK-Blue NOD2 cells with a previously reported NOD2 antagonist^77^ prior to stimulation with MDP (2 μM) or the desmuramylpeptides (2 μM). The resulting NF-κB-induced SEAP activities were compared to those of the controls without the NOD2 antagonist pretreatment. The comparative reduction in NF-κB transcriptional activities indicated that the effects of these desmuramylpeptides are due to their NOD2 activation (Supporting Information, Figure S3).

We note here that the readouts from the HEK-Blue cell assay system might not accurately represent the behaviors of these synthesized desmuramylpeptides under *in vivo* conditions. Thus, further biological evaluations were carried out for the most potent *in vitro* NOD2 agonists, **68**, **73**, and **74**, also with the inclusion of two derivatives with a stearoyl group, **75** and **81**. The C_18_ lipophilic tail was shown to be beneficial for development of MDP derivatives with activities *in vivo*, in part due to its anchoring in the membrane of the liposomes^78^ used as the delivery system of choice for the *in vivo* applications of NOD2 agonists. As the experimental design included the investigation of *in vivo* adjuvant activities of NOD2 agonists encapsulated in a liposomal formulation, inclusion of **75** and **81** in further testing was thus warranted, despite their lower NOD2 activation in the HEK-Blue NOD2 cell assays.

#### 2.3.2 Immunostimulatory Effect of Desmuramylpeptides on PBMCs

The immunostimulatory effects of the selected desmuramylpeptides were evaluated using human primary peripheral blood mononuclear cells (PBMCs). This heterogeneous mixture of immune cells allowed the study of the effects of these NOD2 agonists in a physiologically relevant system, where other NOD2-interacting and downstream signaling proteins were present. The effects of desmuramylpeptides were first determined for the secreted cytokine profile using a cytometric bead array cytokine kit. Moreover, considering that activation of NOD2 is also an important amplification signal for Toll-like receptor (TLR)-induced inflammatory responses,^79−81^ we also examined the effects on cytokine secretion of the combination of desmuramylpeptides with LPS, a well-known TLR4 agonist.

**Figure 4:**
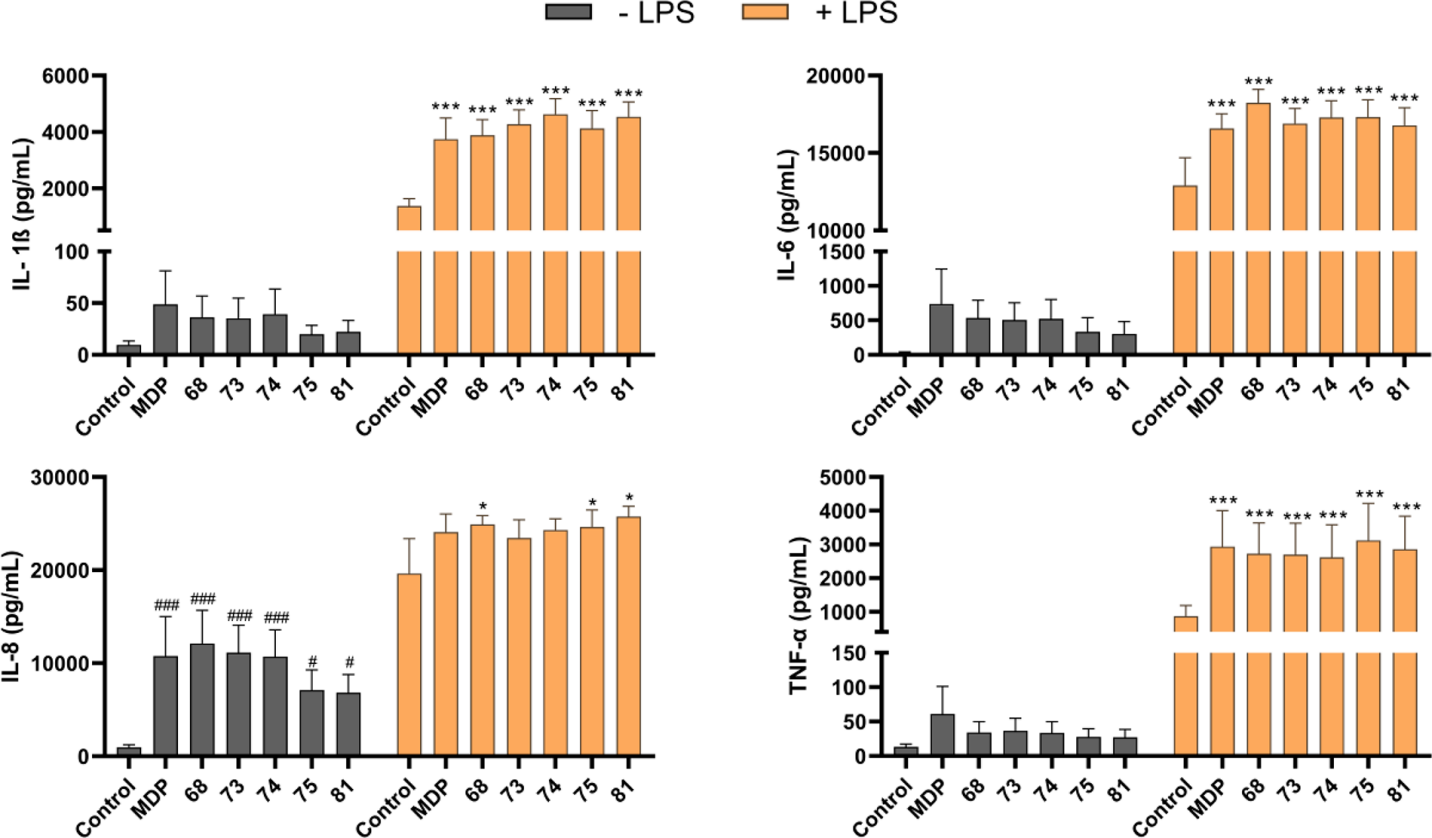
Effects of the MDP and desmuramylpeptide treatments on the release of cytokines from human PBMCs. Cytokine concentrations were measured after 18 h stimulation with MDP (2 μM) or the desmuramylpeptides (2 μM) in the absence or presence of LPS (10 ng/mL). Data are expressed as mean ± SEM of 4 independent experiments. ^#^*p* < 0.05, ^###^*p* < 0.001 versus untreated controls; *p < 0.05, **p < 0.01, ***p < 0.001 versus LPS-treated PBMCs.

Figure 4 shows the effects of the desmuramylpeptides (2 μM) on induction of the cytokines IL-1β, IL-6, IL-8, and TNF-α, both alone and in combination with LPS (10 ng/mL). These data show similar structure–activity relationships to the data obtained in the SEAP reporter gene assays. Compounds **68**, **73**, and **74** by themselves induced small increases in the levels of all four of these cytokines, with similar effects to MDP, while **75** and **81** induced lower responses. In line with previous studies, the effects of MDP and the desmuramylpeptides were most pronounced for the induction of the chemokine IL-8.^82^

Stimulation with LPS resulted in large increases in cytokine production in general, which were further enhanced by MDP and the desmuramylpeptides. When compared to stimulation by LPS alone, all of the tested compounds significantly increased the levels of IL-1β, IL-6, and TNF-α. These effects were synergistic; i.e., the IL-1β, IL-6, and TNF-α produced upon combined desmuramylpeptide and LPS stimulation were higher than the sum of the effects from these individual immunostimulants. This is in agreement with the previously described synergistic signal amplification between NOD2 and TLR4.^83,84^ On the other hand, the IL-8 produced appeared to plateau following the combined stimulation with LPS and the NOD2 agonists, which thus indicated the saturation of IL-8 production in these PBMCs.

Among the diverse PBMC subpopulations, natural killer (NK) cells express high levels of functional NOD2.^85^ As innate immune cells, NK cells have a central role in immunosurveillance, by their detection and destruction of virus-infected and cancer cells, through both direct cytolytic activity and release of cytokines, which can further facilitate the recruitment and activation of other innate and adaptive immune cell types. Stimulation of NK cells with MDP activates the NF-κB signaling pathway, which results in expression and release of TNF-α and interferon (IFN)-γ, as the key regulators of the Th1 cellular immune response.^85^ This is further enhanced synergistically by IL-12 and IFN-α, which suggests a role for accessory-cell-derived cytokines in the formation of an optimal NK cell response.^86^ MDP-stimulated NK cells also show enhanced cytotoxicity toward the Tu167 squamous cell carcinoma of the head and neck cell line.^85^ Furthermore, monocytes represent ∼10% of PBMCs, and they have a similar nonspecific cytolytic activity against cancer cells, which is potentiated after MDP stimulation, both alone and in combination with IFN-γ.^87,88^

In the present study, we examined these desmuramylpeptides in terms of potentiation of the cytotoxic activity of the PBMCs against cancer cells. To this end, we used a previously described flow-cytometry-based cytotoxicity assay where PBMCs are co-incubated with fluorescently labeled cancer cells.^89^ As indicated above, the NOD2-mediated NK cell activity originates from both the direct effects of NOD2 agonists on NK cells and the indirect activation through cytokine release by other NOD2-responsive cells, including monocytes. Thus, we used the whole PBMC population as the effector cells, rather than isolated NK cells, to provide conditions that would be closer to the *in vivo* environment, wherein the immune responses can be reinforced by interactions between the distinct immune cell subpopulations. Two malignant cell lines were used as the target cells in a 40:1 effector to target cell ratio: MEC1 B-chronic lymphocytic leukemia cells, and K562 chronic myelogenous leukemia cells. The K562 cells are considered a “classic” NK cell target, as they lack the expression of major histocompatibility complex (MHC) class I that is required for NK cell inhibition.^90^

**Figure 5:**
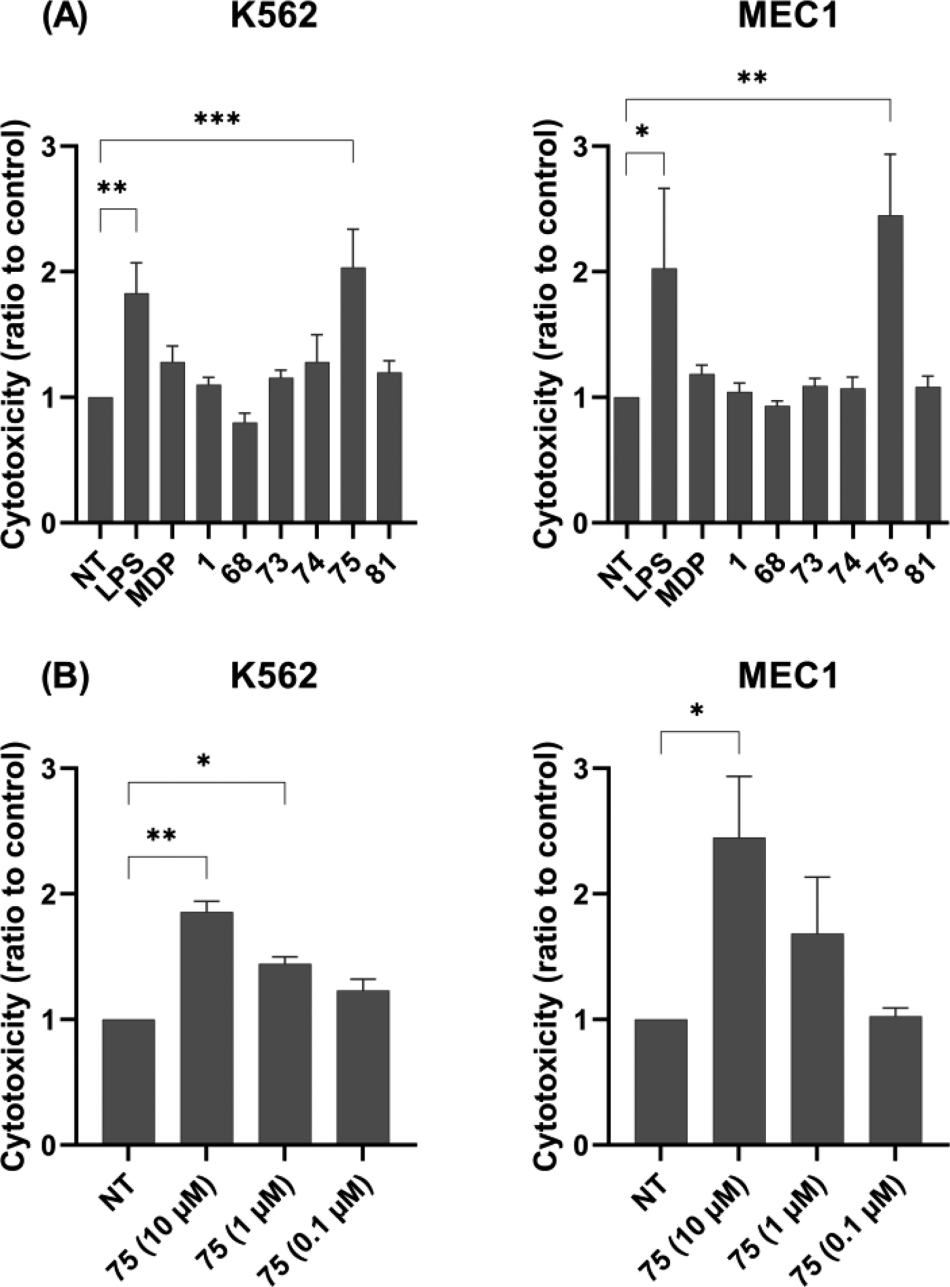
Effects of MDP and desmuramylpeptides on the cytotoxic activities of PBMCs against K562 and MEC1 cells. (A) PBMCs were treated for 18 h with MDP (10 μM), desmuramylpeptides (10 μM), or LPS (1 μg/mL) before the addition of the K562 or MEC1 cells. Cytotoxicity was determined after 4 h co-incubation. (B) Concentration-dependent effect of **75** on the induction of PBMC cytotoxicity. Data are shown as activities relative to the negative control (NT, 0.1% DMSO) and are expressed as mean ± SEM of three (MEC1) or four (K562) independent experiments. *p < 0.05, **p < 0.01, ***p < 0.001 versus relevant negative controls.

Interestingly, apart from LPS as the positive control,^91^ only **75** showed any significant boost to the cytotoxicity of the PBMCs against both of these cancer cell lines at 10 μM (Figure 5A). At 1 μM, this augmented cytotoxicity was still significant against the K562 cells, but not against the MEC1 cells, while at 100 nM, no effects were seen against either cell line (Figure 5B). These data are markedly in contrast with the data obtained in the HEK-Blue NOD2 cell line, where **75** had one-hundredth the NOD2 agonistic activity compared to MDP, **1**, **68**, **73**, and **74**. As the desmuramylpeptides **73**, **74**, and **75** belong to a series of **1** prodrug derivatives that contain increasing lengths of lipophilic acids on the aromatic ring (i.e., acetic, lauric, stearic acids), these data indicated that the long lipophilic tail might be a contributing factor for PBMC cytotoxicity. Conversely, **81** also has a stearoyl tail but was devoid of activity; however, the generally weaker NOD2 agonism of l-threonine based desmuramylpeptides compared to l-valine derivatives has to be borne in mind. In parallel, the PBMCs and cancer cells were also treated with the desmuramylpeptides alone, to define any direct cytotoxicity they might have toward the PBMCs or cancer cells. None of these resulted in increased proportions of dead cells, thereby confirming that the enhanced activity of **75** can be attributed to the stimulation of PBMC cytotoxic activity (data not shown).

Similar lipophilicity-dependent effects on immune cell stimulation were described by Kalyuzhin et al. (1996).^76^ An MDP derivative with a C_7_ lipophilic tail was shown to be a potent stimulator of T cells, macrophages, and NK cells, with the NK cell activity seen as increased cytotoxicity against YAC-1 lymphoma cells. On the other hand, a C_16_ MDP derivative showed suppressive effects on the function of lymphocytes, except for the release of IL-1 and TNF-α, which was comparable after stimulation for both of these MDP derivatives. Based on these data, where the tested desmuramylpeptides induced similar levels of cytokines in PBMCs, the large differences in their cytotoxicity activation suggest that there is no linear dependence between lipophilicity and immunostimulatory effects in the various PBMC cell subpopulations. As for the HEK-Blue NOD2 cell assay, these effects might be ascribed to the hydrolipophilic balance and its effects on the interactions of these compounds with biomembranes.^76^ Further studies are required to determine the physicochemical properties that are optimal for the induction of NK cell activity. Nonetheless, given the previously reported link between NOD2-dependent NK cell activation and *in vivo* antitumor activity,^92^ the data from the present study demonstrate the potential of these desmuramylpeptides in NK cell-dependent cancer immunotherapeutic approaches.

Furthermore, we carried out an analogous cytotoxicity assay on macrophages that were produced by differentiation of THP-1 cells using phorbol 12-myristate 13-acetate, for 3 days.^93^ Interestingly, the desmuramylpeptide treatments did not enhance the tumoricidal activities of the resulting M0 macrophages against the MEC1 cancer cell line (data not shown). THP-1-derived macrophages have been reported to express functional NOD2; however, in line with our findings, MDP treatment of these cells did not result in their activation, as shown by the unchanged levels of their secreted cytokines.^85^

**Figure 6:**
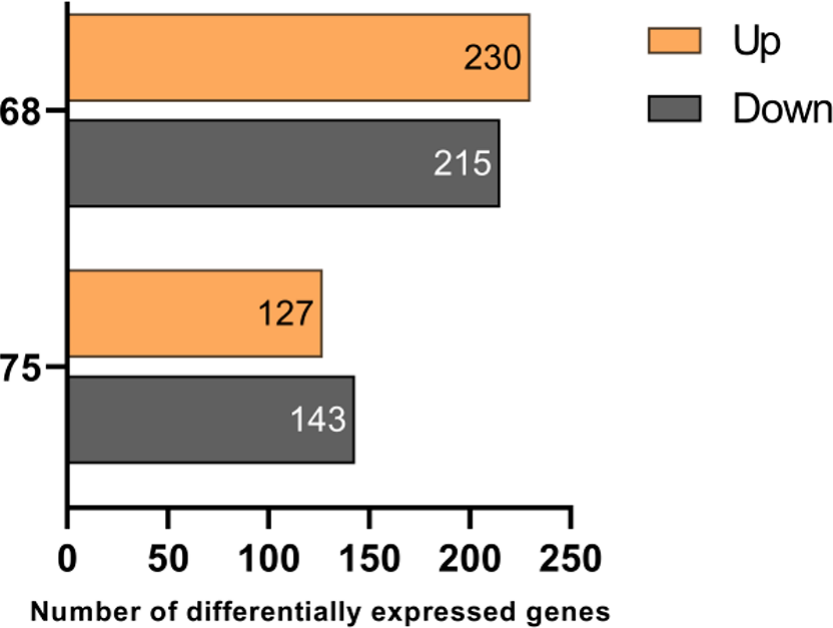
Significantly up-regulated and down-regulated genes in PBMCs from three independent donors after 18 h of treatment with **68** and **75** (2 μM) treatment. A false discovery rate cutoff of <0.05 and a gene expression fold-change >1.5 or <0.667 compared to the untreated control (0.1% DMSO) was applied.

Based on the potent *in vitro* NOD2 activation by **68** and the PBMC cytotoxicity activation by **75**, we explored the effects of **68** and **75** in terms of induction at the transcriptional level. Next-generation sequencing of RNA isolated from PBMCs was carried out following their stimulation for 18 h with **68** or **75** (2 μM). After applying a low expression filter, the remaining 19046 genes from the desmuramylpeptide-treated samples were compared to the vehicle-treated controls (0.1% DMSO). As shown in Figure 6, **68** significantly modulated the expression of 445 genes (230 up-regulated, 215 down-regulated, compared to the control), while **75** modulated the expression of 270 genes (127 up-regulated, 143 down-regulated, compared to the control). Differential expression analysis between the **68**- and **75**-treated samples, however, did not show any significant differentially expressed genes (data not shown). This thus indicates that **68** and **75** induced similar transcriptional changes, where those of **75** were of lower magnitude, which is consistent with the weaker *in vitro* potency of **75** compared to **68**.

**Figure 7:**
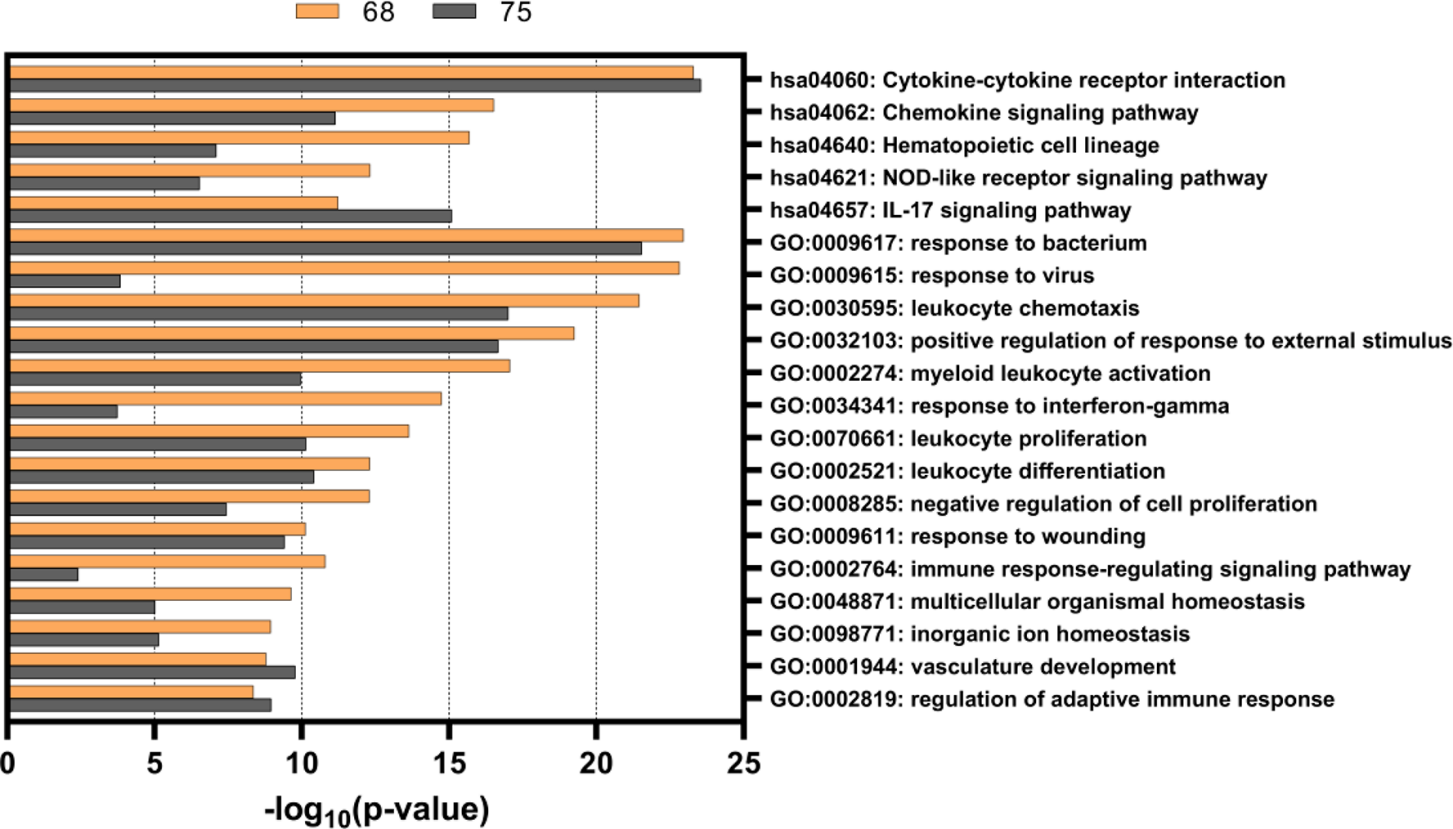
Top 20 most-enriched Gene Ontology (GO) biological processes and Kyoto Encyclopedia of Genes and Genomes (KEGG) terms. The differentially expressed genes after **68** and **75** treatments (2 μM) were used as input in pathway enrichment analysis using Metascape.^94^

**Figure 8:**
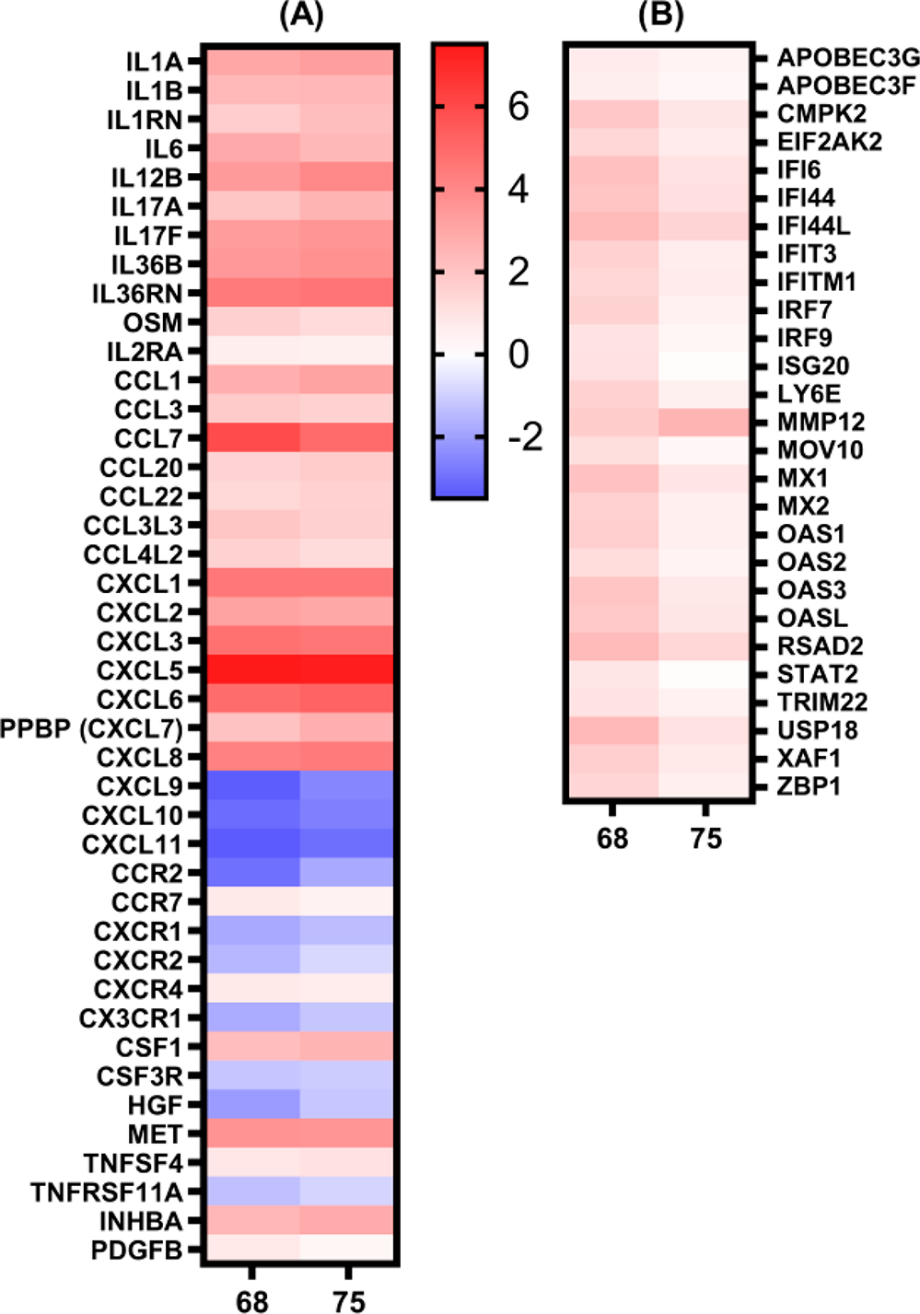
Heat maps of the (A) log_2_(fold change) of the differentially expressed genes in the “Cytokine-cytokine receptor interaction” KEGG pathway and (B) log_2_(fold change) of significantly modulated interferon-stimulated genes (ISGs) by **68** and **75**.

To explore the functional implications of the observed transcriptional changes, gene enrichment analysis was performed for the differentially expressed genes after the **68** and **75** treatments. This was based on the Gene Ontology (GO) biological processes database and the Kyoto Encyclopedia of Genes and Genomes (KEGG) database. The analysis was performed using Metascape, which is a web-based annotation tool that is distinguished from other similar tools by hierarchical clustering of the overlapping enrichment terms.^94^ Each cluster is then represented only by its most significant term, thus removing other redundant terms within each specific cluster. The analysis of the **68** and **75** induced transcriptomes revealed significant enrichment of several pathways involved in immune responses (Figure 7). The pathway that was most significantly overexpressed by both compounds was the KEGG pathway “Cytokine-cytokine receptor interaction” (hsa04060), within which there was up-regulation of IL1B, IL6, and CXCL8 (IL-8), which confirms the observations made at the protein level (see Figure 4). Compounds **68** and **75** also induced the transcription of proinflammatory IL-1 cytokines and their antagonists (IL1A, IL36B, IL1RN, IL36RN), IL-17 cytokines (IL17A, IL17F), oncostatin M (member of the IL-6 cytokine family), and IL12B (p40 subunit of IL-12) (Figure 8A). Furthermore, there was marked up-regulation of several CC (CCL1, 3, 7, 20, 22, 3L3, 4L2) and CXC (CXCL1, 2, 3, 5, 6, 7, 8) chemokines. Indeed, a study on the transcriptional signatures of 23 different TLR/NOD-like receptor agonists (including murabutide as a representative NOD2 agonist) by Salyer and David (2018) identified strong up-regulation of CC and CXC chemokines as a shared characteristic of the majority of the innate immune stimulants they tested.^95^ CXCL1, 2, 3, 5, 6, 7, and 8 are primarily responsible for neutrophil trafficking, as these bind to the cognate receptor CXCR2, and thus contribute to neutrophil-mediated inflammation.^96^ Interestingly, while the up-regulation of IL12B, IL17A, and IL17F indicated a Th1/Th17 type response, **68** and **75** both induced the down-regulation of Th1-associated chemokines CXCL9, CXCL10, and CXCL11. These three chemokines share the same CXCR3 receptor that is mainly expressed on Th1 and NK cells, and they are predominantly involved in IFN-γ-driven Th1 immune responses. Conversely, the up-regulated CCL1 and CCL22 are generally considered to be indicative of Th2 responses.^96^ The shift toward Th2-type responses was further substantiated by up-regulation of the co-stimulatory molecule TNFSF4 (also known as OX40 ligand [OX40L]). Notably, the activation of the OX40L–OX40 axis has previously been linked to NOD2 ligand-driven Th2 polarization.^97^

While **68** and **75** induced similar transcriptional changes in regards to cytokines and chemokines, there were pronounced differences in the induction of genes related to the defense response against viral pathogens. Specifically, there was a **68**-mediated up-regulation of several interferon-stimulated genes (ISGs), which, conversely, were modulated by **75** to a lesser degree (Figure 8B). Their transcription is triggered through the JAK-STAT pathway following recognition of IFNs by their cognate receptors. Several of the upregulated ISGs, such as EIF2AK2 (protein kinase R), IFITM1, ISG20, MX1, OAS1, and RSAD2 (viperin) were previously featured for their antiviral effector functions.^98^ Importantly, the activity of endogenously induced type I IFNs and their respective ISGs was also found to be a key step in the development of adaptive immune responses induced by a wide range of currently used vaccine adjuvants. Their effects are especially essential in the formation of Th1-type responses by promoting the differentiation of Th1 cells and the induction of cytotoxic T lymphocytes, thus generating antiviral and antitumor protective immunity.^99,100^ A meta-analysis of the transcriptional profiles induced by several different vaccines linked LY6E, MX1, OAS3, IFI44L, IFI6, and IFITM3 to the early phases after vaccination.^101^ With the exception of IFITM3, all of these genes were significantly up-regulated by **68**. It is worth noting that while we did observe the up-regulation of IFN downstream signaling proteins, such as the transcription factors STAT2, IRF7, and IRF9, we did not detect the transcription of type I IFN mRNA. However, it is known that the expression of IFNs is both induced and shut off rapidly, to ensure swift immunoprotective effects prior to the onset of detrimental effects to the host.^102^

#### 2.3.3 *In Vitro* Adjuvant Properties of Desmuramylpeptides **68** and **75**

Dendritic cells have an instrumental role in bridging the innate and adaptive arms of immunity by processing and presenting antigens to naive T cells, to thus generate antigen-specific T-cell and B-cell immune responses.^103^ As DCs express a wide range of PRRs, including NOD2, they serve as critical target cells for adjuvant functions. NOD2 activation in DCs was previously shown to increase expression of co-stimulatory molecules (e.g., CD80, CD86, CD40) and production of inflammatory cytokines (e.g., TNF-α, IL-6, IL-8, IL-12), indicators of DC maturation and activation.^104,105^ NOD2 engagement by MDP in DCs also induces autophagy, a vital process in the delivery of cytosolic proteins for MHC class II antigen presentation, which ultimately leads to induction of CD4^+^ T helper cell responses.^18,19^ Depending on the nature of the stimuli involved in DC activation, these cells respond with the production of different cytokines, which leads to the polarization of T helper cells toward distinct effector functions.^103^ Among these, the Th2 and Th1 subtypes are known to promote antigen-specific humoral and cellular immunity, respectively. Cellular immune responses are further characterized by induction of cytotoxic CD8^+^ T cells, which are instrumental in protective immunity against intracellular pathogens and tumors. Notably, NOD2 activation functions as an effective signal for DC-mediated cross-priming of cytotoxic CD8^+^ T cells, through up-regulation of MHC class I-dependent antigen cross-presentation pathways.^106,107^

To evaluate the adjuvant potential of the two most-promising desmuramylpeptides, **68** and **75**, in terms of their effects on DC-mediated activation of CD4^+^ and CD8^+^ T cells, we used an *in vitro* antigen-presentation assay with C57BL/6 mouse bone-marrow-derived DCs (BMDCs). Following stimulation with **68** and **75** (1 or 10 μM) in the presence of ovalbumin soluble protein (50 μg/mL), BMDCs were cocultured with carboxyfluorescein succinimidyl ester (CFSE)-labeled naive ovalbumin-specific CD4^+^ or CD8^+^ T cells, which were isolated from splenocytes of OT II and OT I transgenic mice, respectively. After 3 days of coculture, CD25 (α subunit of IL-2 receptor)^108^ expression was determined, along with the CFSE dilution, as markers of T-cell activation and proliferation, respectively. Furthermore, we also examined how this desmuramylpeptide-induced priming of T cells affected the secreted cytokine profiles in BMDC/T-cell cocultures.

**Figure 9:**
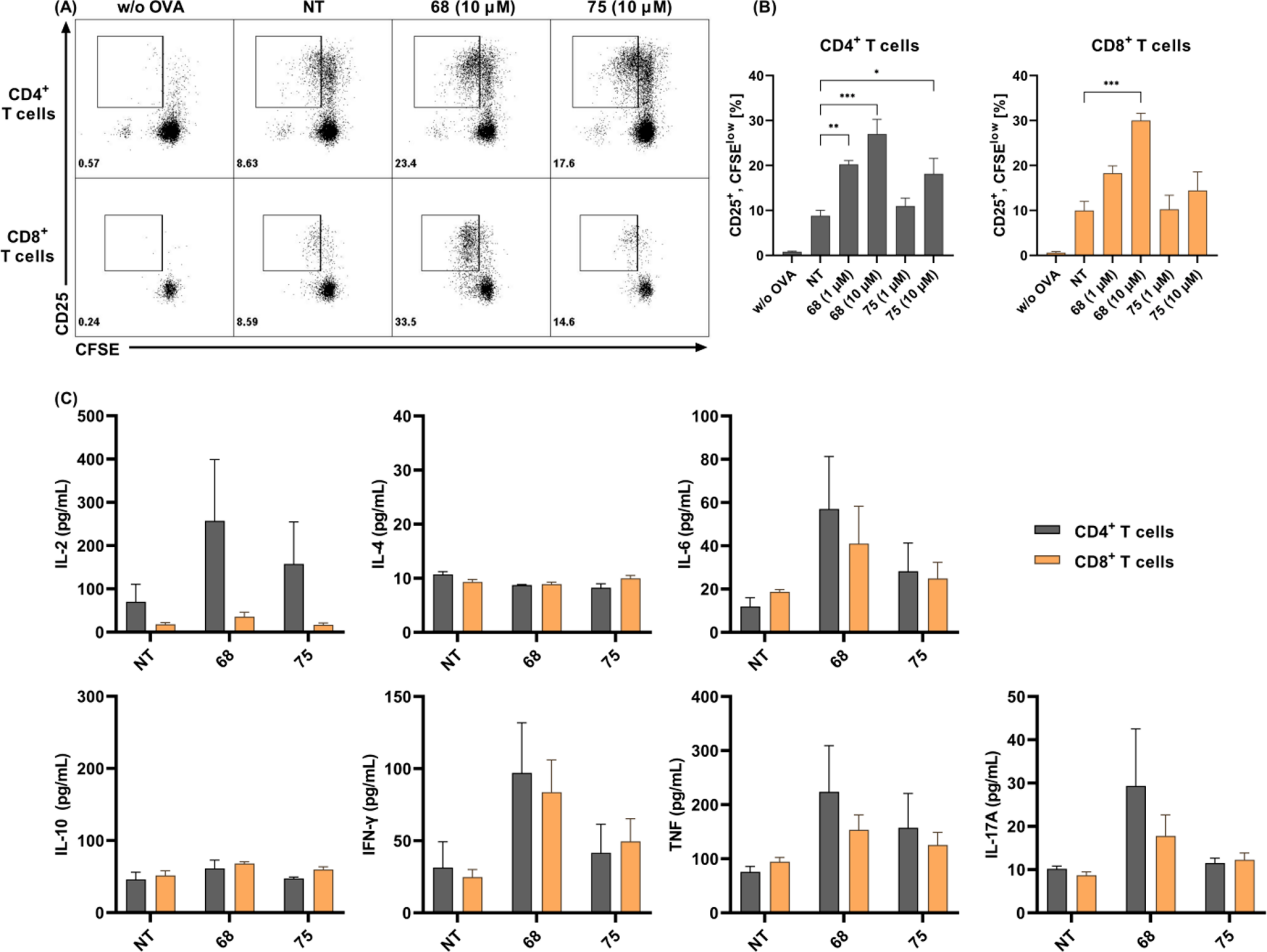
CD4^+^ and CD8^+^ T cell activation, proliferation, and cytokine secretion in response to ovalbumin presentation by bone-marrow-derived dendritic cells (BMDCs) pretreated with desmuramylpeptides **68** and **75**. BMDCs from C57BL/6 mice were treated with **68** and **75** (1 and 10 μM) and ovalbumin (50 μg/mL). After 18 h, they were washed and cocultured for 72 h with carboxyfluorescein succinimidyl ester (CFSE)-labeled ovalbumin-specific CD4^+^ or CD8^+^ T cells, isolated from OT II or OT I mouse splenocytes, respectively. (A) Representative dot plots of live Thy1.2^+^/CD4^+^ or Thy1.2^+^/CD8^+^ T cells, showing CD25 expression and CFSE dilution. (B) Quantification of the proportions of CD25^+^, CFSE^low^ T cells. The analysis regions are shown in panel A. (C) Cytokine concentrations in coculture supernatants following co-incubation with BMDCs as described in panel A. Data are expressed as mean ± SEM of duplicates of two independent experiments. *p < 0.05, **p < 0.01, ***p < 0.001.

As illustrated in Figure 9A,B, with **68** and **75** at 10 μM, both significantly enhanced BMDC-mediated CD4^+^ T-cell activation and proliferation. In line with its superior *in vitro* NOD2 activation, **68** also showed this effect at 1 μM. Enhanced T-cell activation by **68**, and to a lesser degree by **75**, was additionally characterized by elevated levels of IL-2, IL-6, IFN-γ, TNF-α, and IL-17A (Figure 9C). The effects of **68** were most pronounced for the production of IL-2, a pleiotropic cytokine that in addition to driving CD4^+^ T-cell growth also augments the activity of CD8^+^ and NK cells. Activated CD4^+^ T cells produce large amounts of IL-2, although Th1 cells are generally considered to be the major source of IL-2.^109^ IFN-γ is characterized by its inductive effects on CD8^+^ T cells, macrophages, and NK cells, and its up-regulation of MHC I and MHC II antigen presentation pathways in DCs, and it is similarly associated with a switch toward a Th1-like immune response. Taken together with the unaffected levels of Th2-associated IL-4 and IL-10, these data indicate that **68** induced a primarily Th1 polarized response. Interestingly, NOD2 activation by MDP was previously identified as a driving force toward Th2 polarization, with reduced levels of IFN-γ and increased production of IL-4.^110^ This previous study, however, used human-monocyte-derived DCs, while the present study used BMDCs from C57BL/6 mice, a strain that is generally considered as Th1 dominant.^111^ Further studies on other mice strains and human DCs would thus be required to accurately determine the Th1/Th2 polarization promoted by the desmuramylpeptides.

Compound **68** also significantly increased the BMDC processing and cross-presentation of ovalbumin to CD8^+^ T cells, which resulted in their enhanced activation, proliferation, and cytokine secretion. It has been shown that activation of NOD1 and NOD2 in a similar *in vitro* cross-priming assay described by Asano et al. (2010) translated into enhanced proliferation of IFN-γ-secreting CD8^+^ T cells *in vivo*, which resulted in increased antigen-specific antitumor and antibacterial cytotoxic activities.^106^ Complementary data in intranasally immunized mice were also reported for adamantylamide dipeptide, another representative of the desmuramylpeptide class of adjuvants.^112^ As most of the currently licensed adjuvants almost exclusively induce antibody responses, there is a pressing need for adjuvants that can induce cellular immunity, especially in the cancer immunotherapy field. Based on the data described above, **68** holds great potential in this respect.

#### 2.3.4 *In Vivo* Adjuvant Properties of Desmuramylpeptides **68** and **75** in Ovalbumin-Induced Antibody Responses

There is clear evidence that NOD2 activation translates into adjuvant activities *in vivo*.^31,52,113^ Due to the unfavorable pharmacokinetic and toxicologic properties of MDP, significant effort has been devoted to the development of more tolerable MDP derivatives. During this research, *in vivo* experimental data have revealed that both lipophilicity of the derivative and context of application influence the intensity and type of the provoked immune response. For example, MDP applied in saline induces a predominantly Th2-biased humoral immune response.^15^ On the other hand, lipophilic MDP derivatives promote a Th1-biased cellular immune response, especially when used in conjunction with lipophilic carrier systems such as liposomes.^70,114^ Notably, liposomes have repeatedly been featured in the development of vaccines, due to their versatility, biocompatibility, and enhancing effects on the generation of immune responses.^115^

**Figure 10:**
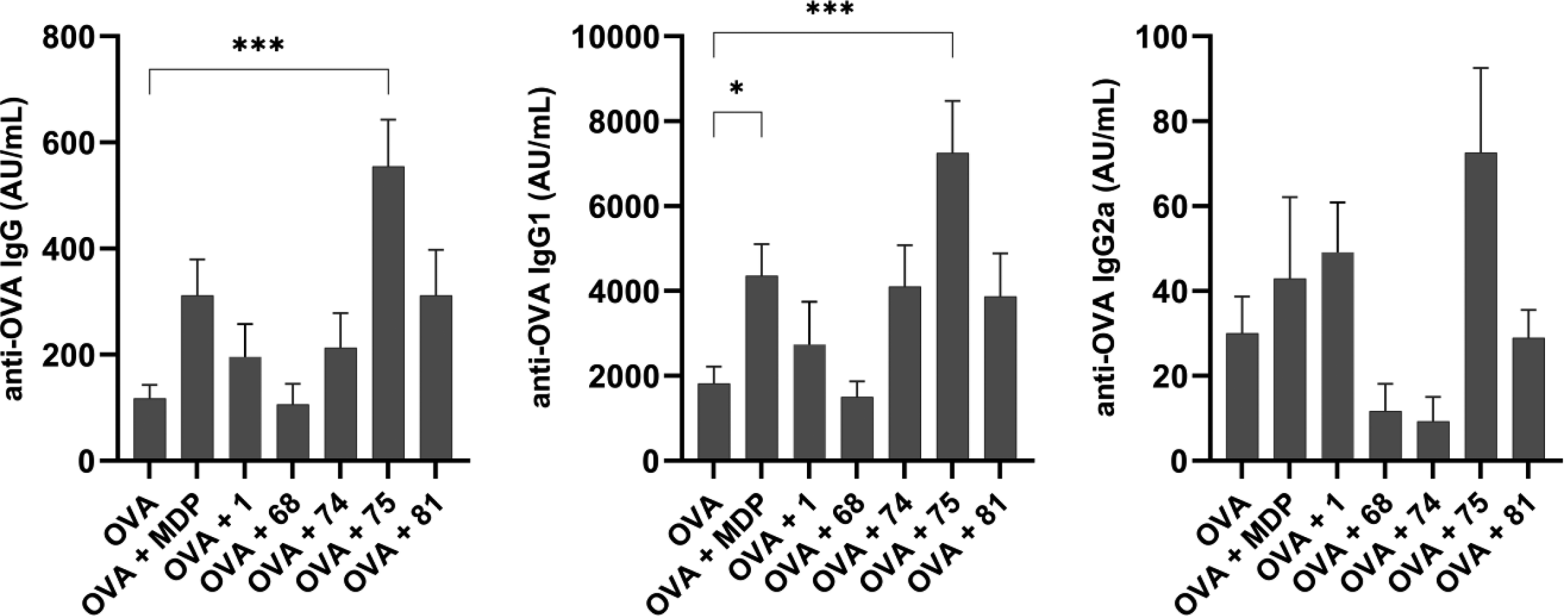
Ovalbumin-specific IgG (left), IgG1 (middle), and IgG2a (right) responses in NIH/OlaHsd mice after immunization with ovalbumin-loaded neutral liposomes (10 μg of ovalbumin per dose), with adjuvants MDP or the desmuramylpeptides (0.30 μmol of adjuvant per dose). The concentrations were measured 1 week after the booster dose. Data are expressed as mean ± SEM of 5 mice per group. *p < 0.05; ***p < 0.001.

To determine whether enhanced antigen presentation *in vitro* is correlated to *in vivo* adjuvant activities, an immunization study was performed in a murine model of adjuvanticity. Specifically, the selected desmuramylpeptides were investigated for induction of systemic immune responses against the model antigen ovalbumin. Seven groups of NIH/OlaHsd mice were immunized with ovalbumin-containing neutral liposomes, either alone or with additional adjuvants of MDP or the desmuramylpeptides **1**, **68**, **74**, **75**, or **81**. After immunization and one booster dose, the mice sera were collected and assayed for ovalbumin-specific IgG antibodies (Figure 10).

As expected, due to the low immunogenicity of ovalbumin, the ovalbumin-loaded liposomes without added adjuvants induced weak systemic responses and thus served as the negative controls. In agreement with our previous experiments, MDP moderately enhanced the production of anti-ovalbumin IgG antibodies, while **1** showed only marginal adjuvant activity.^46^ On the other hand, there was a significant 5-fold boost in the elicited IgG responses in the group immunized with the addition of **75**, followed by a 3-fold boost by **81**, where both of these derivatives had a C_18_ lipophilic tail on the aromatic ring. The C_12_-carrying derivative **74** showed similar activity to **1**, while **68**, although it was the most potent *in vitro* NOD2 agonist, was devoid of adjuvant activity under these conditions.

To understand the nature of these induced immune responses in terms of Th1/Th2 polarization, the levels of the Th1-associated IgG1 and Th2-associated IgG2a antibody isotypes were also measured (Figure 10).^116^ In all of the experimental groups, the levels of induced anti-ovalbumin IgG1 closely resembled the total IgG levels; however, there were notable differences in the desmuramylpeptide enhancement of production of IgG2a. Consistent with previous reports, MDP induced a predominantly Th2-biased response,^15,117^ with significant increases in IgG1 generation and only marginal increases in IgG2a, compared to the control without adjuvant. Likewise, a largely IgG1-based response was elicited by **1** and **81**, while in the groups immunized by **68** and **74**, there was slight suppression of the IgG2a response. Finally, immunization with the liposomes containing the **75** adjuvant significantly enhanced ovalbumin-specific IgG1 responses and, importantly, also induced the highest levels of IgG2a antibodies, which indicated a shift toward a more balanced Th1/Th2 response.

It is clear that the *in vivo* activities of the desmuramylpeptides do not follow the same structure–activity relationship rules as the *in vitro* activities. Given that **1**, **68**, **74**, and **75** act as prodrugs of the same active compound, their differential effects on the induction of humoral immune responses appear to originate from their distinctive physicochemical properties. Indeed, an evident increase in the adjuvant activities correlated with the addition of a lipophilic C_18_ tail on the aromatic ring, as for **75** and **81**.

Entrapment and subsequent retention of both antigens and adjuvants are important considerations in the design of liposomal vaccines. The entrapment of soluble MDP in liposomes was previously shown to be problematic, due to the diffusive escape with a short half-life of retention of 5 h.^118^ Many lipophilic MDP derivatives have been designed to allow their loading into liposomes to be increased, to provide increased adjuvant activity and reduced side effects. For example, addition of lipophilic adamantane groups to peptidoglycan fragments was shown to assist in the anchoring of these derivatives into the liposomal lipid bilayer, using NMR spectroscopy.^119^ Likewise, straight-chain lipophilic anchors can be used for the same purpose, due to the extensive network of van der Waals interactions that they can form in the lipophilic bilayer of liposomes. Surprisingly, a reduction in chain length from C_18_ to C_16_ was shown to significantly increase the propensity for the undesired escape of lipids from liposomal membranes.^78^ Analogously, the enhanced *in vivo* activities of **75**, and to a lesser extent of **81**, compared to their C_12_ congener **74** and the compounds that lacked a lipophilic anchor appeared to be due to their more efficient liposome encapsulation and subsequent retention, thus facilitating their uptake by antigen-presenting cells. Stable incorporation into liposomes additionally protects adjuvants from the actions of hydrolytic enzymes. Given that desmuramylpeptides rely on passive absorption to cross the cell membrane,^61^ extracellular hydrolysis would severely hamper their activation of the cytosolic NOD2.

One of the key advantages of liposomes is their versatility. The chemical properties of the lipid components and the preparation procedures can be chosen to modulate charge, size, size distribution, entrapment, and location of antigens and adjuvants, all of which can potentially influence the intensity and form of the immune response against the antigen of interest.^115^ The net surface charge of liposomes, in particular, was shown to significantly influence the entrapment efficiency of the antigen^120^ and the interactions of the liposomes with antigen-presenting cells and other endogenous tissue components.^121^ Moreover, surface modifications of liposomes can be exploited for targeted delivery of antigens and adjuvants to specific immune-cell populations, to thus promote the desired immune responses while avoiding adverse effects due to targeting of irrelevant cell types. Among the potential targets, mannose receptors are highly expressed on the surface of DCs and macrophages. Liposomes decorated with mannose receptor ligands (i.e., mannosylated liposomes) enhance the uptake and activation of DCs, to result in amplified immune responses against the encapsulated antigen.^122^ Additionally, MDP-containing mannosylated liposomes have been shown to be effective for inhibition of liver metastasis through their targeted delivery to tumoricidal macrophages. This thus expands the potential of these formulations to cancer immunotherapies.^123^

To determine whether the adjuvanticity of **75** can be modulated through changes to the liposomal formulation, a second *in vivo* experiment was designed in which the adjuvant activities of three different liposomal compositions were compared. Neutral liposomes were prepared from egg phosphatidylcholine and cholesterol. Addition of the anionic dicetylphosphate resulted in liposomes with a negative net surface charge, while monomannosyl–poly(ethylene glycol)–palmitic acid derivative (Man-PEG-Pam) was added to assemble mannosylated liposomes.

**Figure 11:**
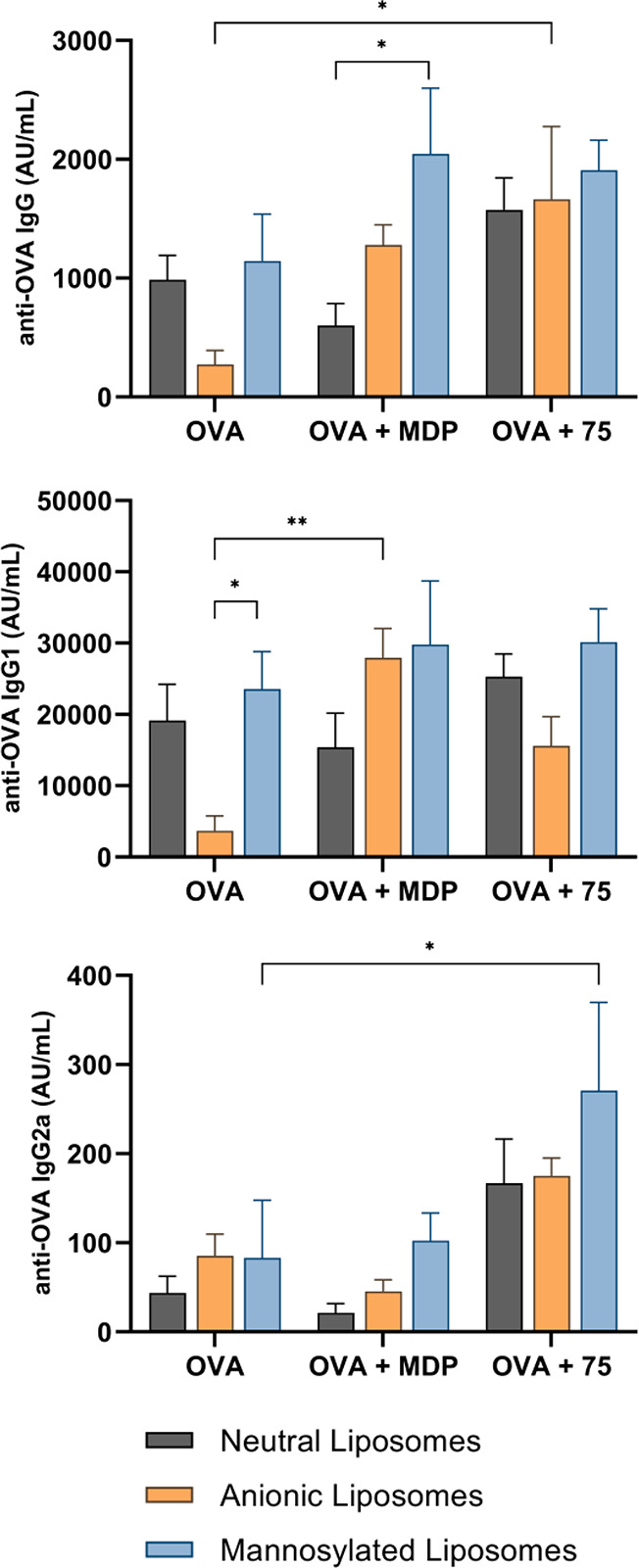
Ovalbumin-specific IgG (top), IgG1 (middle), and IgG2a (bottom) responses in NIH/OlaHsd mice after immunization with neutral (phosphatidylcholine–cholesterol, 7:5), anionic (phosphatidylcholine–cholesterol–dicetylphosphate, 7:5:1), and mannosylated (phosphatidylcholine–cholesterol–mannose-PEG-PA, 7:5:0.5) ovalbumin-loaded liposomes (10 μg of ovalbumin per dose), with adjuvants MDP or **75** (0.15 μmol of adjuvant per dose). The concentrations were measured 1 week after the second booster dose. Data are expressed as mean ± SEM of 5 mice per group. *p < 0.05, **p < 0.01.

The measured ovalbumin-specific IgG, IgG1, and IgG2a antibody levels after immunization with MDP or **75** adjuvants in neutral, negatively charged, and mannosylated liposomes (Figure 11) led to several observations: (i) Contrary to previous reports,^124^ negatively charged liposomes without adjuvant elicited a substantially weaker ovalbumin-specific humoral response compared to both neutral and mannosylated liposomes, both of which exhibited similar adjuvant activities. It is worth noting that the entrapment efficiency of ovalbumin was not evaluated in our study and might contribute to the reduced adjuvanticity of negatively charged liposomes. (ii) The composition of liposomes with MDP-adjuvant significantly influenced the intensity of the immune response. Neutral liposomes had the weakest adjuvant activities, and interestingly, this was even weaker than the neutral liposomes without adjuvant. A stronger IgG response was observed with negatively charged liposomes, while mannosylated liposomes showed the strongest immunogenicity. The levels of Th2-associated IgG1 antibodies followed the same trend. The Th1-associated IgG2a response, however, remained unchanged, compared to liposomes without adjuvant, which is consistent with the predominantly Th2-biased adjuvant activity of MDP.^15^ (iii) The liposomal composition had little influence on the intensity of the **75**-elicited IgG response, although it did alter the Th1/Th2 bias of the provoked immune response. Namely, the negatively charged liposomes showed diminished IgG1 activity. Mannosylated liposomes, on the other hand, enhanced the production of IgG2a antibodies, which is in agreement with previous reports of Th1-biased immune responses upon immunization with mannosylated liposomes with NOD2 agonist adjuvant.^125^ (iv) Finally, regardless of the liposomal composition, **75** increased the levels of IgG antibodies compared to the controls without adjuvant, with comparable or stronger adjuvant activities than MDP. Additionally, while MDP elicited a predominantly Th2-biased IgG1 response, **75** also augmented the production of ovalbumin-specific IgG2a antibodies, resulting in a mixed Th1/Th2 response.

The combined data from both of the *in vivo* experiments showed that introduction of a C_18_ lipophilic anchor into the structure of **1**, to produce **75**, substantially improved the **1***in vivo* adjuvant activity, which appears to be due to the stronger incorporation and retention of **75** in the liposomal lipid bilayer. Similar transformations were applied previously to MDP and its derivatives, which resulted in higher immunoadjuvant activities and, importantly, reduced pyrogenicity, one of the main factors that has hindered the use of MDP in the clinic.^70^ In line with previous reports, the increased lipophilicity additionally augmented the production of IgG2a antibodies, which indicated a shift toward a balanced Th1/Th2 immune response, especially when **75** was administered in the form of mannosylated liposomes. Given that the majority of the currently used adjuvants induce predominantly Th2-biased responses, the eliciting of a Th1-biased or balanced response remains a highly sought after trait for adjuvants; for example, induction of Th1 responses is highly desirable for vaccines that target intracellular pathogens or cancers. Furthermore, when another representative MDP derivative was used in an intranasal mucosal vaccine (i.e., murabutide), this induced robust systemic and mucosal immunity that was characterized by IgG and IgA levels higher than those after parenteral vaccination with alum-adjuvant.^22^ We thus postulate that **75** shows similar potential, and propose **75**-adjuvanted mucosal vaccination as the next potential direction for further research.

## 3 Conclusions

In the present study, we performed a focused structure–activity relationship optimization of **1**, which resulted in a library of novel desmuramylpeptide NOD2 agonists. In particular, we have identified two promising compounds: **68**, as a potent *in vitro* NOD2 agonist with a more than 2-fold improved potency over **1**, and **75**, which shows superior adjuvant activity *in vivo*. Both **68** and **75** induced cytokine production in PBMCs, both alone and in combination with LPS, and enhanced antigen presentation of DCs. Furthermore, **75** stimulated the cytotoxic activity of PBMCs against malignant cells. Interestingly, the *in vitro* NOD2 activation and *in vivo* adjuvant activities do not necessarily correlate in a linear fashion and are highly dependent on the lipophilicity of the compounds. Specifically, we identified a C_18_ lipophilic chain as a pivotal moiety, which conferred *in vivo* adjuvant activity if administered with a liposomal delivery system. As a C_18_-lipidated derivative of **1**, **75** showed promising adjuvant activity *in vivo* in a mouse model of adjuvanticity, surpassing that of MDP and achieving a more balanced Th1/Th2 immune response. The **75***bona fide* induction of an immune response to a model antigen in mice thus highlights its potential as a vaccine adjuvant. Taken together, these data provide deeper understanding of the desmuramylpeptide structural features required to achieve *in vitro* and *in vivo* immunostimulatory activities.

## 4 Experimental Section

### 4.1 Materials

Chemicals were obtained from Sigma-Aldrich (St. Louis, MO, U.S.A.), Tokyo Chemical Industry (Tokyo, Japan), Acros Organics (Geel, Belgium), Enamine (Monmouth Junction, NJ, U.S.A.), and Apollo (Stockport, U.K.) and were used without further purification. MDP, C12-iE-DAP, and LPS (from *E. coli* O55:B5) were obtained from InvivoGen, Inc. (San Diego, CA, U.S.A.). Analytical TLC was performed on Merck 60 F254 silica gel plates (0.25 mm), with visualization using ultraviolet light, ninhydrin, and potassium permanganate. Column chromatography was carried out on silica gel 60 (particle size 240–400 mesh). ^1^H and ^13^C NMR spectra were recorded at 400 and 100 MHz, respectively, on an Avance III spectrometer (Bruker Corporation, Billerica, MA, U.S.A.) in CDCl_3_, DMSO-*d*_6,_ or deuterated methanol (MeOD) with tetramethylsilane as the internal standard. NOESY spectra were recorded at 800 MHz on an Agilent Technologies NMR spectrometer (Santa Clara, CA, U.S.A.). Mass spectra were obtained using an Exactive Plus orbitrap mass spectrometer (Thermo Fisher Scientific, Waltham, MA, U.S.A.) or on Expression CMS mass spectrometer (Advion Inc., Ithaca, NY, U.S.A.). Analytical UHPLC analyses were performed on a Dionex UltiMate 3000 Rapid Separation Binary System (Thermo Fisher Scientific, Waltham, MA, U.S.A.) equipped with an autosampler, a binary pump system, a photodiode array detector, a thermostatted column compartment, and the Chromeleon Chromatography data system. The columns used were Waters Acquity UPLC BEH C18 (1.7 μm, 2.1 mm × 50 mm) or Waters Acquity UPLC CSH C18 (1.7 μm, 2.1 mm × 50 mm) with a flow rate of 0.3 mL/min. The eluent was a mixture of 0.1% TFA in water (A) and acetonitrile (B) with a gradient (%B) as follows: 0–10 min, 5–95%; 10–12 min, 95%; 12–12.5 min, 95–5%. The columns were thermostatted at 40 °C. All of the compounds tested were established to be ≥95% pure.

Compounds **1**, **2**, **3**, **6**, **29**, **42**, **43**, **44**, **45**, and **46** were prepared as previously described by our group.^46^ The NOD2 antagonist was synthesized as described.^77^ The analytical data here were identical to those reported previously. The assembly of the final compounds was as described below, while the preparation of other precursors is given in the Supporting Information.

### 4.2 General Synthetic Procedures

#### 4.2.1 General Procedure A: TFA-Mediated Acidolysis

The Boc-protected compound was added to an ice-chilled stirred mixture of TFA and DCM (1:5), and the mixture was allowed to warm to room temperature. After 3 h, the solvent was evaporated *in vacuo*. The residue was washed three times with diethyl ether.

#### 4.2.2 General Procedure B: EDC-Mediated Coupling

To an ice-chilled stirred solution of the requisite amine (1 equiv) and carboxylic acid (1.0–1.2 equiv) in dry dimethylformamide (DMF), *N*,*N*-diisopropylethylamine (DIPEA; 3 equiv) was added. After stirring for 15 min, HOBt (1.0–1.2 equiv), EDC (1.0–1.2 equiv), and a catalytic amount of 4-dimethylaminopyridine (DMAP) were added, and the mixture was allowed to warm to room temperature. The stirring was continued overnight, after which the mixture was diluted with ethyl acetate (EtOAc; 40 mL) and washed with 1 M HCl (2 × 20 mL), saturated NaHCO_3_ solution (2 × 20 mL), and brine (20 mL). The organic layer was dried over anhydrous Na_2_SO_4_ and concentrated *in vacuo*. If necessary, the residues were further purified using column chromatography to produce sufficiently pure compounds.

#### 4.2.3 General Procedure C: HCl-Mediated Acidolysis

To an ice-chilled stirring solution of the Boc-protected compound in EtOAc (3 mL), 1 M HCl solution in acetic acid (2 mL) was added dropwise. The mixture was allowed to warm to room temperature. The stirring was continued for 2 h, after which the mixture was diluted with EtOAc (30 mL) and washed with a 1 M NaOH solution (3 × 10 mL). The organic layer was dried over anhydrous Na_2_SO_4_ and concentrated *in vacuo* to produce the deprotected amine.

#### 4.2.4 General Procedure D: Acylation with Acyl Chlorides

To an ice-chilled stirring solution of the alcohol or amine (1 equiv) in THF, Et_3_N (1.2 equiv) and the requisite acyl chloride (1.2 equiv) were added dropwise, and the resulting mixture was allowed to warm to room temperature. The stirring was continued for 1 h, after which the mixture was diluted with EtOAc (25 mL) and washed with 1 M HCl (2 × 10 mL), saturated NaHCO_3_ solution (2 × 10 mL), and brine (10 mL). The organic layer was dried over anhydrous Na_2_SO_4_ and concentrated *in vacuo*. If necessary, the residues were further purified using column chromatography, to produce sufficiently pure compounds.

### 4.3 Synthesis and Characterization of Compounds

#### 4.3.1 Diethyl (3-(4-Hydroxy-3-methoxyphenyl)propanoyl)glycyl-l-valyl-d-glutamate (**18**)

A solution of compound **1** (64 mg, 0.120 mmol) in acetic acid (10 mL) was hydrogenated over 10% palladium-on-carbon for 2 h at room temperature and under atmospheric pressure. The catalyst was removed by filtration, and the filtrate was concentrated *in vacuo* to produce the title compound **18** as a white solid (65 mg, 100%). ^1^H NMR (400 MHz, DMSO-*d*_6_) δ 8.71 (br, 1H), 8.38 (d, *J* = 7.6 Hz, 1H), 8.19–8.10 (m, 1H), 7.84 (d, *J* = 8.9 Hz, 1H), 6.76 (d, *J* = 2.0 Hz, 1H), 6.65 (d, *J* = 7.9 Hz, 1H), 6.57 (dd, *J* = 8.1, 2.0 Hz, 1H), 4.30–4.19 (m, 2H), 4.12–3.99 (m, 4H), 3.90 (d, *J* = 4.8 Hz, 1H), 3.78–3.70 (m, 4H), 2.74–2.64 (m, 2H), 2.43–2.31 (m, 4H), 2.06–1.92 (m, 2H), 1.90–1.76 (m, 1H), 1.22–1.12 (m, 6H), 0.90–0.76 (m, 6H). ^13^C NMR (100 MHz, DMSO-*d*_6_) δ 172.57, 172.04, 171.54, 171.42, 169.50, 147.82, 145.03, 132.54, 120.63, 115.70, 112.76, 61.07, 60.42, 57.78, 55.90, 51.52, 42.49, 37.70, 31.23, 31.14, 30.17, 26.35, 19.56, 18.17, 14.52, 14.45. HRMS calcd for C_26_H_40_N_3_O_9_*m*/*z*: 538.2759 (M + H)^+^, found 538.2756.

#### 4.3.2 Diethyl (4-(4-Hydroxy-3-methoxyphenyl)butanoyl)glycyl-l-valyl-d-glutamate (**19**)

Compound **6** (83 mg, 0.179 mmol) was deprotected using general procedure A and coupled to **16** (42 mg, 0.200 mmol) using general procedure B. The residue was washed twice with diethyl ether to produce the title compound **19** as an off-white solid (46 mg, 46%). ^1^H NMR (400 MHz, DMSO-*d*_6_) δ 8.63 (s, 1H), 8.35 (d, *J* = 7.6 Hz, 1H), 8.07 (t, *J* = 5.9 Hz, 1H), 7.73 (d, *J* = 9.0 Hz, 1H), 6.73 (s, 1H), 6.65 (d, *J* = 7.9 Hz, 1H), 6.55 (d, *J* = 7.8 Hz, 1H), 4.27–4.21 (m, 2H), 4.11–4.01 (m, 4H), 3.77–3.69 (m, 5H), 2.45 (t, *J* = 7.8 Hz, 2H), 2.34 (t, *J* = 7.6 Hz, 2H), 2.12 (t, *J* = 7.5 Hz, 2H), 2.02–1.93 (m, 2H), 1.86–1.79 (m, 1H), 1.75 (p, *J* = 7.5 Hz, 2H), 1.19–1.14 (m, 6H), 0.85–0.80 (m, 6H). ^13^C NMR (100 MHz, CDCl_3_) δ 173.92, 172.86, 171.68, 171.35, 169.60, 146.54, 143.80, 133.38, 120.96, 114.30, 111.15, 61.64, 60.80, 58.44, 55.86, 51.86, 43.32, 35.33, 34.88, 31.01, 30.37, 27.31, 26.82, 19.26, 17.83, 14.14, 14.08. HRMS calcd for C_27_H_42_N_3_O_9_*m*/*z*: 552.2916 (M + H)^+^, found 552.2907.

#### 4.3.3 Diethyl ((*E*)-3-(3,4-Dimethoxyphenyl)acryloyl)glycyl-l-valyl-d-glutamate (**20**)

Compound **6** (138 mg, 0.300 mmol) was deprotected using general procedure A and coupled to *trans*-3,4-dimethoxycinnamic acid (68 mg, 0.330 mmol) using general procedure B. The residue was washed twice with diethyl ether to produce the title compound **20** as an off-white solid (102 mg, 64%). ^1^H NMR (400 MHz, MeOD) δ 7.51 (d, *J* = 15.6 Hz, 1H), 7.22–7.13 (m, 2H), 6.99 (d, *J* = 8.4 Hz, 1H), 6.57 (d, *J* = 15.7 Hz, 1H), 4.49–4.41 (m, 1H), 4.34–4.28 (m, 1H), 4.26–4.04 (m, 6H), 3.89 (s, 3H), 3.88 (s, 3H), 2.40 (t, *J* = 7.1 Hz, 2H), 2.27–2.13 (m, 2H), 2.09–1.97 (m, 1H), 1.32–1.18 (m, 6H), 1.00 (t, *J* = 6.9 Hz, 6H). ^13^C NMR (100 MHz, MeOD) δ 172.94, 172.35, 171.55, 170.61, 168.07, 150.97, 149.31, 141.05, 127.82, 121.99, 117.56, 111.25, 110.02, 61.06, 60.28, 58.62, 55.02, 51.79, 42.58, 30.43, 29.85, 25.86, 18.35, 16.65, 13.07. HRMS calcd for C_27_H_40_N_3_O_9_*m*/*z*: 550.2759 (M + H)^+^, found 550.2756.

#### 4.3.4 Diethyl ((*E*)-3-(3,4-Dihydroxyphenyl)acryloyl)glycyl-l-valyl-d-glutamate (**21**)

Compound **6** (78 mg, 0.170 mmol) was deprotected using general procedure A and coupled to *trans*-caffeic acid (34 mg, 0.187 mmol) using general procedure B. The residue was washed twice with diethyl ether to produce the title compound **21** as a cream-colored solid (43 mg, 49%). ^1^H NMR (400 MHz, MeOD) δ 7.44 (d, *J* = 15.7 Hz, 1H), 7.04 (d, *J* = 2.1 Hz, 1H), 6.94 (dd, *J* = 8.2, 2.1 Hz, 1H), 6.78 (d, *J* = 8.2 Hz, 1H), 6.45 (d, *J* = 15.7 Hz, 1H), 4.49–4.41 (m, 1H), 4.31 (d, *J* = 6.1 Hz, 1H), 4.24–4.15 (m, 2H), 4.15–3.93 (m, 4H), 2.43 (t, *J* = 7.2 Hz, 2H), 2.30–2.11 (m, 2H), 2.08–1.93 (m, 1H), 1.32–1.15 (m, 6H), 1.00 (t, *J* = 7.0 Hz, 6H). ^13^C NMR (100 MHz, MeOD) δ 172.95, 172.36, 171.54, 170.69, 168.37, 147.57, 145.36, 141.60, 126.75, 120.93, 116.29, 115.04, 113.69, 61.07, 60.30, 58.63, 51.78, 42.58, 30.43, 29.85, 25.85, 18.34, 16.65, 13.06. HRMS calcd for C_25_H_36_N_3_O_9_*m*/*z*: 522.2446 (M + H)^+^, found 522.2445.

#### 4.3.5 Diethyl ((*E*)-3-(4-Aminophenyl)acryloyl)glycyl-l-valyl-d-glutamate (**24**)

Compound **6** (276 mg, 0.600 mmol) was deprotected using general procedure A and coupled to **22** (174 mg, 0.660 mmol) using general procedure B. The residue was washed twice with diethyl ether to produce compound **23** as a yellow solid (179 mg, 49%). This compound was deprotected using general procedure A. The residue was dissolved in EtOAc (50 mL) and washed with a saturated NaHCO_3_ solution (3 × 30 mL) and brine (30 mL). The organic layer was dried over anhydrous Na_2_SO_4_ and concentrated *in vacuo* to produce the title compound **24** as an orange solid (135 mg, 91%). ^1^H NMR (400 MHz, MeOD) δ 7.46 (d, *J* = 15.7 Hz, 1H), 7.34 (d, *J* = 8.6 Hz, 2H), 6.70 (d, *J* = 8.6 Hz, 2H), 6.42 (d, *J* = 15.7 Hz, 1H), 4.48–4.42 (m, 1H), 4.31 (d, *J* = 6.0 Hz, 1H), 4.23–4.14 (m, 2H), 4.14–3.92 (m, 4H), 2.43 (t, *J* = 7.3 Hz, 2H), 2.27–2.13 (m, 2H), 2.08–1.95 (m, 1H), 1.28 (t, *J* = 7.2 Hz, 3H), 1.22 (t, *J* = 7.2 Hz, 3H), 0.99 (t, *J* = 7.2 Hz, 6H). ^13^C NMR (100 MHz, MeOD) δ 172.95, 172.34, 171.54, 170.77, 168.77, 150.25, 141.92, 129.24, 123.70, 114.39, 114.16, 61.06, 60.29, 58.62, 51.80, 42.63, 30.43, 29.86, 25.87, 18.34, 16.65, 13.06. HRMS calcd for C_25_H_37_N_4_O_7_*m*/*z*: 505.2657 (M + H)^+^, found 505.2641.

#### 4.3.6 Diethyl ((*E*)-3-(4-Isopropylphenyl)acryloyl)glycyl-l-valyl-d-glutamate (**25**)

Compound **6** (92 mg, 0.200 mmol) was deprotected using general procedure A and coupled to *trans-*4-isopropylcinnamic acid (42 mg, 0.220 mmol) using general procedure B. The residue was washed twice with diethyl ether to produce the title compound **25** as a white solid (44 mg, 42%). ^1^H NMR (400 MHz, DMSO-*d*_6_) δ 8.42–8.31 (m, 2H), 7.94 (d, *J* = 9.0 Hz, 1H), 7.49 (d, *J* = 7.9 Hz, 2H), 7.40 (d, *J* = 15.7 Hz, 1H), 7.29 (d, *J* = 7.9 Hz, 2H), 6.69 (d, *J* = 15.8 Hz, 1H), 4.31–4.21 (m, 2H), 4.14–3.97 (m, 4H), 3.90 (d, *J* = 5.8 Hz, 2H), 2.96–2.84 (m, 1H), 2.35 (t, *J* = 7.5 Hz, 2H), 2.05–1.93 (m, 2H), 1.89–1.76 (m, 1H), 1.25–1.10 (m, 12H), 0.85 (t, *J* = 6.5 Hz, 6H). ^13^C NMR (100 MHz, DMSO-*d*_6_) δ 171.95, 171.44, 170.92, 168.82, 165.34, 149.99, 138.84, 132.40, 127.54, 126.79, 120.75, 60.48, 59.81, 57.27, 50.97, 42.10, 33.20, 30.66, 29.60, 25.81, 23.57, 19.02, 17.64, 13.95, 13.90. HRMS calcd for C_28_H_42_N_3_O_7_*m*/*z*: 532.3017 (M + H)^+^, found 532.3012.

#### 4.3.7 Diethyl ((*E*)-3-(4-Nitrophenyl)acryloyl)glycyl-l-valyl-d-glutamate (**26**)

Compound **6** (92 mg, 0.200 mmol) was deprotected using general procedure A and coupled to *trans-*4-nitrocinnamic acid (42 mg, 0.220 mmol) using general procedure B. The residue was washed twice with diethyl ether to produce the title compound **26** as a yellow solid (69 mg, 65%). ^1^H NMR (400 MHz, DMSO-*d*_6_) δ 8.51 (t, *J* = 5.8 Hz, 1H), 8.40 (d, *J* = 7.5 Hz, 1H), 8.27 (d, *J* = 8.7 Hz, 2H), 8.00 (d, *J* = 9.0 Hz, 1H), 7.85 (d, *J* = 8.8 Hz, 2H), 7.55 (d, *J* = 15.9 Hz, 1H), 6.95 (d, *J* = 15.9 Hz, 1H), 4.31–4.21 (m, 2H), 4.15–3.97 (m, 4H), 3.94 (d, *J* = 5.7 Hz, 2H), 2.35 (t, *J* = 7.5 Hz, 2H), 2.07–1.93 (m, 2H), 1.91–1.76 (m, 1H), 1.22–1.11 (m, 6H), 0.85 (t, *J* = 6.3 Hz, 6H). ^13^C NMR (100 MHz, DMSO-*d*_6_) δ 172.53, 172.01, 171.47, 169.12, 165.04, 148.00, 141.94, 137.14, 129.09, 126.59, 124.59, 61.05, 60.39, 57.87, 51.53, 42.69, 31.26, 30.18, 26.40, 19.58, 18.25, 14.52, 14.47. HRMS calcd for C_25_H_35_N_4_O_9_*m*/*z*: 535.2399 (M + H)^+^, found 535.2393.

#### 4.3.8 Diethyl (2-(3,4-Dimethoxyphenyl)cyclopropane-1-carbonyl)glycyl-l-valyl-d-glutamate (**27**)

Compound **6** (92 mg, 0.200 mmol) was deprotected using general procedure A and coupled to **13** (49 mg, 0.220 mmol) using general procedure B. Purification by flash chromatography (5% MeOH in DCM) produced the title compound **27** as a yellow solid (36 mg, 31%). ^1^H NMR (400 MHz, DMSO-*d*_6_) δ 8.43–8.36 (m, 2H), 7.85–7.77 (m, 1H), 6.84 (d, *J* = 8.3 Hz, 1H), 6.75–6.69 (m, 1H), 6.62 (dd, *J* = 8.2, 2.1 Hz, 1H), 4.29–4.20 (m, 2H), 4.14–3.98 (m, 4H), 3.84–3.76 (m, 2H), 3.73 (s, 3H), 3.70 (s, 3H), 2.35 (t, *J* = 7.6 Hz, 2H), 2.22–2.14 (m, 1H), 2.06–1.88 (m, 3H), 1.88–1.77 (m, 1H), 1.32–1.24 (m, 1H), 1.21–1.11 (m, 7H), 0.89–0.79 (m, 6H). ^13^C NMR (100 MHz, MeOD) δ 174.23, 172.92, 172.27, 171.56, 170.55, 149.07, 147.75, 133.45, 117.96, 111.70, 110.29, 61.06, 60.31, 58.40, 55.13, 55.05, 51.78, 42.65, 30.57, 29.84, 25.88, 24.96, 24.58, 18.35, 16.64, 14.62, 13.10, 13.06. HRMS calcd for C_28_H_40_N_3_O_9_*m*/*z*: 562.2770 (M – H)^−^, found 562.2769.

#### 4.3.9 Diethyl (2-(4-Hydroxy-3-methoxyphenyl)cyclopropane-1-carbonyl)glycyl-l-valyl-d-glutamate (**28**)

Compound **6** (111 mg, 0.242 mmol) was deprotected using general procedure A and coupled to **17** (42 mg, 0.202 mmol) using general procedure B. White solid (98 mg, 88%). ^1^H NMR (400 MHz, DMSO-*d*_6_) δ 8.75 (s, 1H), 8.41–8.35 (m, 2H), 7.84–7.76 (m, 1H), 6.70–6.67 (m, 1H), 6.66 (d, *J* = 8.1 Hz, 1H), 6.49 (dd, *J* = 8.1, 2.0 Hz, 1H), 4.31–4.20 (m, 2H), 4.14–3.99 (m, 4H), 3.83–3.77 (m, 2H), 3.74 (s, 3H), 2.35 (t, *J* = 7.5 Hz, 2H), 2.19–2.09 (m, 1H), 2.03–1.93 (m, 2H), 1.92–1.86 (m, 1H), 1.85–1.77 (m, 1H), 1.31–1.22 (m, 1H), 1.22–1.12 (m, 7H), 0.83 (t, *J* = 7.5 Hz, 6H). ^13^C NMR (100 MHz, DMSO-*d*_6_) δ 172.52, 172.17, 172.14, 172.00, 171.48, 169.41, 147.94, 145.27, 132.10, 118.35, 115.81, 110.99, 61.05, 60.41, 57.65, 57.62, 56.03, 51.52, 42.68, 42.63, 31.34, 31.31, 30.17, 26.41, 25.50, 24.45, 19.56, 18.14, 15.38, 14.53, 14.46. HRMS calcd for C_27_H_40_N_3_O_9_*m*/*z*: 550.2759 (M + H)^+^, found 550.2748.

#### 4.3.10 Diethyl ((*E*)-3-(4-Hydroxy-3-methoxyphenyl)acryloyl)glycyl-l-threonyl-d-glutamate (**32**)

Compound **9** (388 mg, 0.841 mmol) was deprotected using general procedure A and coupled to *trans*-ferulic acid (180 mg, 0.925 mmol) using general procedure B. Purification by flash chromatography (6% MeOH in DCM) produced the title compound **32** as a yellow solid (263 mg, 58%). ^1^H NMR (400 MHz, CDCl_3_) δ 7.74 (d, *J* = 7.6 Hz, 1H), 7.57 (d, *J* = 8.1 Hz, 1H), 7.51 (d, *J* = 15.5 Hz, 1H), 7.14 (t, *J* = 5.3 Hz, 1H), 7.04–6.95 (m, 2H), 6.85 (d, *J* = 8.0 Hz, 1H), 6.43–6.31 (m, 2H), 4.58–4.47 (m, 2H), 4.45–4.35 (m, 1H), 4.23–4.01 (m, 6H), 3.86 (s, 3H), 3.79 (d, *J* = 6.4 Hz, 1H), 2.43 (t, *J* = 7.4 Hz, 2H), 2.29–2.14 (m, 1H), 2.13–1.97 (m, 1H), 1.28–1.14 (m, 9H). ^13^C NMR (100 MHz, CDCl_3_) δ 173.08, 172.23, 171.25, 170.31, 167.36, 147.69, 146.86, 142.03, 127.09, 122.31, 117.15, 114.84, 110.06, 67.30, 61.91, 60.84, 58.35, 55.92, 52.35, 43.70, 30.54, 26.26, 18.82, 14.12, 14.07. HRMS calcd for C_25_H_36_N_3_O_10_*m*/*z*: 538.2395 (M + H)^+^, found 538.2396.

#### 4.3.11 Diethyl *O*-Benzyl-*N*-(((*E*)-3-(4-hydroxy-3-methoxyphenyl)acryloyl)glycyl)-l-seryl-d-glutamate (**30**)

Compound **7** (227 mg, 0.420 mmol) was deprotected using general procedure A and coupled to *trans*-ferulic acid (82 mg, 0.420 mmol) using general procedure B. Yellow solid (177 mg, 69%). ^1^H NMR (400 MHz, CDCl_3_) δ 7.71 (d, *J* = 7.9 Hz, 1H), 7.60 (d, *J* = 7.9 Hz, 1H), 7.52 (d, *J* = 15.6 Hz, 1H), 7.33–7.17 (m, 6H), 7.02–6.94 (m, 2H), 6.84 (d, *J* = 8.0 Hz, 1H), 6.36 (d, *J* = 15.5 Hz, 1H), 4.85–4.71 (m, 1H), 4.62–4.43 (m, 3H), 4.21–3.97 (m, 6H), 3.91–3.83 (m, 1H), 3.81 (s, 3H), 3.69–3.57 (m, 1H), 2.35 (t, *J* = 7.7 Hz, 2H), 2.26–2.11 (m, 1H), 2.07–1.94 (m, 1H), 1.26–1.11 (m, 6H). ^13^C NMR (100 MHz, CDCl_3_) δ 172.85, 171.70, 170.05, 169.86, 167.25, 147.78, 147.04, 141.84, 137.40, 128.43, 127.81, 127.73, 127.11, 122.20, 117.30, 115.01, 110.29, 73.40, 73.30, 69.54, 61.59, 60.64, 55.88, 53.03, 51.99, 43.60, 30.22, 26.93, 26.88. HRMS calcd for C_31_H_40_N_3_O_10_*m*/*z*: 614.2708 (M + H)^+^, found 614.2695.

#### 4.3.12 Diethyl ((*E*)-3-(4-Hydroxy-3-methoxyphenyl)acryloyl)glycyl-l-seryl-d-glutamate (**31**)

Compound **8** (277 mg, 0.620 mmol) was deprotected using general procedure A and coupled to *trans*-ferulic acid (120 mg, 0.620 mmol) using general procedure B. Orange solid (150 mg, 46%). ^1^H NMR (400 MHz, MeOD) δ 7.52 (d, *J* = 15.7 Hz, 1H), 7.18 (d, *J* = 1.9 Hz, 1H), 7.08 (d, *J* = 8.5 Hz, 1H), 6.83 (d, *J* = 8.2 Hz, 1H), 6.54 (d, *J* = 15.7 Hz, 1H), 4.54–4.45 (m, 2H), 4.25–4.01 (m, 6H), 3.92 (s, 3H), 3.89–3.79 (m, 2H), 2.49–2.40 (m, 2H), 2.29–2.17 (m, 1H), 2.10–2.03 (m, 1H), 1.35–1.18 (m, 6H). ^13^C NMR (101 MHz, MeOD) δ 173.06, 171.69, 171.02, 170.72, 168.48, 148.68, 147.91, 141.59, 126.73, 122.00, 116.56, 115.08, 110.33, 61.46, 61.15, 60.26, 55.44, 55.00, 51.93, 42.91, 29.82, 29.37, 26.01, 13.04. HRMS calcd for C_24_H_34_N_3_O_10_*m*/*z*: 524.2239 (M + H)^+^, found 524.2231.

#### 4.3.13 Methyl ((*R*)-5-Ethoxy-4-((*S*)-2-(2-((*E*)-3-(4-hydroxy-3-methoxyphenyl)acrylamido)acetamido)-3-methylbutanamido)-5-oxopentanoyl)-l-lysinate (**41**)

To a stirring solution of compound **40** (104 mg, 0.119 mmol) in THF (15 mL), 1-octanethiol (210 μL, 1.19 mmol) and 1,8-diazabicyclo[5.4.0]undec-7-ene (DBU; 9 μL, 0.060 mmol) were added. The resulting mixture was stirred overnight at room temperature. The solvent was evaporated *in vacuo*, and the residue was washed twice with diethyl ether then recrystallized from ethanol/diethyl ether to produce the title compound **41** as a yellow solid (40 mg, 52%). ^1^H NMR (400 MHz, MeOD) δ 7.47 (d, *J* = 15.7 Hz, 1H), 7.13 (d, *J* = 2.0 Hz, 1H), 7.05 (dd, *J* = 8.2, 2.0 Hz, 1H), 6.77 (d, *J* = 8.2 Hz, 1H), 6.48 (d, *J* = 15.6 Hz, 1H), 4.43–4.35 (m, 1H), 4.33–4.07 (m, 4H), 3.90 (s, 3H), 3.71 (s, 3H), 3.66–3.59 (m, 1H), 3.61–3.51 (m, 1H), 2.62 (t, *J* = 7.1 Hz, 2H), 2.40–2.18 (m, 4H), 2.11–2.01 (m, 2H), 1.84–1.70 (m, 2H), 1.67–1.50 (m, 1H), 1.49–1.38 (m, 2H), 1.29 (t, *J* = 7.1 Hz, 3H), 1.02 (t, *J* = 6.6 Hz, 6H). ^13^C NMR (100 MHz, DMSO-*d*_6_) δ 173.24, 172.22, 171.97, 171.59, 169.51, 166.35, 165.79, 148.35, 140.00, 126.67, 122.12, 118.80, 116.16, 111.37, 60.93, 55.96, 53.76, 52.43, 52.28, 52.19, 48.32, 42.76, 38.08, 32.00, 28.74, 26.45, 23.88, 19.65, 19.41, 18.26, 14.50. HRMS calcd for C_31_H_48_N_5_O_10_*m*/*z*: 650.3396 (M + H)^+^, found 650.3387.

#### 4.3.14 Di-*tert*-butyl ((*E*)-3-(4-Hydroxy-3-methoxyphenyl)acryloyl)glycyl-l-valyl-d-glutamate (**56**)

Compound **47** (55 mg, 0.153 mmol) was deprotected using general procedure C and coupled to **46** (64 mg, 0.184 mmol) using general procedure B. Purification by flash chromatography (5% MeOH in DCM) produced the title compound **56** as an orange solid (27 mg, 30%). ^1^H NMR (400 MHz, MeOD) δ 7.44 (d, *J* = 15.6, 1H), 7.11 (d, *J* = 1.6 Hz, 1H), 7.01 (dd, *J* = 8.4 Hz, *J* = 2.0 Hz, 1H), 6.76 (d, *J* = 8.0 Hz, 1H), 6.46 (d, *J* = 16.0 Hz, 1H), 4.29–4.25 (m, 2H), 4.02–3.91 (m, 2H), 3.85 (s, 3H), 2.26 (t, *J* = 7.6 Hz, 2H), 2.15–2.00 (m, 2H), 1.94–1.81 (m, 1H), 1.42 (s, 9H), 1.38 (s, 9H), 0.93 (t, *J* = 7.2 Hz, 6H). ^13^C NMR (100 MHz, CDCl_3_) δ 173.12, 172.28, 171.23, 170.71, 166.75, 165.00, 147.17, 146.93, 144.83, 144.20, 138.75, 127.17, 122.49, 117.24, 114.70, 110.20, 80.92, 58.47, 52.43, 38.61, 28.10, 28.01, 27.96, 27.32. HRMS calcd for C_30_H_44_N_3_O_9_*m*/*z*: 590.3078 (M – H)^−^, found 590.3062.

#### 4.3.15 (*R*)-*N*^1^,*N*^5^-Diethyl-2-((*S*)-2-(2-((*E*)-3-(4-hydroxy-3-methoxyphenyl)acrylamido)acetamido)-3-methylbutanamido)pentanediamide (**57**)

Compound **48** (108 mg, 0.326 mmol) was deprotected using general procedure C and coupled to **46** (139 mg, 0.391 mmol) using general procedure B to produce the title compound **57** as an orange solid (99 mg, 57%). ^1^H NMR (400 MHz, DMSO-*d*_6_) δ 9.45 (s, 1H), 8.40–8.13 (m, 2H), 8.03–8.00 (m, 1H), 7.90–7.75 (m, 2H), 7.33 (d, *J* = 16.0, 1H), 7.14 (d, *J* = 1.6 Hz, 1H), 7.00 (dd, *J* = 8.0 Hz, *J* = 1.6 Hz, 1H), 6.79 (d, *J* = 8.0 Hz, 1H), 6.57 (d, *J* = 15.6 Hz, 1H), 4.28–4.05 (m, 2H), 3.92–3.82 (m, 2H), 3.80 (s, 3H), 3.07–3.02 (m, 4H), 2.18–1.86 (m, 3H), 1.83–1.60 (m, 2H), 1.19–1.00 (m, 6H), 0.98–0.96 (m, 6H). ^13^C NMR (100 MHz, DMSO-*d*_6_) δ 171.05, 169.51, 169.48, 169.41, 166.32, 160.85, 160.10, 159.44, 148.30, 147.77, 126.67, 125.45, 118.57, 115.61, 110.81, 107.63, 107.07, 84.11, 55.47, 46.64, 33.28, 31.40, 29.96, 27.69, 27.60, 26.69, 24.43, 21.90, 14.69. HRMS calcd for C_26_H_40_N_5_O_7_*m*/*z*: 534.2990 (M + H)^+^, found 534.2986.

#### 4.3.16 (*R*)-*N*^1^,*N*^5^-Dibutyl-2-((*S*)-2-(2-((*E*)-3-(4-hydroxy-3-methoxyphenyl)acrylamido)acetamido)-3-methylbutanamido)pentanediamide (**58**)

Compound **49** (120 mg, 0.336 mmol) was deprotected using general procedure A and coupled to **46** (130 mg, 0.370 mmol) using general procedure B to produce the title compound **58** as an orange solid (115 mg, 58%). ^1^H NMR (400 MHz, DMSO-*d*_6_) δ 9.45 (s, 1H), 8.25 (d, *J* = 8.0 Hz, 1H), 8.16 (t, *J* = 5.6 Hz, 1H), 8.05–7.94 (m, 1H), 7.85–7.71 (m, 2H), 7.33 (d, *J* = 15.6, 1H), 7.14 (d, *J* = 2.0 Hz, 1H), 7.00 (dd, *J* = 8.0 Hz, *J* = 1.2 Hz, 1H), 6.79 (d, *J* = 8.0 Hz, 1H), 6.55 (d, *J* = 16.0 Hz, 1H), 4.18–4.11 (m, 2H), 3.92–3.86 (m, 2H), 3.80 (s, 3H), 3.06–3.00 (m, 4H), 2.10–1.99 (m, 2H), 1.98–1.81 (m, 2H), 1.78–1.63 (m, 1H), 1.36–1.22 (m, 8H), 0.87–0.80 (m, 12H). ^13^C NMR (100 MHz, DMSO-*d*_6_) δ 171.18, 171.17, 171.13, 169.45, 147.76, 139.38, 138.21, 121.54, 115.60, 110.81, 55.46, 52.37, 38.09, 31.87, 31.21, 31.13, 19.53, 19.45, 19.08, 18.26, 13.65. HRMS calcd for C_30_H_48_N_5_O_7_*m*/*z*: 590.3554 (M + H)^+^, found 590.3542.

#### 4.3.17 *tert*-Butyl *N*^5^-Butyl-*N*^2^-((*E*)-3-(4-hydroxy-3-methoxyphenyl)acryloyl)glycyl-l-valyl-d-glutaminate (**59**)

Compound **50** (45 mg, 0.126 mmol) was deprotected using general procedure A and coupled to **46** (53 mg, 0.151 mmol) using general procedure B to produce the title compound **59** as a yellow solid (35 mg, 47%). ^1^H NMR (400 MHz, MeOD) δ 7.42 (d, *J* = 15.6 Hz, 1H), 7.10 (s, 1H), 7.00 (d, *J* = 8.0 Hz, 1H), 6.76 (d, *J* = 8.0 Hz, 1H), 6.46 (d, *J* = 15.6 Hz, 1H), 4.24–4.14 (m, 2H), 4.09–3.89 (m, 2H), 3.85 (s, 3H), 3.15–3.00 (m, 2H), 2.22–2.03 (m, 4H), 1.94–1.79 (m, 2H), 1.41 (s, 9H), 1.33–1.20 (m, 3H), 0.97–0.82 (m, 9H). ^13^C NMR (100 MHz, CDCl_3_) δ 172.46, 172.41, 171.41, 171.03, 169.98, 167.32, 167.11, 147.79, 146.92, 142.02, 141.72, 127.02, 122.29, 117.25, 114.91, 110.02, 82.22, 58.95, 55.93, 52.65, 43.81, 39.49, 32.63, 31.58, 31.52, 30.43, 27.96, 19.43, 17.64, 13.77. HRMS calcd for C_30_H_47_N_4_O_8_*m*/*z*: 591.3394 (M + H)^+^, found 591.3403.

#### 4.3.18 *tert*-Butyl *N*^2^-((*E*)-3-(4-Hydroxy-3-methoxyphenyl)acryloyl)glycyl-l-valyl-*N*^5^-methoxy-d-glutaminate (**60**)

Compound **51** (62 mg, 0.187 mmol) was deprotected using general procedure C and coupled to **46** (79 mg, 0.224 mmol) using general procedure B. Purification by flash chromatography (3% MeOH in DCM) produced the title compound **60** as an orange waxy solid (12 mg, 11%). ^1^H NMR (400 MHz, MeOD) δ 7.44 (d, *J* = 15.6 Hz, 1H), 7.11 (d, *J* = 2.0 Hz, 1H), 7.01 (dd, *J* = 8.0 Hz, *J* = 1.6 Hz, 1H), 6.76 (d, *J* = 8.0 Hz, 1H), 6.47 (d, *J* = 15.6 Hz, 1H), 4.34–4.33 (m, 1H), 4.13–4.09 (m, 1H), 4.05–3.93 (m, 2H), 3.85 (s, 3H), 3.68 (s, 3H), 2.45–2.20 (m, 2H), 2.15–2.02 (m, 2H), 1.88–1.76 (m, 1H), 1.44 (s, 9H), 0.92–0.90 (m, 6H). ^13^C NMR (100 MHz, CDCl_3_) δ 172.20, 169.08, 167.42, 166.65, 161.17, 146.74, 141.98, 127.12, 124.23, 122.54, 117.21, 114.74, 114.18, 109.51, 82.51, 57.32, 55.96, 52.30, 43.49, 31.19, 29.32, 28.29, 27.98, 24.88, 19.00, 17.75. HRMS calcd for C_27_H_39_N_4_O_9_*m*/*z*: 563.2717 (M – H)^−^, found 563.2722.

#### 4.3.19 *tert*-Butyl *N*^2^-((*E*)-3-(4-Hydroxy-3-methoxyphenyl)acryloyl)glycyl-l-valyl-*N*^5^-methoxy-*N*^5^-methyl-d-glutaminate (**61**)

Compound **52** (78 mg, 0.225 mmol) was deprotected using general procedure C and coupled to **46** (95 mg, 0.270 mmol) using general procedure B to produce the title compound **61** as an orange solid (57 mg, 44%). ^1^H NMR (400 MHz, MeOD) δ 7.44 (d, *J* = 15.6 Hz, 1H), 7.10 (d, *J* = 1.6 Hz, 1H), 7.01 (dd, *J* = 8.0 Hz, *J* = 1.6 Hz, 1H), 6.76 (d, *J* = 8.0 Hz, 1H), 6.46 (d, *J* = 15.6 Hz, 1H), 4.34–4.20 (m, 2H), 4.05–3.90 (m, 2H), 3.85 (s, 3H), 3.70–3.57 (m, 3H), 3.13–3.07 (m, 3H), 2.58–2.42 (m, 2H), 2.18–2.01 (m, 2H), 1.98–1.84 (m, 1H), 1.42–1.41 (m, 9H), 0.97–0.90 (m, 6H). ^13^C NMR (100 MHz, CDCl_3_) δ 173.63, 173.44, 171.20, 170.65, 169.65, 169.40, 166.94, 147.62, 146.88, 141.82, 141.63, 127.22, 122.25, 122.22, 117.55, 114.86, 110.01, 82.03, 65.88, 61.22, 58.43, 58.37, 55.91, 53.06, 52.99, 43.57, 43.37, 32.19, 31.26, 31.14, 28.32, 26.16, 19.35, 19.16, 18.07, 15.28. *Some signals in the ^13^C spectrum are doubled due to the presence of both l- and d-configured valine. HRMS calcd for C_28_H_41_N_4_O_9_*m*/*z*: 577.2874 (M – H)^−^, found 577.2858.

#### 4.3.20 *tert*-Butyl (*R*)-2-((*S*)-2-(2-((*E*)-3-(4-Hydroxy-3-methoxyphenyl)acrylamido)acetamido)-3-methylbutanamido)-5-oxo-5-(pyrrolidin-1-yl)pentanoate (**62**)

Compound **53** (70 mg, 0.196 mmol) was deprotected using general procedure C and coupled to **46** (82 mg, 0.235 mmol) using general procedure B to produce the title compound **62** as a yellow solid (65 mg, 57%). ^1^H NMR (400 MHz, MeOD) δ 7.44 (d, *J* = 16.0 Hz, 1H), 7.10 (s, 1H), 7.00 (d, *J* = 8.0 Hz, 1H), 6.76 (d, *J* = 8.0 Hz, 1H), 6.46 (d, *J* = 15.6 Hz, 1H), 4.42–4.16 (m, 2H), 4.09–3.90 (m, 2H), 3.84 (s, 3H), 3.83–3.65 (m, 2H), 3.43–3.31 (m, 2H), 2.43–2.25 (m, 2H), 2.21–1.68 (m, 7H), 1.44–1.37 (m, 9H), 0.97–0.89 (m, 6H). ^13^C NMR (100 MHz, CDCl_3_) δ 172.27, 171.17, 171.08, 169.80, 167.08, 167.03, 147.81, 147.07, 146.98, 141.70, 127.28, 127.19, 122.29, 122.22, 117.66, 117.60, 115.10, 115.04, 114.94, 110.21, 110.12, 82.00, 80.92, 58.47, 58.44, 56.02, 56.00, 53.52, 53.39, 46.84, 46.77, 46.74, 46.12, 46.01, 43.49, 31.22, 28.08, 26.09, 24.37, 19.37, 19.33, 18.02. *Some signals in the ^13^C spectrum are doubled due to the presence of both l- and d-configured valine. HRMS calcd for C_30_H_43_N_4_O_8_*m*/*z*: 587.3081 (M – H)^−^, found 587.3088.

#### 4.3.21 *tert*-Butyl (*R*)-4-((*S*)-2-(2-((*E*)-3-(4-Hydroxy-3-methoxyphenyl)acrylamido)acetamido)-3-methylbutanamido)-5-(methoxy(methyl)amino)-5-oxopentanoate (**63**)

Compound **54** (70 mg, 0.201 mmol) was deprotected using general procedure C and coupled to **46** (84 mg, 0.241 mmol) using general procedure B to produce the title compound **63** as an orange solid (40 mg, 34%). ^1^H NMR (400 MHz, MeOD) δ 7.44 (d, *J* = 16.0 Hz, 1H), 7.10 (d, *J* = 2.0 Hz, 1H), 7.01 (dd, *J* = 8.0 Hz, *J* = 1.6 Hz, 1H), 6.76 (d, *J* = 8.0 Hz, 1H), 6.47 (d, *J* = 15.6 Hz, 1H), 4.34–4.22 (m, 2H), 4.06–3.91 (m, 2H), 3.85 (s, 3H), 3.77 (s, 3H), 3.15 (s, 3H), 2.36–2.22 (m, 2H), 2.16–1.92 (m, 2H), 1.89–1.78 (m, 1H), 1.41–1.38 (m, 9H), 0.95–0.90 (m, 6H). ^13^C NMR (100 MHz, CDCl_3_) δ 172.29, 172.08, 171.22, 171.12, 169.46, 169.38, 166.85, 147.54, 146.82, 141.61, 127.29, 122.25, 122.19, 117.62, 114.81, 109.98, 109.91, 80.77, 65.88, 61.64, 58.39, 58.28, 55.92, 48.82, 48.73, 43.46, 43.41, 32.14, 31.23, 28.05, 26.95, 19.35, 19.20, 18.13, 17.87, 15.28. *Some signals in the ^13^C spectrum are doubled due to the presence of both l- and d-configured valine. HRMS calcd for C_28_H_43_N_4_O_9_*m*/*z*: 579.3030 (M + H)^+^, found 579.3036.

#### 4.3.22 *tert*-Butyl (*R*)-4-((*S*)-2-(2-((*E*)-3-(4-Hydroxy-3-methoxyphenyl)acrylamido)acetamido)-3-methylbutanamido)-5-oxo-5-(pyrrolidin-1-yl)pentanoate (**64**)

Compound **55** (65 mg, 0.182 mmol) was deprotected using general procedure C and coupled to **46** (76 mg, 0.218 mmol) using general procedure B to produce the title compound **64** as a yellow solid (30 mg, 28%). ^1^H NMR (400 MHz, MeOD) δ 7.44 (d, *J* = 15.6 Hz, 1H), 7.10 (d, *J* = 2.0 Hz, 1H), 7.01 (dd, *J* = 8.0 Hz, *J* = 1.6 Hz, 1H), 6.76 (d, *J* = 8.4 Hz, 1H), 6.47 (d, *J* = 15.6 Hz, 1H), 4.65–4.60 (m, 1H), 4.34–4.19 (m, 1H), 4.06–3.91 (m, 2H), 3.84 (s, 3H), 3.70–3.59 (m, 2H), 3.44–3.31 (m, 2H), 2.34–2.22 (m, 2H), 2.14–2.04 (m, 1H), 2.02–1.91 (m, 3H), 1.86–1.79 (m, 3H), 1.41–1.38 (m, 9H), 0.94–0.89 (m, 6H). ^13^C NMR (100 MHz, CDCl_3_) δ 172.20, 172.09, 171.01, 169.94, 169.57, 169.42, 166.85, 147.49, 146.81, 141.58, 141.40, 127.35, 122.16, 117.80, 114.79, 109.91, 80.79, 58.13, 55.92, 50.02, 46.60, 46.21, 43.41, 31.35, 30.97, 30.92, 30.88, 28.06, 27.24, 26.01, 24.12, 19.30, 17.99, 17.88. *Some signals in the ^13^C spectrum are doubled due to the presence of both l- and d-configured valine. HRMS calcd for C_30_H_43_N_4_O_8_*m*/*z*: 587.3081 (M – H)^−^, found 587.3078.

#### 4.3.23 Dicyclopentyl ((*E*)-3-(4-Hydroxy-3-methoxyphenyl)acryloyl)glycyl-l-valyl-d-glutamate (**68**)

Compound **67** (108 mg, 0.200 mmol) was deprotected using general procedure A and coupled to *trans*-ferulic acid (43 mg, 0.220 mmol) using general procedure B. The residue was washed twice with diethyl ether to produce the title compound **68** as an off-white solid (93 mg, 76%). ^1^H NMR (400 MHz, CDCl_3_) δ 7.56 (d, *J* = 15.6 Hz, 1H), 7.24 (d, *J* = 7.6 Hz, 1H), 7.09–6.97 (m, 3H), 6.92–6.81 (m, 2H), 6.37 (d, *J* = 15.6 Hz, 1H), 6.00 (s, 1H), 5.21–5.07 (m, 2H), 4.55–4.47 (m, 1H), 4.46–4.40 (m, 1H), 4.23–4.06 (m, 2H), 3.91 (s, 3H), 2.41–2.29 (m, 2H), 2.26–2.08 (m, 2H), 2.06–1.93 (m, 1H), 1.87–1.76 (m, 4H), 1.74–1.51 (m, 12H), 1.00–0.88 (m, 6H). ^13^C NMR (101 MHz, CDCl_3_) δ 172.72, 171.46, 171.10, 169.47, 166.86, 147.57, 146.75, 141.86, 127.23, 122.31, 117.40, 114.73, 109.87, 78.65, 77.57, 58.50, 55.96, 52.03, 43.69, 32.70, 32.65, 32.60, 32.51, 30.80, 30.69, 26.93, 23.70, 23.64, 19.34, 17.74. HRMS calcd for C_32_H_46_N_3_O_9_*m*/*z*: 616.3229 (M + H)^+^, found 616.3221.

#### 4.3.24 Dioctadecyl ((*E*)-3-(4-Hydroxy-3-methoxyphenyl)acryloyl)glycyl-l-valyl-d-glutamate (**72**)

Compound **71** (489 mg, 0.538 mmol) was deprotected using general procedure A and coupled to *trans*-ferulic acid (125 mg, 0.646 mmol) using general procedure B, using DCM instead of DMF as the reaction solvent. Purification by flash chromatography (5% MeOH in DCM) produced the title compound **72** as a yellow solid (376 mg, 71%). ^1^H NMR (400 MHz, CDCl_3_) δ 7.56 (d, *J* = 15.6 Hz, 1H), 7.22 (d, *J* = 7.5 Hz, 1H), 7.06 (dd, *J* = 8.1, 1.9 Hz, 1H), 7.03–6.94 (m, 2H), 6.89 (d, *J* = 8.2 Hz, 1H), 6.77 (t, *J* = 5.2 Hz, 1H), 6.35 (d, *J* = 15.6 Hz, 1H), 5.95 (d, *J* = 7.8 Hz, 1H), 4.60–4.52 (m, 1H), 4.47–4.38 (m, 1H), 4.17–4.06 (m, 4H), 4.02 (t, *J* = 6.8 Hz, 2H), 3.91 (s, 3H), 2.48–2.32 (m, 2H), 2.30–2.13 (m, 2H), 2.10–1.97 (m, 1H), 1.64–1.52 (m, 4H), 1.25 (s, 60H), 1.00–0.91 (m, 6H), 0.88 (t, *J* = 6.8 Hz, 6H). ^13^C NMR (100 MHz, CDCl_3_) δ 173.08, 171.78, 171.11, 169.50, 166.89, 147.59, 146.72, 141.97, 127.19, 122.36, 117.29, 114.73, 109.77, 65.88, 65.09, 58.48, 55.93, 51.99, 43.74, 31.94, 30.70, 30.40, 29.73, 29.68, 29.64, 29.57, 29.54, 29.38, 29.31, 29.26, 29.21, 28.56, 28.49, 26.84, 25.90, 25.81, 22.71, 19.37, 17.65, 14.14. HRMS calcd for C_58_H_102_N_3_O_9_*m*/*z*: 984.7611 (M + H)^+^, found 984.7609.

#### 4.3.25 Diethyl ((*E*)-3-(4-Acetoxy-3-methoxyphenyl)acryloyl)glycyl-l-valyl-d-glutamate (**73**)

Synthesized from **1** (64 mg, 0.120 mmol) and acetyl chloride (10 μL, 0.144 mmol) using general procedure D. Purification by flash chromatography (EtOAc) produced the title compound **73** as a white solid (27 mg, 39%). ^1^H NMR (400 MHz, DMSO-*d*_6_) δ 8.40 (d, *J* = 7.6 Hz, 1H), 8.32 (t, *J* = 5.8 Hz, 1H), 7.95 (d, *J* = 9.0 Hz, 1H), 7.43 (d, *J* = 15.8 Hz, 1H), 7.34 (d, *J* = 1.9 Hz, 1H), 7.17 (dd, *J* = 8.3, 1.8 Hz, 1H), 7.12 (d, *J* = 8.1 Hz, 1H), 6.76 (d, *J* = 15.8 Hz, 1H), 4.31–4.21 (m, 2H), 4.14–3.98 (m, 4H), 3.91 (d, *J* = 5.7 Hz, 2H), 3.82 (s, 3H), 2.35 (t, *J* = 7.5 Hz, 2H), 2.26 (s, 3H), 2.05–1.91 (m, 2H), 1.91–1.77 (m, 1H), 1.21–1.11 (m, 6H), 0.85 (t, *J* = 6.5 Hz, 6H). ^13^C NMR (100 MHz, DMSO-*d*_6_) δ 172.55, 172.05, 171.50, 169.31, 168.94, 165.70, 151.53, 140.65, 138.89, 134.34, 123.75, 122.60, 120.58, 112.06, 61.06, 60.40, 57.82, 56.23, 51.52, 42.68, 31.26, 30.17, 26.37, 20.88, 19.58, 18.23, 14.52, 14.47. HRMS calcd for C_28_H_40_N_3_O_10_*m*/*z*: 578.2708 (M + H)^+^, found 578.2704.

#### 4.3.26 Diethyl ((*E*)-3-(4-(Dodecanoyloxy)-3-methoxyphenyl)acryloyl)glycyl-l-valyl-d-glutamate (**74**)

Synthesized from **1** (64 mg, 0.120 mmol) and lauroyl chloride (34 μL, 0.144 mmol) using general procedure D. Purification by flash chromatography (20% hexanes in EtOAc) produced the title compound **74** as a white solid (45 mg, 52%). ^1^H NMR (400 MHz, CDCl_3_) δ 7.60 (d, *J* = 15.6 Hz, 1H), 7.29 (d, *J* = 7.8 Hz, 1H), 7.13–7.04 (m, 3H), 7.01 (d, *J* = 7.9 Hz, 1H), 6.97–6.90 (m, 1H), 6.48 (d, *J* = 15.6 Hz, 1H), 4.60–4.50 (m, 1H), 4.49–4.41 (m, 1H), 4.21–4.05 (m, 6H), 3.84 (s, 3H), 2.57 (t, *J* = 7.5 Hz, 2H), 2.47–2.32 (m, 2H), 2.26–2.15 (m, 2H), 2.10–1.96 (m, 1H), 1.75 (p, *J* = 7.5 Hz, 2H), 1.47–1.18 (m, 22H), 1.01–0.91 (m, 6H), 0.89 (t, *J* = 7.1 Hz, 3H). ^13^C NMR (100 MHz, CDCl_3_) δ 172.97, 171.74, 171.66, 171.14, 169.35, 166.41, 151.38, 141.22, 141.19, 133.54, 123.21, 120.65, 120.03, 111.60, 61.67, 60.82, 58.52, 55.89, 51.97, 43.69, 34.04, 31.92, 30.83, 30.41, 29.63, 29.52, 29.36, 29.30, 29.06, 26.77, 25.01, 22.70, 19.35, 17.72, 14.14, 14.11. HRMS calcd for C_38_H_60_N_3_O_10_*m*/*z*: 718.4273 (M + H)^+^, found 718.4260.

#### 4.3.27 Diethyl ((*E*)-3-(3-Methoxy-4-(stearoyloxy)phenyl)acryloyl)glycyl-l-valyl-d-glutamate (**75**)

Synthesized from **1** (64 mg, 0.120 mmol) and stearoyl chloride (49 μL, 0.144 mmol) using general procedure D. The residue was washed twice with diethyl ether to produce the title compound **75** (38 mg, 40%). ^1^H NMR (400 MHz, CDCl_3_) δ 7.60 (d, *J* = 15.6 Hz, 1H), 7.30 (d, *J* = 7.6 Hz, 1H), 7.14–7.05 (m, 3H), 7.01 (d, *J* = 7.9 Hz, 1H), 6.95 (t, *J* = 5.2 Hz, 1H), 6.48 (d, *J* = 15.6 Hz, 1H), 4.60–4.50 (m, 1H), 4.49–4.40 (m, 1H), 4.22–4.05 (m, 6H), 3.84 (s, 3H), 2.57 (t, *J* = 7.5 Hz, 2H), 2.45–2.35 (m, 2H), 2.29–2.13 (m, 2H), 2.10–1.98 (m, 1H), 1.79–1.69 (m, 2H), 1.47–1.17 (m, 34H), 1.00–0.92 (m, 6H), 0.88 (t, *J* = 6.6 Hz, 3H). ^13^C NMR (100 MHz, CDCl_3_) δ 172.98, 171.74, 171.69, 171.16, 169.37, 166.43, 151.38, 141.22, 141.19, 133.54, 123.21, 120.65, 120.03, 111.61, 61.68, 60.83, 58.52, 55.89, 51.97, 43.70, 34.04, 31.94, 30.81, 30.41, 29.71, 29.68, 29.65, 29.54, 29.38, 29.31, 29.08, 26.77, 25.01, 22.71, 19.35, 17.71, 14.14, 14.11. HRMS calcd for C_44_H_72_N_3_O_10_*m*/*z*: 802.5212 (M + H)^+^, found 802.5204.

#### 4.3.28 Dicyclopentyl ((*E*)-3-(4-Acetoxy-3-methoxyphenyl)acryloyl)glycyl-l-valyl-d-glutamate (**76**)

Synthesized from **68** (69 mg, 0.112 mmol) and acetyl chloride (10 μL, 0.134 mmol) using general procedure D. Purification by flash chromatography (3% MeOH in DCM) produced the title compound **76** as an off-white solid (59 mg, 80%). ^1^H NMR (400 MHz, CDCl_3_) δ 7.59 (d, *J* = 15.6 Hz, 1H), 7.40 (d, *J* = 7.6 Hz, 1H), 7.25 (d, *J* = 8.8 Hz, 1H), 7.15–7.06 (m, 3H), 7.01 (d, *J* = 8.7 Hz, 1H), 6.51 (d, *J* = 15.6 Hz, 1H), 5.20–5.08 (m, 2H), 4.56–4.42 (m, 2H), 4.20–4.10 (m, 2H), 3.85 (s, 3H), 2.41–2.29 (m, 5H), 2.26–2.08 (m, 2H), 2.03–1.92 (m, 1H), 1.85–1.77 (m, 4H), 1.74–1.60 (m, 8H), 1.59–1.51 (m, 4H), 1.01–0.88 (m, 6H). ^13^C NMR (100 MHz, CDCl_3_) δ 172.66, 171.54, 171.21, 169.40, 168.86, 166.40, 151.32, 140.99, 133.77, 123.15, 120.58, 120.32, 111.73, 78.66, 77.55, 58.54, 55.93, 52.01, 43.61, 32.69, 32.64, 32.60, 32.50, 30.93, 30.69, 26.94, 23.69, 23.63, 20.66, 19.33, 17.84. HRMS calcd for C_34_H_48_N_3_O_10_*m*/*z*: 658.3334 (M + H)^+^, found 658.3311.

#### 4.3.29 Dicyclopentyl ((*E*)-3-(4-(Dodecanoyloxy)-3-methoxyphenyl)acryloyl)glycyl-l-valyl-d-glutamate (**77**)

Synthesized from **68** (60 mg, 0.097 mmol) and lauroyl chloride (28 μL, 0.117 mmol) using general procedure D. The residue was washed twice with hexane to produce the title compound **77** as a white solid (77 mg, 97%). ^1^H NMR (400 MHz, CDCl_3_) δ 7.60 (d, *J* = 15.6 Hz, 1H), 7.17–7.08 (m, 3H), 7.01 (d, *J* = 7.9 Hz, 1H), 6.90 (d, *J* = 8.7 Hz, 1H), 6.78 (t, *J* = 5.1 Hz, 1H), 6.46 (d, *J* = 15.6 Hz, 1H), 5.21–5.09 (m, 2H), 4.56–4.46 (m, 1H), 4.46–4.38 (m, 1H), 4.23–4.06 (m, 2H), 3.85 (s, 3H), 2.57 (t, *J* = 7.5 Hz, 2H), 2.44–2.29 (m, 2H), 2.27–2.08 (m, 2H), 2.05–1.92 (m, 1H), 1.88–1.51 (m, 18H), 1.48–1.38 (m, 2H), 1.36–1.25 (m, 14H), 1.00–0.91 (m, 6H), 0.88 (t, *J* = 6.7 Hz, 3H). ^13^C NMR (100 MHz, CDCl_3_) δ 172.79, 171.70, 171.41, 170.96, 169.22, 166.32, 151.40, 141.29, 141.24, 133.52, 123.21, 120.71, 119.97, 111.57, 78.69, 77.61, 58.48, 55.90, 52.06, 43.67, 34.04, 32.70, 32.65, 32.61, 32.52, 31.91, 30.78, 30.68, 29.62, 29.51, 29.34, 29.28, 29.06, 26.91, 25.01, 23.69, 23.64, 22.69, 19.33, 17.69, 14.12. HRMS calcd for C_44_H_68_N_3_O_10_*m*/*z*: 798.4899 (M + H)^+^, found 798.4876.

#### 4.3.30 Dicyclopentyl ((*E*)-3-(3-Methoxy-4-(stearoyloxy)phenyl)acryloyl)glycyl-l-valyl-d-glutamate (**78**)

Synthesized from **68** (62 mg, 0.101 mmol) and stearoyl chloride (41 μL, 0.121 mmol) using general procedure D. The residue was washed twice with hexane to produce the title compound **78** as a white solid (83 mg, 93%). ^1^H NMR (400 MHz, CDCl_3_) δ 7.60 (d, *J* = 15.6 Hz, 1H), 7.25 (d, *J* = 9.5 Hz, 1H), 7.14–7.04 (m, 3H), 7.00 (d, *J* = 8.2 Hz, 1H), 6.94 (t, *J* = 5.2 Hz, 1H), 6.48 (d, *J* = 15.7 Hz, 1H), 5.22–5.09 (m, 2H), 4.56–4.40 (m, 2H), 4.19–4.13 (m, 2H), 3.84 (s, 3H), 2.57 (t, *J* = 7.5 Hz, 2H), 2.48–2.27 (m, 2H), 2.25–2.09 (m, 2H), 2.06–1.94 (m, 1H), 1.88–1.51 (m, 18H), 1.47–1.37 (m, 2H), 1.37–1.18 (m, 26H), 1.00–0.92 (m, 6H), 0.88 (t, *J* = 6.6 Hz, 3H). ^13^C NMR (100 MHz, CDCl_3_) δ 172.71, 171.70, 171.44, 171.07, 169.30, 166.36, 151.39, 141.20, 141.16, 133.58, 123.19, 120.64, 120.10, 111.66, 78.66, 77.57, 58.51, 55.90, 52.03, 43.64, 35.31, 34.05, 32.69, 32.65, 32.60, 32.52, 31.93, 30.91, 30.68, 29.70, 29.67, 29.64, 29.53, 29.37, 29.30, 29.20, 29.08, 28.88, 26.95, 25.01, 24.24, 23.69, 23.64, 22.70, 19.32, 17.79, 14.13. HRMS calcd for C_50_H_80_N_3_O_10_*m*/*z*: 882.5838 (M + H)^+^, found 882.5810.

#### 4.3.31 Diethyl *O*-Benzyl-*N*-(((*E*)-3-(3-methoxy-4-(stearoyloxy)phenyl)acryloyl)glycyl)-l-seryl-d-glutamate (**79**)

Synthesized from **30** (68 mg, 0.110 mmol) and stearoyl chloride (41 μL, 0.120 mmol) using general procedure D as a white solid (93 mg, 96%). ^1^H NMR (400 MHz, CDCl_3_) δ 7.59 (d, *J* = 15.5 Hz, 1H), 7.39–7.22 (m, 6H), 7.13–6.98 (m, 4H), 6.62 (t, *J* = 5.2 Hz, 1H), 6.41 (d, *J* = 15.6 Hz, 1H), 4.67 (td, *J* = 6.4, 3.8 Hz, 1H), 4.62–4.47 (m, 3H), 4.21–3.98 (m, 6H), 3.97–3.89 (m, 1H), 3.84 (s, 3H), 3.65–3.58 (m, 1H), 2.58 (t, *J* = 7.5 Hz, 2H), 2.43–2.26 (m, 2H), 2.26–2.13 (m, 1H), 2.07–1.94 (m, 1H), 1.76 (p, *J* = 7.5 Hz, 2H), 1.46–1.16 (m, 34H), 0.88 (t, *J* = 6.6 Hz, 3H). ^13^C NMR (101 MHz, CDCl_3_) δ 172.97, 171.70, 171.46, 169.59, 169.07, 166.33, 151.39, 141.44, 141.26, 137.28, 133.47, 128.51, 127.97, 127.84, 123.21, 120.77, 119.80, 111.52, 73.51, 69.18, 61.63, 60.74, 55.90, 52.89, 52.08, 43.60, 34.05, 31.93, 30.24, 29.71, 29.67, 29.64, 29.53, 29.37, 29.30, 29.08, 26.87, 25.02, 22.70, 14.13. HRMS calcd for C_49_H_74_N_3_O_11_*m*/*z*: 880.5318 (M + H)^+^, found 880.5302.

#### 4.3.32 Diethyl ((*E*)-3-(3-Methoxy-4-(stearoyloxy)phenyl)acryloyl)glycyl-l-seryl-d-glutamate (**80**)

Synthesized from **31** (75 mg, 0.140 mmol) and stearoyl chloride (53 μL, 0.160 mmol) using general procedure D. Purification by flash chromatography (6% MeOH in DCM) produced the title compound **80** as a white solid (23 mg, 21%). ^1^H NMR (400 MHz, MeOD) δ 7.57 (d, *J* = 15.6 Hz, 1H), 7.30 (d, *J* = 1.8 Hz, 1H), 7.21 (dd, *J* = 8.2, 1.9 Hz, 1H), 7.06 (d, *J* = 8.2 Hz, 1H), 6.69 (d, *J* = 16.0 Hz, 1H), 4.55–4.44 (m, 2H), 4.23–4.03 (m, 6H), 3.88 (s, 3H), 3.87–3.84 (m, 2H), 2.60 (t, *J* = 7.3 Hz, 2H), 2.51–2.40 (m, 2H), 2.33–2.16 (m, 1H), 2.13–1.98 (m, 1H), 1.81–1.69 (m, 2H), 1.51–1.18 (m, 34H), 0.92 (t, *J* = 7.1 Hz, 3H). ^13^C NMR (100 MHz, CDCl_3_) δ 173.27, 172.12, 171.72, 170.89, 169.57, 166.69, 151.40, 141.53, 141.29, 133.41, 123.22, 120.75, 119.75, 111.61, 61.95, 60.91, 55.90, 54.87, 52.50, 43.75, 34.05, 31.93, 30.53, 29.71, 29.67, 29.53, 29.37, 29.30, 29.08, 25.01, 22.70, 14.13, 14.10. HRMS calcd for C_42_H_68_N_3_O_11_*m*/*z*: 790.4848 (M + H)^+^, found 790.4835.

#### 4.3.33 Diethyl ((*E*)-3-(3-Methoxy-4-(stearoyloxy)phenyl)acryloyl)glycyl-l-threonyl-d-glutamate (**81**)

Synthesized from **32** (65 mg, 0.120 mmol) and stearoyl chloride (49 μL, 0.144 mmol) using general procedure D. Purification by flash chromatography (5% MeOH in DCM) produced the title compound **81** as an off-white solid (35 mg, 36%). ^1^H NMR (400 MHz, CDCl_3_) δ 7.69 (d, *J* = 7.5 Hz, 1H), 7.57 (d, *J* = 15.5 Hz, 1H), 7.53–7.47 (m, 1H), 7.15–7.06 (m, 3H), 7.00 (d, *J* = 8.5 Hz, 1H), 6.47 (d, *J* = 15.5 Hz, 1H), 4.58–4.44 (m, 2H), 4.41 (d, *J* = 6.5 Hz, 1H), 4.21–4.02 (m, 6H), 3.84 (s, 3H), 2.57 (t, *J* = 7.5 Hz, 2H), 2.43 (t, *J* = 7.4 Hz, 2H), 2.29–2.16 (m, 1H), 2.13–1.99 (m, 1H), 1.75 (p, *J* = 7.5 Hz, 2H), 1.49–1.06 (m, 37H), 0.88 (t, *J* = 6.8 Hz, 3H). ^13^C NMR (100 MHz, CDCl_3_) δ 173.16, 172.22, 171.79, 171.29, 170.13, 166.79, 151.38, 141.41, 141.23, 133.46, 123.20, 120.73, 119.83, 111.62, 67.28, 61.97, 60.89, 58.29, 55.88, 52.41, 34.04, 31.94, 30.55, 29.71, 29.68, 29.65, 29.54, 29.38, 29.31, 29.08, 26.16, 25.01, 24.95, 22.71, 18.78, 14.14, 14.08. HRMS calcd for C_43_H_70_N_3_O_11_*m*/*z*: 804.5005 (M + H)^+^, found 804.4999.

#### 4.3.34 Methyl *N*^2^-((*R*)-5-Ethoxy-4-((*S*)-2-(2-((*E*)-3-(4-hydroxy-3-methoxyphenyl)acrylamido)acetamido)-3-methylbutanamido)-5-oxopentanoyl)-*N*^6^-stearoyl-l-lysinate (**82**)

Synthesized from **41** (30 mg, 0.0462 mmol) and stearoyl chloride (14 μL, 0.0462 mmol) using general procedure D. Purification by flash chromatography (7% MeOH in DCM) produced the title compound **82** as an off-white solid (11 mg, 26%). ^1^H NMR (400 MHz, CDCl_3_) δ 7.65–7.54 (m, 2H), 7.37 (d, *J* = 7.3 Hz, 1H), 7.25–7.21 (m, 1H), 7.12–7.06 (m, 1H), 7.04 (d, *J* = 1.9 Hz, 1H), 6.94–6.84 (m, 2H), 6.41 (d, *J* = 15.6 Hz, 1H), 5.84 (s, 1H), 5.75 (d, *J* = 5.4 Hz, 1H), 4.41–4.34 (m, 2H), 4.29–4.12 (m, 3H), 4.03–3.93 (m, 2H), 3.93 (s, 3H), 3.72 (s, 3H), 3.34–3.18 (m, 2H), 2.34 (t, *J* = 6.5 Hz, 2H), 2.22–2.13 (m, 4H), 1.78 (s, 2H), 1.69–1.36 (m, 5H), 1.34–1.16 (m, 33H), 1.01 (d, *J* = 6.8 Hz, 3H), 0.96 (d, *J* = 6.9 Hz, 3H), 0.92–0.84 (m, 3H). ^13^C NMR (100 MHz, DMSO-*d*_6_) δ 173.19, 172.41, 172.20, 171.92, 171.50, 169.42, 166.27, 148.83, 148.28, 139.95, 126.79, 122.05, 118.95, 116.10, 111.36, 60.94, 57.77, 55.98, 52.38, 52.25, 52.17, 38.49, 35.89, 31.75, 31.56, 31.29, 30.98, 29.49, 29.47, 29.40, 29.23, 29.16, 29.12, 27.15, 25.78, 23.23, 22.56, 19.64, 18.23, 14.50, 14.42. HRMS calcd for C_49_H_82_N_5_O_11_*m*/*z*: 916.6005 (M + H)^+^, found 916.6002.

### 4.4 Mice

#### 4.4.1 Experiments with Bone-Marrow-Derived Dendritic Cells and T Cells

C57BL/6, OT I (C57BL/6-Tg(TcraTcrb)1100Mjb/J), and OT II (C57BL/6-Tg(TcraTcrb)425Cbn/Crl) mice were purchased from Jackson Laboratory (Bar Harbor, ME, U.S.A.) and bred at the University of Leiden (The Netherlands). The mice were kept under standard laboratory conditions, with food and water provided *ad libitum*. The mice were euthanized while sedated by cervical dislocation. All animal work was performed according to the guidelines of the European Parliament Directive 2010/63EU, and the experimental work was approved by the Animal Ethics Committee of Leiden University. For culture conditions of BMDCs, see below.

#### 4.4.2 *In Vivo* Experiments

NIH/OlaHsd inbred mice were raised at the Institute of Immunology, Croatia. All mice used were females from 2.0 to 2.5 months old. During the experimental period, the mice were housed in the Animal Facility of the Institute of Immunology, with food and water provided *ad libitum*. All animal work was performed according to the Croatian Law on Animal Welfare (2017), which complies strictly with the EC Directive (2010/63/EU).

### 4.5 Cell Cultures

#### 4.5.1 HEK-Blue NOD1 and NOD2 Cells

HEK-Blue NOD1 and NOD2 cells (InvivoGen, San Diego, CA, U.S.A.) were cultured according to the manufacturer instructions in Dulbecco’s modified Eagle’s medium (Sigma-Aldrich, St. Louis, MO, U.S.A.) supplemented with 10% heat-inactivated fetal bovine serum (Gibco), 2 mM l-glutamine (Sigma-Aldrich), 100 U/mL penicillin (Sigma-Aldrich), 100 μg/mL streptomycin (Sigma-Aldrich), and 100 μg/mL normocin (InvivoGen) for two passages. All subsequent passages were cultured in medium additionally supplemented with 30 μg/mL blasticidin (InvivoGen) and 100 μg/mL Zeocin (InvivoGen). The cells were incubated in a humidified atmosphere at 37 °C and 5% CO_2_.

#### 4.5.2 Peripheral Blood Mononuclear Cells

Human PBMCs from healthy and consenting donors were isolated from heparinized blood by density gradient centrifugation with Ficoll-Paque (Pharmacia, Sweden). The isolated cells were resuspended in RPMI 1640 medium (Sigma-Aldrich, St. Louis, MO, U.S.A.) supplemented with 10% heat-inactivated fetal bovine serum (Gibco), 2 mM l-glutamine (Sigma-Aldrich), 100 U/mL penicillin (Sigma-Aldrich), and 100 μg/mL streptomycin (Sigma-Aldrich) and used in the assays.

#### 4.5.3 Cancer Cell Lines

K562 cells are a chronic myelogenous leukemia cell line (ATCC, Manassas, VA, U.S.A.),^126^ and MEC1 cells are a B-chronic lymphocytic leukemia cell line (DSMZ GmbH, Braunschweig, Germany).^127^ The K562 cells were cultured in RPMI 1640 medium (Sigma-Aldrich, St. Louis, MO, U.S.A.) supplemented with 10% heat-inactivated fetal bovine serum (Gibco), 2 mM l-glutamine (Sigma-Aldrich), 100 U/mL penicillin (Sigma-Aldrich), and 100 μg/mL streptomycin (Sigma-Aldrich). The MEC1 cells were cultured in Iscove’s modified Dulbecco’s medium supplemented with 10% heat-inactivated fetal bovine serum (Gibco), 2 mM l-glutamine (Sigma-Aldrich), 100 U/mL penicillin (Sigma-Aldrich), and 100 μg/mL streptomycin (Sigma-Aldrich).

#### 4.5.4 Bone-Marrow-Derived Dendritic Cells

Bone marrow cells were isolated from the tibia of C57BL/6 mice and cultured in Dulbecco’s modified Eagle’s medium (Lonza, Basel, Switzerland) supplemented with 10% heat-inactivated fetal bovine serum (Lonza), 2 mM l-glutamine (Lonza), 100 U/mL penicillin (Lonza), 100 μg/mL streptomycin (Lonza), and 20 ng/mL granulocyte-macrophage colony-stimulating factor (ImmunoTools, Friesoythe, Germany) for 7 days at 37 °C and 5% CO_2_. The purity of the BMDCs was evaluated with PE-labeled anti-mouse CD11c (Biolegend, San Diego, CA, U.S.A.) by flow cytometry with >90% shown to be CD11c positive.

### 4.6 Cytotoxicity

The tested compounds were dissolved in DMSO and further diluted in culture medium to the desired final concentrations, such that the final DMSO concentration did not exceed 0.1%. HEK-Blue NOD2 cells were seeded (40 000 cells/well) in 96-well plates in 100 μL of culture medium and treated with 20 μM of each compound or with the corresponding vehicle (0.1% DMSO; control cells). After 18 h of incubation (37 °C, 5% CO_2_), the metabolic activity was assessed using the CellTiter 96 Aqueous One Solution cell proliferation assay (Promega, Madison, WI, U.S.A.), according to the manufacturer instructions. The experiments were run in duplicate and repeated as two independent biological replicates.

### 4.7 Measurement of NF-kB Transcriptional Activity (HEK-Blue Detection)

HEK-Blue NOD2 and NOD1 cell line reporter assays are derived from HEK293 cells by cotransfection of the hNOD2 or hNOD1 genes, respectively, and an NF-κB-inducible secreted embryonic alkaline phosphatase (SEAP) reporter gene. Following activation of NOD2 or NOD1, the resulting NF-κB induces production of SEAP, the levels of which can be quantified colorimetrically. HEK-Blue NOD2 or NOD1 cells were seeded (2.5 × 10^5^ cells/mL) in 96-well plates in 200 μL of HEK-Blue detection medium (InvivoGen, San Diego, CA, U.S.A.) and treated with the compounds (2 μM for fixed concentration assay; 7–8 different concentrations from 1 nM to 20 μM for EC_50_ determination) or with the corresponding vehicle (0.1% DMSO). After 18 h of incubation (37 °C, 5% CO_2_), secreted embryonic alkaline phosphatase (SEAP) activity was determined spectrophotometrically as absorbance at 630 nm (BioTek Synergy microplate reader; Winooski, VT, U.S.A.). EC_50_ values were calculated using Prism software (version 9; GraphPad Software, CA, U.S.A.). For determination of specificity, HEK-Blue NOD2 cells (2.5 × 10^5^ cells/mL) were first pretreated for 1 h with a 10 μM NOD2 antagonist, before the addition of the compounds (2 μM), with incubation for 18 h. SEAP activity in the supernatants was determined as above. The experiments were run in duplicate and repeated as at least two independent biological replicates.

### 4.8 Cytokine Release from Peripheral Blood Mononuclear Cells

Peripheral blood mononuclear cells were seeded (1 × 10^6^ cells/mL) in 48-well plates in 500 μL of growth medium and treated with the compounds (2 μM) or with the corresponding vehicle (0.1% DMSO) in the absence and presence of LPS (10 ng/mL). Cell-free supernatants were collected after 18 h of incubation (37 °C, 5% CO_2_) and stored at −80 °C until tested. Cytokine production was determined with the BD Cytometric Bead Array Human Inflammatory Cytokines Kit (contents: IL-8, IL-1β, IL-6, IL-10, TNF, IL-12p70; BD Bioscience) on an Attune NxT flow cytometer (Thermo Fisher Scientific, Waltham, MA, U.S.A.). Standard curves were generated using recombinant cytokines contained in the kit. The data were analyzed using the FlowJo (Tree Star, Inc., Ashland, OR, U.S.A.) and Prism (GraphPad, San Diego, CA, U.S.A.) software. The experiments were repeated as four independent biological replicates. Statistical significance was determined by repeated measures one-way ANOVA followed with Dunnett’s multiple comparisons test.

### 4.9 Peripheral Blood Mononuclear Cell Cytotoxicity

The PBMC cytotoxicity assays using K562 and MEC1 cells were performed as described previously, with some modifications.^89^ PBMCs were seeded (4 × 10^5^ cells/well) in 96-well plates and treated with compounds (0.1–10 μM) or vehicle (0.1% DMSO) for 18 h. The K562 or MEC1 cells were stained with CFSE (Invitrogen, Carlsbad, CA, U.S.A.), washed twice with complete medium, and added (1 × 10^4^ cells/well) to pretreated PBMCs for a final effector cell to target tumor cell ratio of 40:1. After a 4 h co-incubation (37 °C, 5% CO_2_), cells were stained with Sytox blue dead cell stain (Invitrogen) and analyzed using an Attune NxT flow cytometer (Thermo Fisher Scientific, Waltham, MA, U.S.A.) and the FlowJo software (Tree Star, Inc., Ashland, OR, U.S.A.). Cells that were positive for both CFSE and Sytox blue were defined as dead K562 and MEC1 cells. PBMCs alone and the CFSE-labeled cancer cells alone were also treated with the compounds at the same concentrations and stained with Sytox blue to determine any direct cytotoxicity of the compounds toward the PBMCs and cancer cells. The experiments were run in duplicate and repeated as three independent biological replicates. Statistical significance was determined by one-way ANOVA followed with Dunnett’s multiple comparisons test.

### 4.10 RNA Sequencing

Peripheral blood mononuclear cells from three independent donors were seeded (2 × 10^6^ cells/mL) in 24-well plates in 1 mL of growth medium and treated with the compounds (2 μM) or vehicle (0.1% DMSO) for 18 h at 37 °C in 5% CO_2_. The cells were washed with phosphate-buffered saline, resuspended in RNAlater RNA stabilization solution (Sigma-Aldrich, St. Louis, MO, U.S.A.), and stored at −80 °C. RNA extraction, library construction, and sequencing were conducted by Genewiz (Leipzig, Germany).

Briefly, total RNA was extracted using the RNeasy mini kit (Qiagen, Hilden, Germany) according to the manufacturer protocol. RNA samples were quantified using Qubit 4.0 Fluorometer (Life Technologies, Carlsbad, CA, U.S.A.), and RNA integrity was checked with RNA Kit on a 5600 Fragment Analyzer (Agilent Technologies, Palo Alto, CA, U.S.A.). All RNA samples were of high quality with an RNA quality number of ≥9.4. RNA sequencing libraries were prepared using NEBNext Ultra II RNA library prep kit for Illumina according to the manufacturer instructions (New England Biolabs, Ipswich, MA, U.S.A.). Libraries were loaded on the Illumina NovaSeq 6000 instrument, and clustering was performed directly on the NovaSeq before sequencing according to the manufacturer instructions. The samples were sequenced using a 2 × 150 paired end configuration. Image analysis and base calling were conducted by the NovaSeq Control Software. Raw sequence data (.bcl files) generated from the Illumina NovaSeq were converted into *fastq* files and demultiplexed using Illumina’s bcl2fastq 2.19 software. One mismatch was allowed for index sequence identification. After investigating the quality of the raw data, sequence reads were trimmed to remove possible adapter sequences and nucleotides with poor quality using Trimmomatic v.0.36. The trimmed reads were mapped to the *Homo sapiens* reference genome as available on ENSEMBL, using STAR aligner v.2.5.2b, thus generating BAM files. Unique gene hit counts were calculated using feature counts from the Subread package v.1.5.2. Only unique reads that fell within exon regions were counted. After extraction of gene hit counts, the gene hit counts table was used for downstream differential expression analysis.

Differential expression analysis was performed with iDEP.91.^128^ First, a low expression filter was applied (0.5 counts per million in at least 1 library). The remaining gene counts were normalized by counts per million in the EdgeR package, with a pseudocount of 4. Differential gene expression analysis was performed with the DESeq2 method, using a false discovery rate <0.05 and a gene expression fold change >1.5 or <0.667 as the cutoff values. The list of differentially expressed genes was then used as input for gene annotation and pathway enrichment analysis with Metascape.^94^

### 4.11 Bone-Marrow-Derived Dendritic Cell Antigen Presentation

CD4^+^ and CD8^+^ T cells were purified from splenocytes of OT II and OT I transgenic mice using CD4^+^ and CD8^+^ T cell isolation kits (Miltenyi Biotec, Germany), according to manufacturer instructions. Purified T cells were stained with CFSE (Invitrogen, Carlsbad, CA, U.S.A.) and washed. Then, 5 × 10^4^ T cells were mixed with 1 × 10^4^ BMDCs per well (pretreated with desmuramylpeptides [1 or 10 μM] and 50 μg/mL ovalbumin [InvivoGen, San Diego, CA, U.S.A.] for 18 h, and then washed). After 72 h of co-incubation (37 °C, 5% CO_2_), the supernatants were collected and stored at −80 °C for subsequent cytokine measurements. The cells were stained with Fixable viability dye eFluor 780 (eBioscience, Thermo Fisher Scientific, MA, U.S.A.), anti-Thy1.2-PE-Cy7 antibodies (Biolegend, San Diego, CA, U.S.A.), anti-CD8-eFluor450 antibodies (eBioscience), anti-CD4-eFluor450 antibodies (eBioscience), and anti-CD25-APC antibodies (Biolegend) and analyzed using a Beckman Coulter Cytoflex S flow cytometer (CA, U.S.A.) and FlowJo software (Tree Star, Inc., Ashland, OR, U.S.A.). Live Thy1.2^+^/CD4^+^ and Thy1.2^+^/CD8^+^ were evaluated for CFSE dilution and CD25 expression. The experiments were run in duplicate and repeated as two independent biological replicates. Statistical significance was determined by one-way ANOVA followed by Dunnett’s multiple comparisons test.

### 4.12 T Cell Cytokine Release

Supernatants from CD4^+^ and CD8^+^ T cells after 72 h of co-incubation with BMDCs (pretreated with desmuramylpeptides [10 μM] and washed) were collected as described above. The cytokine concentrations were determined with the Cytometric Bead Array Mouse Th1/Th2/Th17 Cytokine Kit (contents: IL-2, IL-4, IL-6, IFN-γ, TNF, IL-17A, IL-10; BD Bioscience) on an Attune NxT flow cytometer (Thermo Fisher Scientific, Waltham, MA, U.S.A.). Standard curves were generated using recombinant cytokines contained in the kit. The data were analyzed using the FlowJo (Tree Star, Inc., Ashland, OR, U.S.A.) and Prism (GraphPad, San Diego, CA, U.S.A.) software. The experiments were run in duplicate and repeated as two independent biological replicates.

### 4.13 *In Vivo* Induction of Ovalbumin-Specific Immune Response

#### 4.13.1 Materials, Antigens, and Antibodies

Bovine serum albumin, Tween 20, monoclonal anti-chicken egg albumin (clone OVA-14 mouse IgG1 isotype), *o*-phenylenediaminedihydrochloride, and MDP were from Sigma (U.S.A.). Horseradish-peroxidase-conjugated goat anti-mouse IgG (HRP-anti-mouse IgG) was from Bio-Rad Laboratories (U.S.A.). Biotin-conjugated rat anti-mouse IgG1 and anti-mouse IgG2a monoclonal antibodies and streptavidin–peroxidase were from PharMingen, Becton Dickinson (U.S.A.). Chemicals for buffers and solutions were from Kemika (Croatia). Ovalbumin was from Serva (Germany). l-α-Phosphatidylcholine, type XI-E, from fresh egg yolk (egg-phosphatidylcholine) was from Avanti Polar Lipids. Cholesterol from porcine liver and dicetylphosphate were from Sigma (U.S.A.). Monomannosyl-PEG-palmitic acid derivative (Man-PEG-Pam) was synthesized as previously described.^129^

#### 4.13.2 Preparation of Liposomes

Multilamellar liposomes were prepared by the modified thin lipid film method as described previously.^130,131^ For the preparation of neutral liposomes, a 7:5 molar ratio of egg-phosphatidylcholine and cholesterol was used. For the preparation of negatively charged liposomes, a 7:5:1 molar ratio of egg-phosphatidylcholine, cholesterol, and dicetylphosphate was used, while a 7:5:0.5 molar ratio of egg-phosphatidylcholine, cholesterol, and Man-PEG-Pam was used in the preparation of mannosylated liposomes. Lipids (total lipid concentration, 4 mg/mL) were dissolved in chloroform/methanol (2:1). A methanol or chloroform/methanol solution of MDP or the desmuramylpeptides was added to the lipid solution for a final concentration of 2 mM and 1.5 mM for the first (Figure 10) and second (Figure 11) experiments, respectively. After rotary evaporation of the solvent, the remaining lipid film was dried in vacuum for 1 h and then dispersed by gentle handshaking in an ovalbumin solution in saline (0.0667 and 0.1 mg/mL for the first and second experiments, respectively). This was then left overnight at 4 °C to swell and stabilize. Liposome size was reduced by sonification. Nonentrapped material was not separated from liposomes, and the complete liposome suspension was used for immunizations.

#### 4.13.3 Immunizations

For the first experiment (Figure 10), sex-matched NIH/OlaHsd mice (five per group) were immunized subcutaneously in the tail base and boosted one time after 21 days. The injection volume in all experimental groups was 0.15 mL per mouse, which corresponds to 10 μg of OVA, 400 μg of lipids, and 0.30 μmol of MDP and desmuramylpeptides. For the second experiment (Figure 11), sex-matched NIH/OlaHsd mice (five per group) were immunized and boosted two times subcutaneously into the tail base at 21-day intervals. The injection volume in all experimental groups was 0.1 mL per mouse, which corresponds to 10 μg of OVA, 400 μg of lipids, and 0.15 μmol of MDP and **75**. The mice were anesthetized with ip application of ketamine/xylazine (25 mg/kg each) prior to blood collection from the axillary plexus on the seventh day after the last booster dose. Individual serum from each animal was decomplemented at 56 °C for 30 min and then stored at −20 °C until tested.

#### 4.13.4 Ovalbumin-Specific Serum Antibody Concentration Determination by ELISA

ELISA assays were performed as detailed previously.^46^ Briefly, high-binding ELISA plates (Costar, U.S.A.) were coated with a 15 μg/mL solution of ovalbumin in carbonate buffer, pH 9.6, and incubated overnight at room temperature. Nonspecific antibody binding was blocked with 0.5% w/v bovine serum albumin in PBS-T (0.05% (v/v) Tween 20 in phosphate-buffered saline) for 2 h at 37 °C. After washing, five serial dilutions of mice sera and standard preparations were added in duplicate. Plates were incubated overnight at room temperature, washed, and analyzed for ovalbumin-specific IgG levels by incubation with HRP-conjugated goat anti-mouse IgG (2 h at 37 °C) and then, after washing, with 0.6 mg/mL *o*-phenylenediaminedihydrochloride solution in citrate-phosphate buffer, pH 5.0, with 0.5 μL of 30% H_2_O_2_ per milliliter for 30 min at room temperature in the dark. The enzymatic reaction was stopped with 12.5% H_2_SO_4_, and absorbance at 492 nm was measured using a microplate reader (Thermo Fisher Scientific, Waltham, MA, U.S.A.). For the determination of ovalbumin-specific IgG1 and IgG2a, the plates were incubated with biotin-conjugated rat anti-mouse IgG1 or IgG2a (2 h at 37 °C) and subsequently with streptavidin–peroxidase for another 2 h at 37 °C. After washing, the substrate solution was added and incubated for 30 min at room temperature in the dark, as described above. The enzymatic reaction was stopped with 12.5% H_2_SO_4_, and absorbance at 492 nm was measured using a microplate reader. The relative quantities of antibodies were determined by parallel line assays using appropriate standard preparations of anti-ovalbumin IgG, anti-ovalbumin IgG1, and anti-ovalbumin IgG2a. Statistical significance was determined by one-way ANOVA followed by Dunnett’s multiple comparisons test.

### 4.14 Screening against PAINS

All tested compounds were screened against the PAINS filter^132^ as implemented in CANVAS (Schrödinger software, Release 2020-2, New York, U.S.A.). Compound **21** was identified as an interfering compound due to the presence of a catechol structure. Given that **21** was tested in two independent analogous assays with HEK-Blue NOD1 and NOD2 cells and it showed no activity in the NOD1 assay, the activity measured in the NOD2 assay did not arise from nonspecific activation. Furthermore, the reduced NOD2 activation of **21** after NOD2 antagonist pretreatment in the specificity assay provided an additional argument for the absence of interference of the catechol structure in **21**.

### 4.15 Statistics

Data analysis was performed using Prism software (version 9; GraphPad Software, CA, U.S.A.). Statistical differences were determined as specified under individual experimental procedures. A *p* value < 0.05 was considered statistically significant.

## Supporting Information Available

(Molecular formula strings CSV)
Synthesis and analytical data of intermediate compounds **4**, **5**, **7**–**17**, **22**, **33**–**40**, **47**–**55**, **65**–**67**, and **69**–**71**, supporting schemes and figures, experimental procedure for chiral HPLC resolution of compound **29**, ^1^H–^1^H NOESY determination of cyclopropane stereoconfiguration in compounds **27**, **28**, **29a**, and **29b**, and ^1^H and ^13^C NMR spectra of representative tested compounds (PDF)

## Abbreviations Used

BMDC: bone-marrow-derived dendritic cell
CARD: caspase recruitment domain
CFSE: carboxyfluorescein succinimidyl ester
COMU: (1-cyano-2-ethoxy-2-oxoethylidenaminooxy)dimethylamino-morpholino-carbenium hexafluorophosphate
DBU: 1,8-diazabicyclo[5.4.0]undec-7-ene
DC: dendritic cell
DCC: dicyclohexylcarbodiimide
DIPEA: *N*,*N*-diisopropylethylamine
DMAP: 4-dimethylaminopyridine
DMF: dimethylformamide
EDC: 1-ethyl-3-(3-(dimethylamino)propyl)carbodiimide
EtOAc: ethyl acetate
GO: Gene Ontology
HOBt: 1-hydroxybenzotriazole
ISG: interferon-stimulated gene
KEGG: Kyoto Encyclopedia of Genes and Genomes
LPS: lipopolysaccharide
Man-PEG-Pam: monomannosyl–poly(ethylene glycol)–palmitic acid
MDP: muramyl dipeptide
MHC: major histocompatibility complex
MurNAc: *N*-acetylmuramic acid
NF-κB: nuclear factor κB
NK: natural killer
NOD: nucleotide-binding oligomerization domain
PBMC: peripheral blood mononuclear cell
PRR: pattern recognition receptor
SEAP: secreted embryonic alkaline phosphatase
TFA: trifluoroacetic acid
THF: tetrahydrofuran
TLR: Toll-like receptor
