# Supplementary material for: Structural Fine-Tuning of Desmuramylpeptide NOD2 Agonists Defines Their In Vivo Adjuvant Activity
Source: J Med Chem. 2021 May 27;64(11):7809–38. doi: 10.1021/acs.jmedchem.1c00644 (PMC8279416; doi:10.1021/acs.jmedchem.1c00644)
Supplement: Supplementary file 2 — jm1c00644_si_002.pdf [file jm1c00644_si_002.pdf]

SUPPORTING INFORMATION

Structural fine-tuning of desmuramylpeptide NOD2 agonists defines their *in vivo* adjuvant activity

Samo Guzelj,[1] Sanja Nabergoj,[1] Martina Gobec,[1] Stane Pajk,[1] Veronika Klančič,[1] Bram Slütter,[2] Ruža Frkanec,[3] Adela Štimac,[3] Primož Šket,[4] Janez Plavec,[4] Irena Mlinarič-Raščan,[1] Žiga Jakopin[*,1]

[1]Faculty of Pharmacy, University of Ljubljana, SI-1000 Ljubljana, Slovenia

[2]Div. BioTherapeutics, Leiden Academic Centre for Drug Research, Leiden University, 2333 CC Leiden, The Netherlands

[3]Centre for Research and Knowledge Transfer in Biotechnology, University of Zagreb, 10000 Zagreb, Croatia

[4]Slovenian NMR Centre, National Institute of Chemistry, SI-1000 Ljubljana, Slovenia

*Corresponding author:

Žiga Jakopin

Phone: +386 1 4769 646

Fax: + 386 1 4258 031

E-mail address: ziga.jakopin@ffa.uni-lj.si

Table of contents:

1 Supporting Schemes 3

2 Supporting figures 4

3 Supporting tables 6

4 Chiral resolution of compound 29 6

5 ^{1}H–^{1}H NOESY determination of cyclopropane stereoconfiguration of compounds 27, 28, 29a, and 29b 8

6 Synthesis and characterization of compounds 12

7 ^{1}H and ^{13}C NMR spectra of representative tested compounds 25

8 Select UHPLC traces 40

9 References 44

1 Supporting Schemes

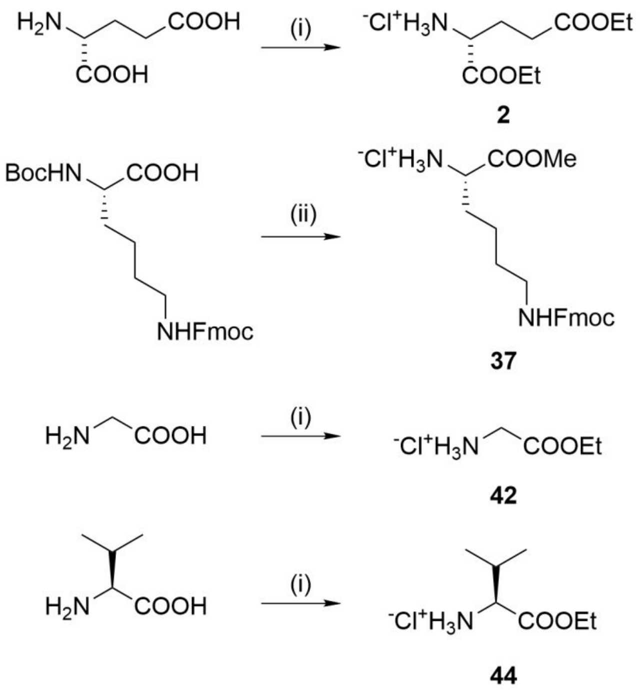

Supporting scheme S1. Synthesis of compounds **2**, **37**, **42**, and **44**. Reagents and conditions: (i) $SOCl_2$, EtOH, reflux; (ii) $SOCl_2$, MeOH, rt.

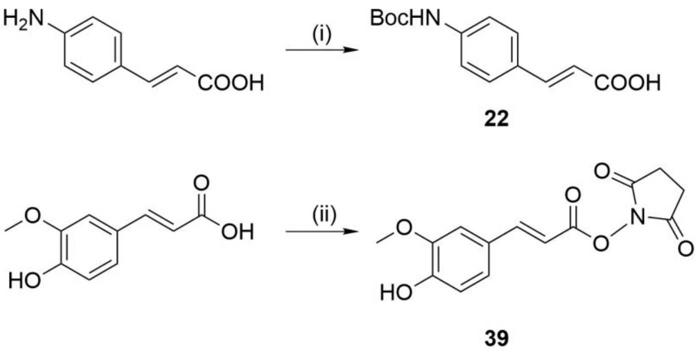

Supporting scheme S2. Synthesis of compounds **22** and **39**. Reagents and conditions: (i) Boc_2O, NaOH, H_2O/dioxane, rt; (ii) *N*-Hydroxysuccinimide, EDC, DMAP, THF/MeCN, rt.

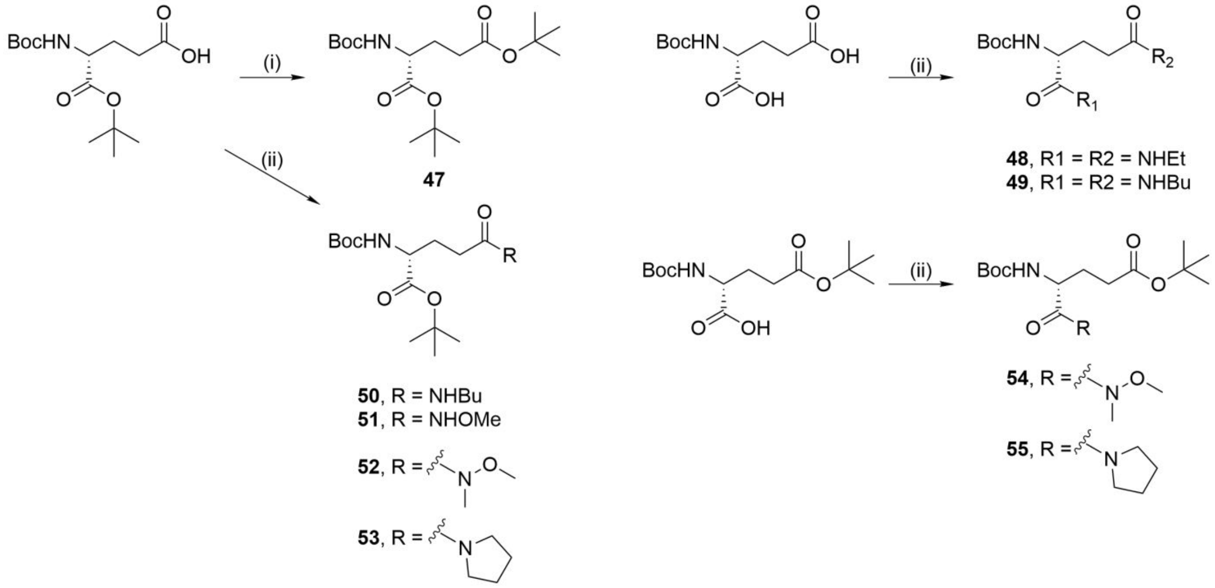

Supporting scheme S3. Synthesis of compounds **47** – **55**. Reagents and conditions: (i) Tert-butyl 2,2,2-trichloroacetimidate, boron trifluoride diethyl etherate, DCM/cyclohexane, rt; (ii) RH, EDC, HOBt, Et_3N, DMAP, DMF, rt.

2 Supporting figures

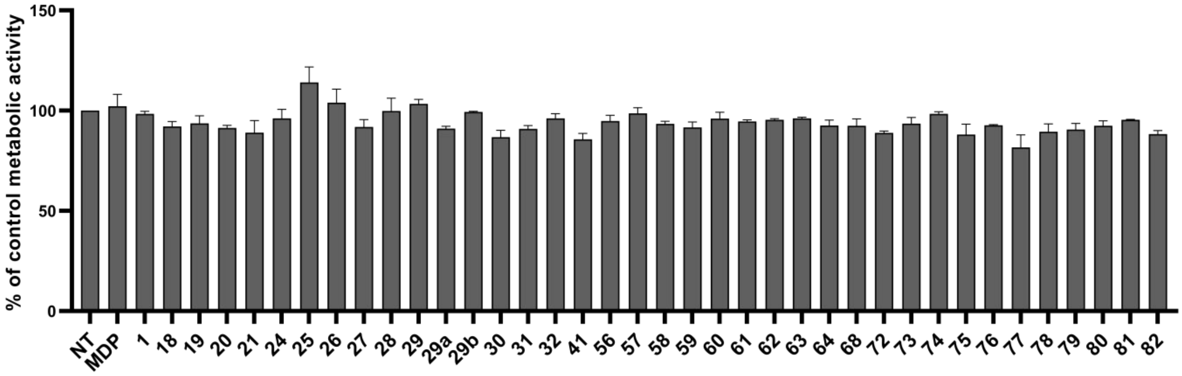

Supporting figure S1. Metabolic activities of HEK-Blue NOD2 cells after 18 h stimulation with MDP (20 µM) or the desmuramylpeptides (20 µM). The results are shown relative to that of the untreated control (0.1 % DMSO; NT). Data are means ±SEM of two independent experiments.

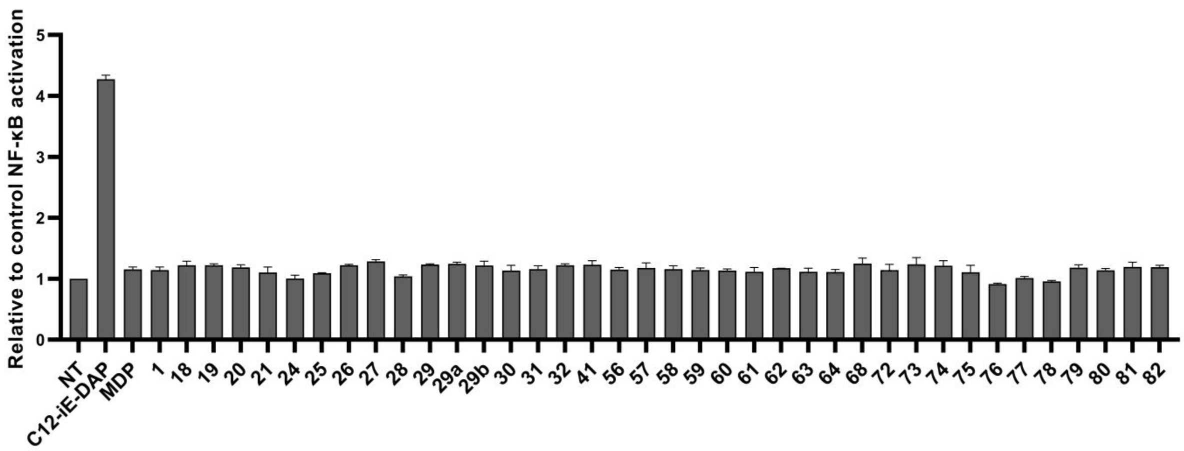

Supporting figure S2. NOD1 agonistic activities of desmuramylpeptides. HEK-Blue NOD1 cells were treated with C12-iE-DAP (100 nM) or the desmuramylpeptides (2 μM) for 18 h. The SEAP activities are shown relative to that of the untreated control (0.1 % DMSO; NT). Data are means ±SEM of two independent experiments. C12-iE-DAP was used as the positive control.

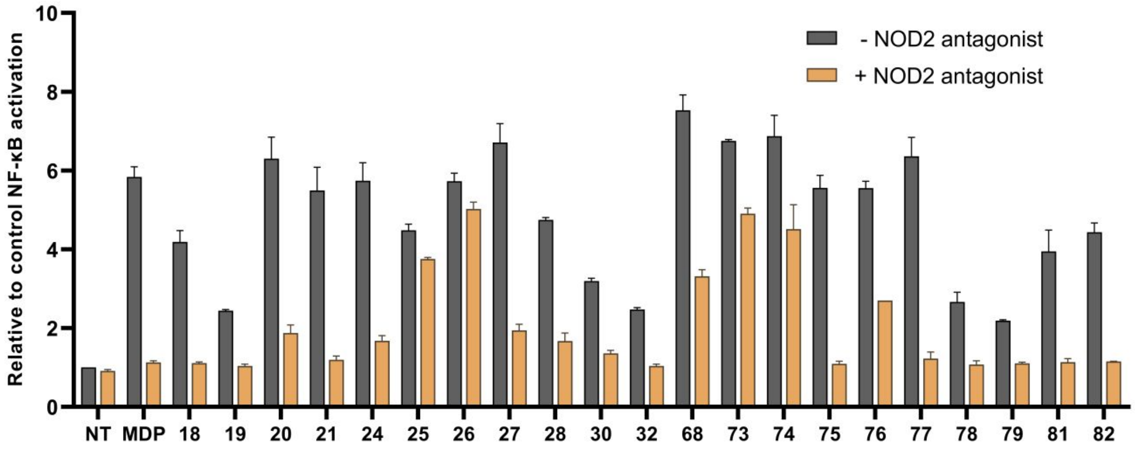

Supporting figure S3. Effects of NOD2 antagonist pre-treatment on MDP- and desmuramylpeptide-induced NF-kB transcriptional activities. HEK-Blue NOD2 cells were pre-incubated for 1 h in the presence or absence of a NOD2 antagonist (10 μM), before the addition of MDP (2 μM) or desmuramylpeptides (2 μM). The SEAP activities after 18 h of incubation are shown relative to that of the untreated control (0.1 % DMSO; NT). Data are means ±SEM of two independent experiments.

3 Supporting tables

Supporting table S1. Ratio of ovalbumin-specific IgG1 and IgG2a levels in the first *in vivo* experiment*

Experimental group	Log_{10} (anti-OVA IgG1/anti-OVA IgG2a)**
OVA	1.95 ± 0.20
OVA + MDP	2.43 ± 0.25
OVA + **1**	1.68 ± 0.06
OVA + **68**	2.28 ± 0.13
OVA + **74**	2.90 ± 0.39
OVA + **75**	2.07 ± 0.17
OVA + **81**	2.18 ± 0.24

*The IgG1/IgG2a ratios were calculated one week after the booster dose. Refer to Figure 10 for ovalbumin-specific IgG, IgG1, and IgG2a levels. **Data are means ±SEM of 5 mice per group.

Supporting table S2. Ratio of ovalbumin-specific IgG1 and IgG2a levels in the second *in vivo* experiment*

Experimental group		Log_{10} (anti-OVA IgG1/anti-OVA IgG2a)**
OVA	Neutral liposomes	2.73 ± 0.29
	Anionic liposomes	1.45 ± 0.25
	Mannosylated liposomes	2.83 ± 0.23
OVA + MDP	Neutral liposomes	3.02 ± 0.17
	Anionic liposomes	2.85 ± 0.10
	Mannosylated liposomes	2.48 ± 0.19
OVA + **75**	Neutral liposomes	2.27 ± 0.17
	Anionic liposomes	1.84 ± 0.20
	Mannosylated liposomes	2.15 ± 0.17

*The IgG1/IgG2a ratios were calculated one week after the second booster dose. Refer to Figure 11 for ovalbumin-specific IgG, IgG1, and IgG2a levels. **Data are means ±SEM of 5 mice per group.

4 Chiral resolution of compound 29

Semipreparative normal-phase chiral HPLC separation of compound **29** was performed on an Agilent Technologies/HP 1100 series system equipped with an autosampler, a binary pump system, a photodiode array detector, a thermostated column compartment, a fraction collector compartment, and the Agilent ChemStation data system. The column used was a Kromasil 5-CelluCoat column (5 µm, 10 mm × 250 mm). A solution of compound **29** (200 µL, 2.5 mg/mL in ethanol) was injected

and eluted over 16 min at a flow rate of 3 mL/min, using a mixture of hexane (80 %) and isopropanol (20 %). The column was thermostated at 25 °C. The separation was repeated twelve times, after which the eluates corresponding to the two chromatographic peaks were pooled and evaporated to dryness, yielding 1.65 mg of the first-eluted **29a** as a white solid and 1.76 mg of the second-eluted **29b** as a white solid.

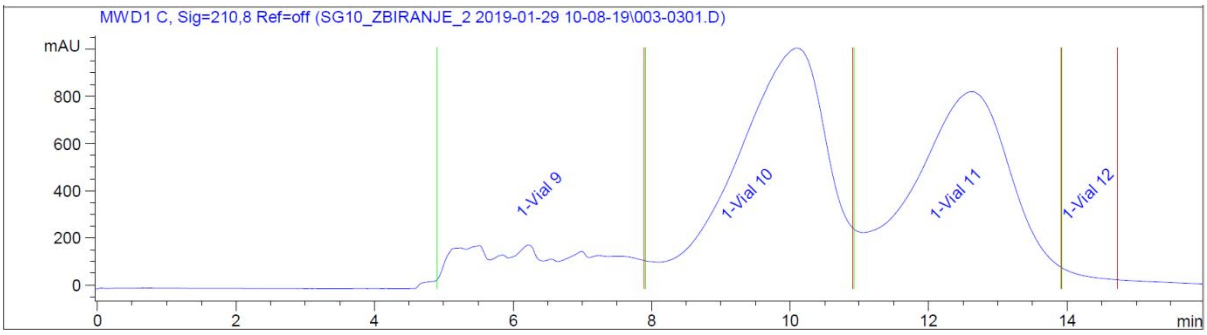

Supporting figure S4. Semipreparative chiral HPLC of the racemic compound 29.

The enantiomeric excess (ee) of the separated diastereomers was evaluated with analytical normal-phase chiral HPLC with a Kromasil 3-CelluCoat column (3 µm, 4.6 mm × 150 mm). A solution of compounds **29a** and **29b** (20 µL, 0.2 mg/mL in ethanol) was injected and eluted over 25 min at a flow rate of 1 mL/min, using a mixture of hexane (95 %) and ethanol (5 %). The ee of both compounds was determined to be > 99 %.

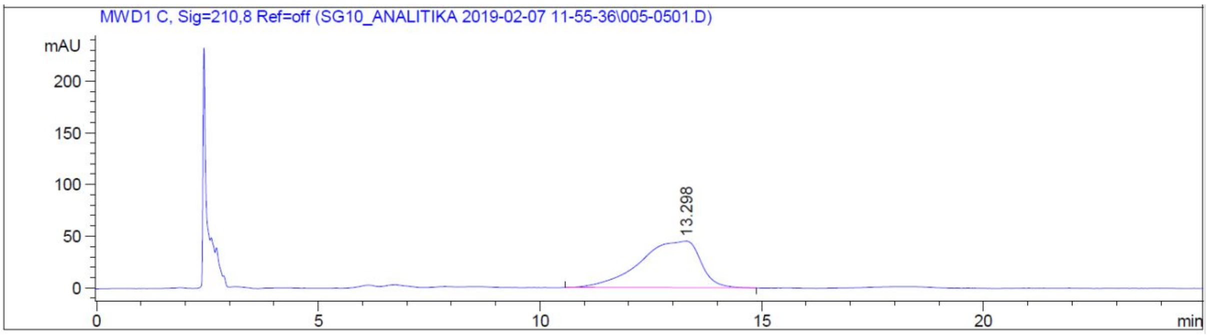

Supporting figure S5. Analytical chiral HPLC of compound **29a**.

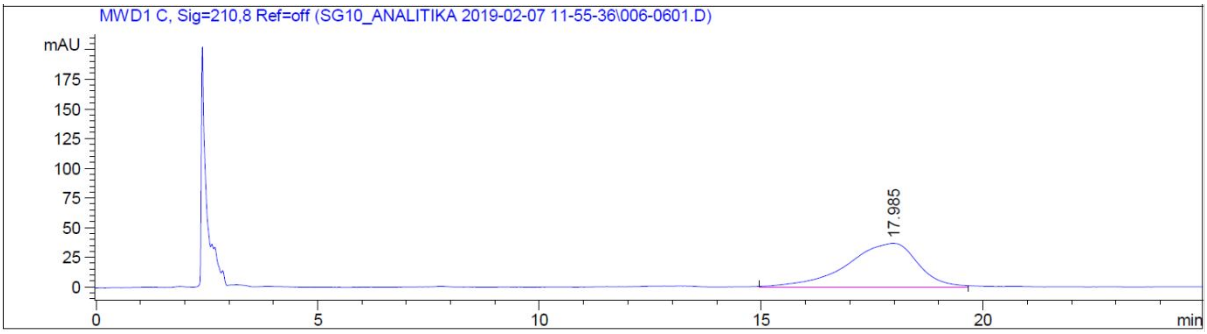

Supporting figure S6. Analytical chiral HPLC of compound **29b**.

5 ^1H–^1H NOESY determination of cyclopropane stereoconfiguration of compounds 27, 28, 29a, and 29b

^1H spectra of compounds **27**, **28**, **29a,** and **29b** were first assigned using homonuclear correlation spectroscopy (COSY) and total correlation spectroscopy (TOCSY) (not shown). ^1H–^1H NOESY spectra of these compounds were then recorded to determine the arrangement of protons on the cyclopropane ring. The presence of H(18)–H(29’) and H(19)–H(29’’) NOE contacts in compounds **27** and **28** suggested a *trans* orientation of the substituents on the cyclopropane ring (Figures S7 and S8). Conversely, if the ring were *cis* configured, both H(18) and H(19) would form contacts with the same proton of the cyclopropane –CH_2(29)– methylene group (i.e. either H(29’) or H(29’’)). Analogously, H(18)–H(20’) and H(19)–H(20’’) contacts were found in the NOESY spectra of compounds **29a** and **29b**, confirming the same *trans* configuration. As there were no differences in the ^1H chemical shifts of **29a** and **29b**, both NOESY spectra were superimposed in figure S9, for added clarity.

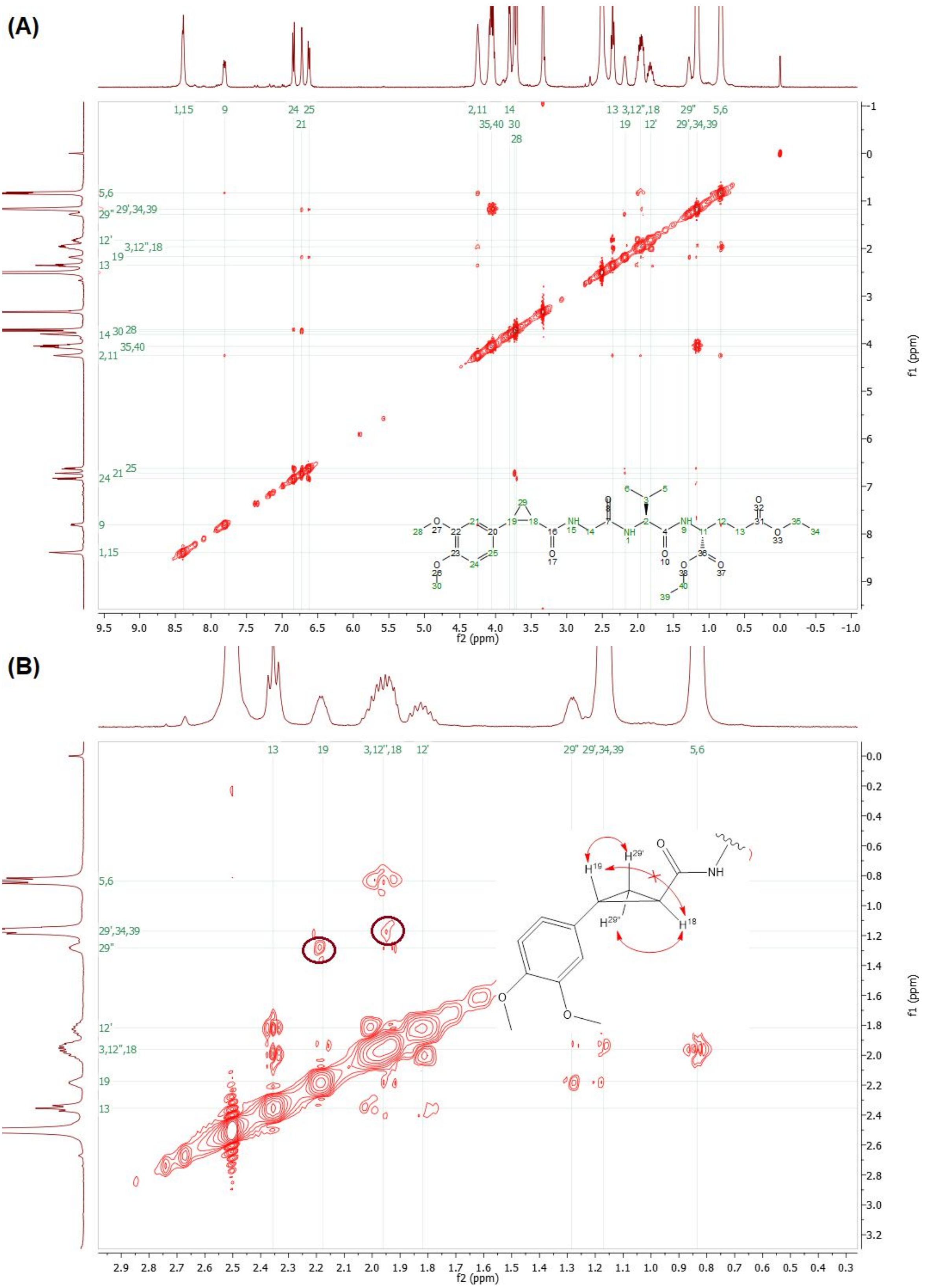

Supporting figure S7. (A) 400 MHz ^{1}H–^{1}H NOESY spectrum of compound **27** in DMSO-d_6. (B) Enlarged region showing the relevant NOE contacts (circled in dark red).

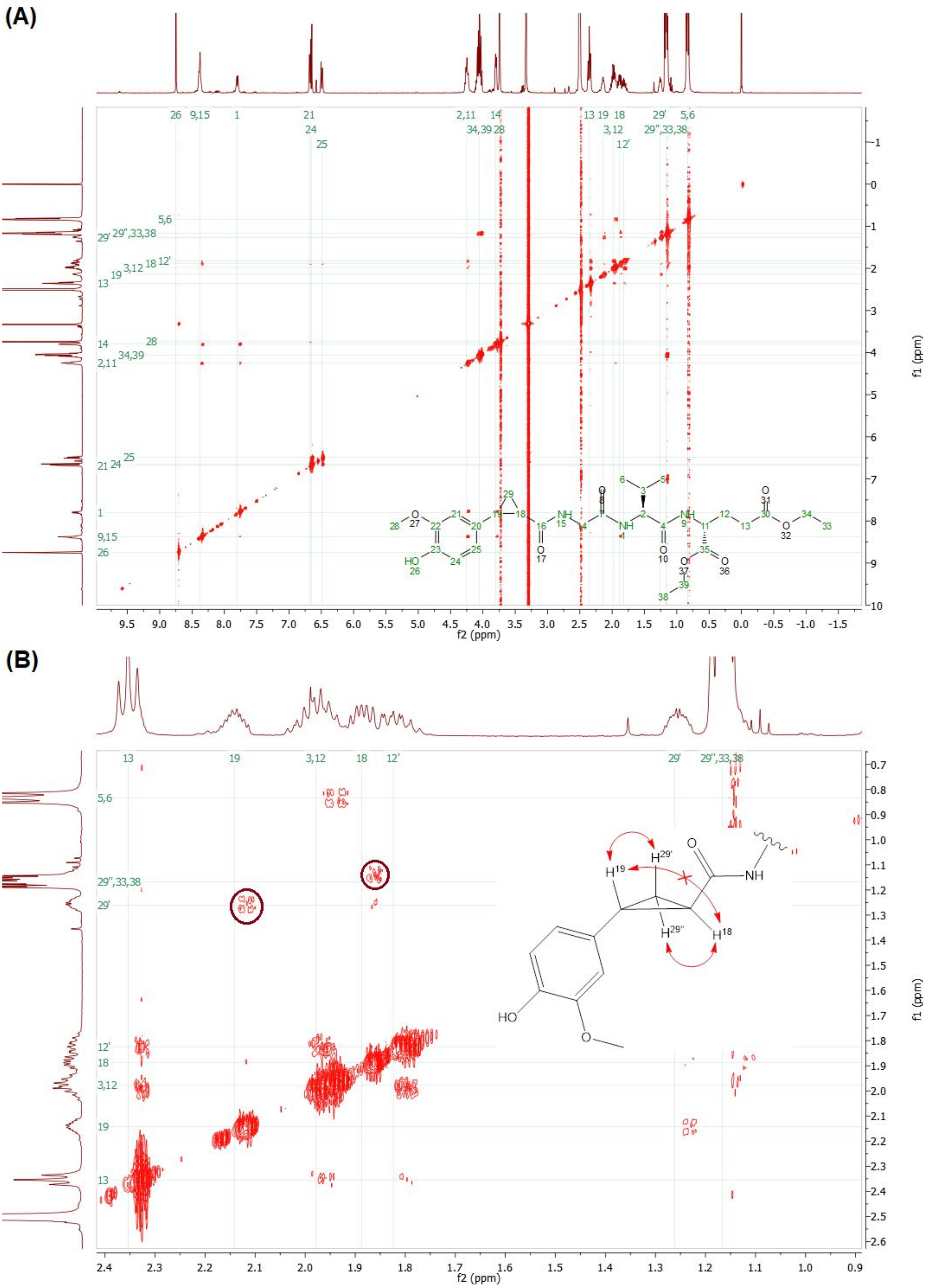

Supporting figure S8. (A) 800 MHz ^{1}H–^{1}H NOESY spectrum of compound **28** in DMSO-d_6. (B) Enlarged region showing the relevant NOE contacts (circled in dark red).

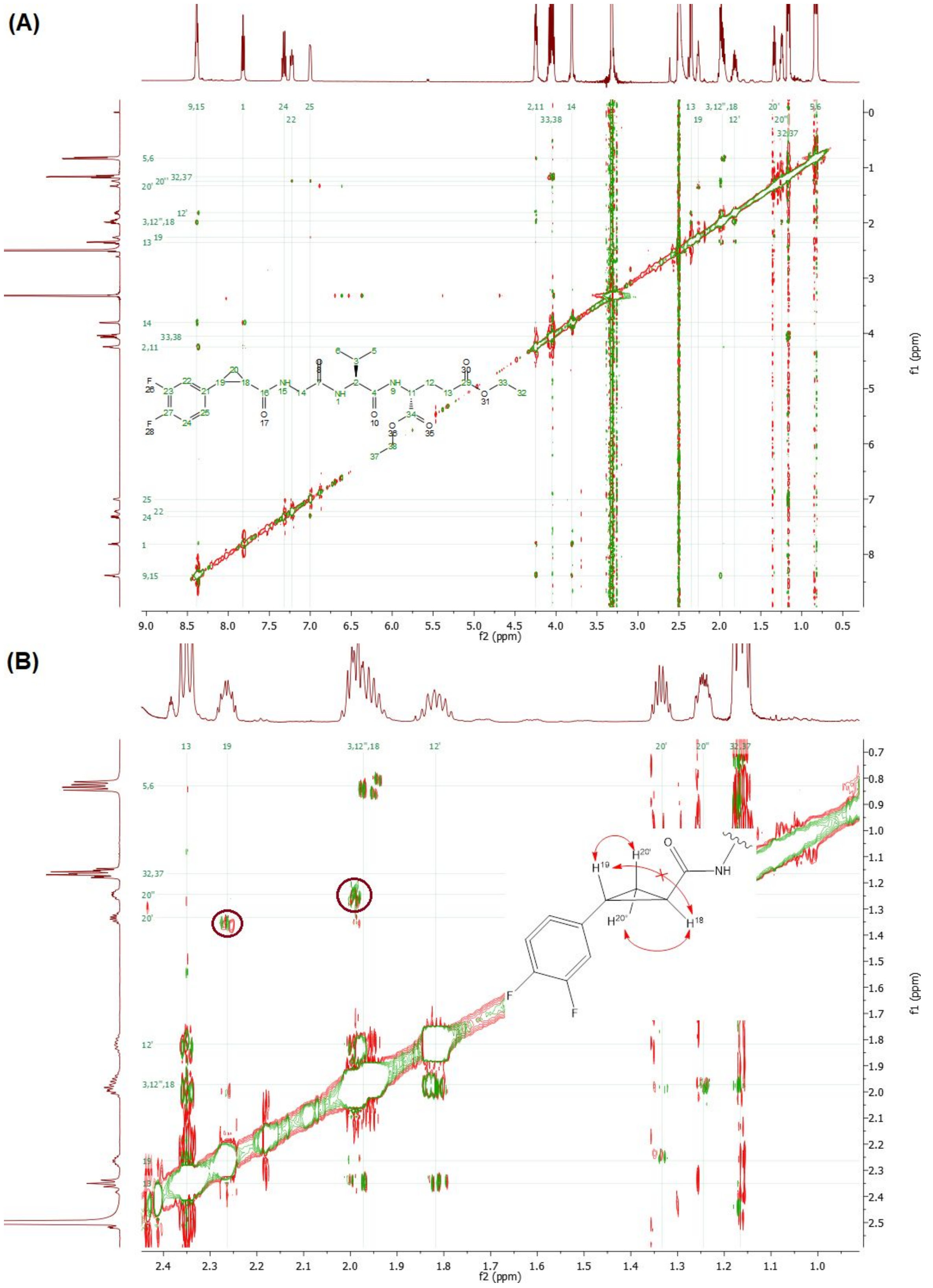

Supporting figure S9. (A) Superimposed 800 MHz ^1H–^1H NOESY spectra of compounds **29a** (in red) and **29b** (in green) in DMSO-d_6. (B) Enlarged region showing the relevant NOE contacts (circled in dark red).

6 Synthesis and characterization of compounds

6.1 General synthetic procedures

6.1.1 General procedure A: TFA-Mediated Acidolysis

The Boc-protected compound was added to an ice-chilled stirred mixture of TFA and DCM (1:5), and the mixture was allowed to warm to room temperature. After 3 h, the solvent was evaporated off *in vacuo*. The residue was washed three times with diethyl ether.

6.1.2 General procedure B: DCC-Mediated Coupling

To an ice-chilled stirred solution of the requisite carboxylic acid (1.0–1.1 eq.) in dry EtOAc, triethylamine (3 eq.), HOBt (1.0–1.1 eq.) and DCC (1.0–1.1 eq.) were added. After stirring for 15 minutes, the requisite amine (1 eq.) and a catalytic amount of DMAP were added, and the mixture was allowed to warm to room temperature. The stirring was continued overnight, after which the mixture was filtered. The filtrate was diluted with EtOAc(40 mL) and washed with 1 M HCl (2 × 20 mL), saturated $NaHCO_3$ solution (2 × 20 mL) and brine (20 mL). The organic layer was dried over anhydrous Na_2SO_4 and concentrated *in vacuo*.

6.1.3 General procedure C: EDC-Mediated Coupling

To an ice-chilled stirred solution of the requisite amine (1.5 eq. when one amide bond was formed; 3 eq. when two amide bonds were formed) and carboxylic acid (1 eq.) in dry DMF, triethylamine (2 eq. when one amide bond was formed; 4 eq. when two amide bonds were formed) was added. After stirring for 15 min, HOBt (1.2 eq. when one amide bond was formed; 2.5 eq. when two amide bonds were formed), EDC (1.2 eq. when one amide bond was formed; 2.5 eq. when two amide bonds were formed) and a catalytic amount of DMAP were added, and the mixture was allowed to warm to room temperature. The stirring was continued overnight, after which the mixture was diluted with EtOAc (30 mL) and washed with 1 M HCl (3 × 10 mL), saturated $NaHCO_3$ solution (2 × 10 mL) and brine (10 mL). The organic layer was dried over anhydrous Na_2SO_4 and concentrated *in vacuo*.

6.2 Diethyl *O*-benzyl-*N*-(tert-butoxycarbonyl)-L-seryl-D-glutamate (4)

Synthesized from *O*-benzyl-*N*-Boc-L-serine (2.74 g, 9.30 mmol) and **2** (2.23 g, 9.30 mmol) using General procedure B. White solid (3.710 g, 83 %). ^{1}H NMR (400 MHz, DMSO-d_6) δ 8.37 (d, *J* = 7.8 Hz, 1H), 7.37 – 7.27 (m, 5H), 6.82 (d, *J* = 8.4 Hz, 1H), 4.49 (s, 2H), 4.35 – 4.21 (m, 2H), 4.12 – 3.97 (m, 4H), 3.65 – 3.49 (m, 2H), 2.34 (t, *J* = 7.4 Hz, 2H), 2.06 – 1.94 (m, 1H), 1.91 – 1.76 (m, 1H), 1.39 (s, 9H), 1.23 – 1.11 (m, 6H).

6.3 Diethyl (tert-butoxycarbonyl)-L-threonyl-D-glutamate (5)

Synthesized from Boc-L-threonine (1.206 g, 5.50 mmol) and **2** (1.199 g, 5.00 mmol) using General procedure B. Yellow oil (991 mg, 45 %). ^{1}H NMR (400 MHz, DMSO-d_6) δ 8.16 (d, J = 7.7 Hz, 1H), 6.29 (d, J = 8.0 Hz, 1H), 4.71 (d, J = 5.6 Hz, 1H), 4.30 – 4.20 (m, 1H), 4.14 – 3.98 (m, 4H), 3.91 – 3.83 (m, 2H), 2.35 (t, J = 7.7 Hz, 2H), 2.05 – 1.92 (m, 1H), 1.89 – 1.76 (m, 1H), 1.38 (s, 9H), 1.22 – 1.13 (m, 6H), 1.04 (d, J = 6.0 Hz, 3H).

6.4 Diethyl *O*-benzyl-*N*-((tert-butoxycarbonyl)glycyl)-L-seryl-D-glutamate (7)

Compound **4** (1.65 g, 3.44 mmol) was deprotected using General procedure A and coupled to Boc-glycine (603 mg, 3.44 mmol) using General procedure B. White solid (1.70 g, 92 %). ^{1}H NMR (400 MHz, DMSO) δ 8.46 (d, *J* = 7.8 Hz, 1H), 7.91 (d, *J* = 8.1 Hz, 1H), 7.39 – 7.24 (m, 5H), 7.04 (t, *J* = 6.1 Hz, 1H), 4.60 (q, *J* = 6.2 Hz, 1H), 4.49 (s, 2H), 4.36 – 4.24 (m, 1H), 4.13 – 3.98 (m, 4H), 3.65 – 3.51 (m, 4H), 2.34 (t, *J* = 7.5 Hz, 2H), 2.05 – 1.94 (m, 1H), 1.91 – 1.74 (m, 1H), 1.38 (s, 9H), 1.22 – 1.12 (m, 6H).

6.5 Diethyl (tert-butoxycarbonyl)glycyl-L-seryl-D-glutamate (8)

A solution of compound **7** (729 mg, 1.36 mmol) in acetic acid (20 mL) was hydrogenated over 10% palladium-on-carbon overnight at room temperature and atmospheric pressure. The catalyst was removed by filtration, the filtrate was concentrated *in vacuo* and co-evaporated twice with diethyl ether to produce the title compound **8** as a white solid (506 mg, 83 %). ^{1}H NMR (400 MHz, MeOD) δ 4.52 – 4.44 (m, 2H), 4.24 – 4.09 (m, 4H), 3.88 – 3.74 (m, 4H), 2.50 – 2.41 (m, 2H), 2.27 – 2.14 (m, 1H), 2.07 – 1.93 (m, 1H), 1.47 (s, 9H), 1.32 – 1.22 (m, 6H).

6.6 Diethyl (tert-butoxycarbonyl)glycyl-L-threonyl-D-glutamate (9)

Compound **5** (908 mg, 2.24 mmol) was deprotected using General procedure A and coupled to Boc-glycine (431 mg, 2.46 mmol) using General procedure B. Orange oil (697 mg, 67 %). ^{1}H NMR (400 MHz, $CDCl_3$) δ 7.58 (d, *J* = 7.9 Hz, 1H), 7.20 (d, *J* = 8.0 Hz, 1H), 5.61 (t, *J* = 5.5 Hz, 1H), 4.56 – 4.46 (m, 1H), 4.45 – 4.36 (m, 2H), 4.25 – 4.07 (m, 6H), 3.88 (d, *J* = 6.0 Hz, 1H), 2.49 – 2.35 (m, 2H), 2.29 – 2.14 (m, 1H), 2.11 – 1.98 (m, 1H), 1.45 (s, 9H), 1.32 – 1.22 (m, 9H).

6.7 Ethyl (*E*)-3-(4-hydroxy-3-methoxyphenyl)acrylate (10)

To an ice-chilled stirring solution of *trans*-ferulic acid (1.942 g, 10.0 mmol) in absolute ethanol (20 mL), thionyl chloride (0.872 mL, 12.0 mmol) was added dropwise. After refluxing the mixture overnight, the solvent was evaporated *in vacuo*. The residue was dissolved in EtOAc (30 mL) and washed with 1 M HCl (15 mL), saturated $NaHCO_3$ solution (2 × 15 mL) and brine (15 mL). The organic layer was dried over anhydrous Na_2SO_4 and concentrated *in vacuo* to produce the title compound **10** as an orange solid (2.012 g, 91 %). ^{1}H NMR (400 MHz, DMSO-d_6) δ 9.61 (s, 1H), 7.54 (d, *J* = 15.9 Hz, 1H), 7.33 (d, *J* = 2.0 Hz, 1H), 7.12 (dd, *J* = 8.2, 2.0 Hz, 1H), 6.79 (d, *J* = 8.2 Hz, 1H), 6.48 (d, *J* = 15.9 Hz, 1H), 4.16 (q, *J* = 7.1 Hz, 2H), 3.81 (s, 3H), 1.25 (t, *J* = 7.1 Hz, 3H).

6.8 Ethyl (*E*)-3-(4-acetoxy-3-methoxyphenyl)acrylate (11)

To an ice-chilled stirring solution of compound **10** (889 mg, 4.0 mmol) in THF (25 mL), triethylamine (0.427 mL, 6.0 mmol) and acetyl chloride (0.835 mL, 6.0 mmol) were added. The mixture was allowed to warm to room temperature. After stirring for 1 h, the solvent was evaporated *in vacuo*. The residue was dissolved in EtOAc (50 mL) and washed with 1 M HCl (25 mL), saturated $NaHCO_3$ solution (25 mL) and brine (25 mL). The organic layer was dried over anhydrous Na_2SO_4 and concentrated *in vacuo* to produce the title compound **11** as a white solid (954 mg, 90 %). ^{1}H NMR (400 MHz, DMSO-d_6) δ 7.64 (d, *J* = 16.0 Hz, 1H), 7.53 (d, *J* = 1.9 Hz, 1H), 7.30 (dd, *J* = 8.3, 1.9 Hz, 1H), 7.13 (d, *J* = 8.1 Hz, 1H), 6.71 (d, *J* = 16.0 Hz, 1H), 4.20 (q, *J* = 7.1 Hz, 2H), 3.82 (s, 3H), 2.26 (s, 3H), 1.27 (t, *J* = 7.1 Hz, 3H).

6.9 Ethyl 2-(3,4-dimethoxyphenyl)cyclopropane-1-carboxylate (12)

To a suspension of sodium hydride (60 % dispersion in mineral oil, 200 mg, 5.0 mmol) in dry DMSO (8 mL), trimethylsulfoxonium iodide (1.100 g, 5.0 mmol) was added under argon. After stirring for 1 h, a solution of compound **11** (528 mg, 2.0 mmol) in dry DMSO (5 mL) was added and the stirring was continued at room temperature under argon overnight. The reaction was quenched with brine (30 mL), after which the mixture was extracted with EtOAc (3 × 20 mL). The combined organic phases were washed with a 1 M NaOH solution (3 × 15 mL), 1 M HCl (15 mL), saturated $NaHCO_3$ solution (15 mL) and brine (15 mL). The organic layer was dried over anhydrous Na_2SO_4 and concentrated *in vacuo* to produce the title compound **12** as a yellow oil (180 mg, 33 %). ^{1}H NMR (400 MHz, DMSO-d_6) δ 6.84 (d, *J* = 8.3 Hz, 1H), 6.74 (d, *J* = 2.1 Hz, 1H), 6.69 (dd, *J* = 8.3, 2.1 Hz, 1H), 4.10 (q, *J* = 7.1 Hz, 2H), 3.74 (s, 3H), 3.71 (s, 3H), 2.42 – 2.32 (m, 1H), 1.94 – 1.85 (m, 1H), 1.46 – 1.31 (m, 2H), 1.20 (t, *J* = 7.1 Hz, 3H).

6.10 2-(3,4-dimethoxyphenyl)cyclopropane-1-carboxylic acid (13)

To a solution of compound **12** (152 mg, 0.550 mmol) in ethanol (5 mL), a 1 M NaOH solution (2.2 mL) was added, and the mixture was stirred overnight at room temperature. The reaction mixture was neutralized with the addition of 1 M HCl and extracted with EtOAc (3 × 20 mL). The combined organic phases were washed with brine (2 × 20 mL), dried over anhydrous Na_2SO_4 and concentrated *in vacuo* to produce the title compound **13** as a yellow solid (110 mg, 90 %). ^{1}H NMR (400 MHz, DMSO-d_6) δ 12.25 (s, 1H), 6.84 (d, *J* = 8.3 Hz, 1H), 6.74 (d, *J* = 2.1 Hz, 1H), 6.67 (dd, *J* = 8.2, 2.1 Hz, 1H), 3.74 (s, 3H), 3.71 (s, 3H), 2.38 – 2.29 (m, 1H), 1.80 – 1.71 (m, 1H), 1.42 – 1.28 (m, 2H).

6.11 Benzyl (*E*)-3-(4-(benzyloxy)-3-methoxyphenyl)acrylate (14)

The procedure for preparation of compound **14** was adapted from a reported procedure.[1]

To a stirring solution of *trans*-ferulic acid (971 mg, 5.0 mmol) in DMF (20 mL), K_2CO_3 (6.91 g, 50 mmol) and benzyl chloride (3.45 mL, 30 mmol) were added. The stirring was continued for 4 hours at 80 °C, before the addition of water (100 mL). The precipitated product was filtered and washed twice with water to produce the title compound **14** as a white solid (1.494 g, 80 %). ^{1}H NMR (400 MHz, DMSO-

d$_6$) δ 7.63 (d, *J* = 15.9 Hz, 1H), 7.48 – 7.29 (m, 11H), 7.24 (dd, *J* = 8.4, 2.0 Hz, 1H), 7.06 (d, *J* = 8.4 Hz, 1H), 6.64 (d, *J* = 16.0 Hz, 1H), 5.21 (s, 2H), 5.13 (s, 2H), 3.81 (s, 3H).

6.12 Benzyl 2-(4-(benzyloxy)-3-methoxyphenyl)cyclopropane-1-carboxylate (15)

To a suspension of sodium hydride (60 % dispersion in mineral oil, 103 mg, 2.58 mmol) in dry DMSO (6 mL), trimethylsulfoxonium iodide (569 mg, 2.58 mmol) was added under argon. After stirring for 1 h, a solution of compound **14** (642 mg, 1.72 mmol) in dry DMSO (20 mL) was added and the stirring was continued at room temperature for 3 h. The reaction was quenched with water (40 mL), after which the mixture was extracted with diethyl ether (3 × 25 mL). The combined organic phases were washed with water (2 × 30 mL) and brine (30 mL), dried over anhydrous Na_2SO_4 and concentrated *in vacuo*. Purification by flash chromatography (10% EtOAc in hexanes) produced the title compound **15** as a colorless oil (233 mg, 35 %). ^{1}H NMR (400 MHz, DMSO-d$_6$) δ 7.45 – 7.33 (m, 8H), 7.28 – 7.17 (m, 2H), 6.90 (d, *J* = 8.3 Hz, 1H), 6.78 (d, *J* = 2.1 Hz, 1H), 6.67 (dd, *J* = 8.3, 2.1 Hz, 1H), 5.13 (d, *J* = 2.9 Hz, 2H), 5.03 (s, 2H), 3.76 (s, 3H), 2.47 – 2.38 (m, 1H), 2.03 – 1.94 (m, 1H), 1.50 – 1.34 (m, 2H).

6.13 4-(4-hydroxy-3-methoxyphenyl)butanoic acid (16)

A solution of compound **15** (70 mg, 0.180 mmol) in ethanol (20 mL) was hydrogenated over 10% palladium-on-carbon overnight at room temperature and under atmospheric pressure. The catalyst was removed by filtration and the filtrate concentrated *in vacuo* to produce the title compound **16** as a white solid (38 mg, 100 %). ^{1}H NMR (400 MHz, DMSO-d$_6$) δ 6.71 (d, J = 2.0 Hz, 1H), 6.66 (d, J = 7.9 Hz, 1H), 6.54 (dd, J = 7.9, 2.0 Hz, 1H), 3.73 (s, 3H), 2.44 (t, J = 7.4 Hz, 2H), 2.09 (t, J = 7.4 Hz, 2H), 1.71 (p, J = 7.5 Hz, 2H).

6.14 2-(4-hydroxy-3-methoxyphenyl)cyclopropane-1-carboxylic acid (17)

To a solution of palladium (II) acetate (15 mg, 0.069 mmol) in dry dichloromethane (10 mL) were added triethylamine (19 µL, 0.138 mmol) and triethylsilane (220 µL, 1.38 mmol). The resulting black suspension was stirred for 15 minutes, after which a solution of compound **15** (268 mg, 0.69 mmol) in dry DCM (10 mL) was added. The resulting mixture was stirred under argon atmosphere for 18 h, diluted with DCM (20 mL) and extracted with a 1 M NaOH solution (2 × 15 mL). Combined aqueous

phases were washed with diethyl ether (3 × 10 mL), acidified to pH = 1 with 1 M HCl and extracted with DCM (3 × 15 mL). Combined organic phases were washed with water (20 mL) and brine (20 mL), dried over anhydrous Na_2SO_4 and concentrated *in vacuo* to produce the title compound **17** as a white solid (51 mg, yield: 35%). ^{1}H NMR (400 MHz, DMSO-d_6) δ 12.21 (s, 1H), 8.78 (s, 1H), 6.70 (d, J = 2.1 Hz, 1H), 6.65 (d, J = 8.1 Hz, 1H), 6.54 (dd, J = 8.1, 2.1 Hz, 1H), 3.75 (s, 3H), 2.33 – 2.23 (m, 1H), 1.75 – 1.66 (m, 1H), 1.38 – 1.30 (m, 1H), 1.31 – 1.23 (m, 1H).

6.15 (*E*)-3-(4-((tert-butoxycarbonyl)amino)phenyl)acrylic acid (22)

To an ice-chilled stirring solution of 4-aminocinnamic acid (998 mg, 5.0 mmol) in water (4 mL) and 1 M NaOH (6.5 mL), a solution of di-*tert*-butyl dicarbonate (1.419 g, 6.5 mmol) in dioxane (20 mL) was added. The mixture was allowed to warm to room temperature and the stirring was continued overnight. Dioxane was evaporated *in vacuo*, after which the mixture was washed with diethyl ether (10 mL). The aqueous phase was acidified to pH = 1 with 1 M HCl and extracted with EtOAc (3 × 20 mL). The combined organic phases were dried over anhydrous Na_2SO_4 and concentrated *in vacuo* to produce the title compound **22** as an orange solid (1.062 g, 81 %). ^{1}H NMR (400 MHz, DMSO-d_6) δ 12.17 (s, 1H), 9.59 (s, 1H), 7.62 – 7.55 (m, 2H), 7.53 – 7.46 (m, 3H), 6.38 (d, *J* = 16.0 Hz, 1H), 1.48 (s, 9H).

6.16 5-benzyl 1-ethyl (tert-butoxycarbonyl)-D-glutamate (33)

Compound **33** was prepared from Boc-D-glutamic acid 5-benzyl ester according to a reported procedure.[2]

6.17 5-benzyl 1-ethyl (tert-butoxycarbonyl)-L-valyl-D-glutamate (34)

Compound **33** (1.827 g, 5 mmol) was deprotected using General procedure A and coupled to Boc-L-valine (1.194 g, 5.5 mmol) using General procedure B. White solid (1.908 g, 82 %). 1H NMR (400 MHz, DMSO-d6) δ 8.22 (d, J = 7.5 Hz, 1H), 7.42 – 7.28 (m, 5H), 6.61 (d, J = 8.9 Hz, 1H), 5.08 (s, 2H), 4.28 – 4.18 (m, 1H), 4.13 – 4.00 (m, 2H), 3.79 (t, J = 8.6 Hz, 1H), 2.43 (t, J = 7.6 Hz, 2H), 2.09 – 1.96 (m, 1H), 1.93 – 1.81 (m, 1H), 1.79 – 1.63 (m, 1H), 1.36 (s, 9H), 1.16 (t, J = 7.1 Hz, 3H), 0.83 (t, J = 6.2 Hz, 6H).

6.18 5-benzyl 1-ethyl (tert-butoxycarbonyl)glycyl-L-valyl-D-glutamate (35)

Compound **34** (1.858 g, 4.0 mmol) was deprotected using General procedure A and coupled to Boc-glycine (770 mg, 4.4 mmol) using General procedure B. White solid (1.395 g, 67 %). ^{1}H NMR (400 MHz, DMSO-d$_6$) δ 8.45 (d, *J* = 7.5 Hz, 1H), 7.60 (d, *J* = 9.1 Hz, 1H), 7.42 – 7.29 (m, 5H), 7.05 (t, *J* = 6.1 Hz, 1H), 5.09 (s, 2H), 4.32 – 4.21 (m, 2H), 4.12 – 4.03 (m, 2H), 3.58 (d, *J* = 6.0 Hz, 2H), 2.45 (t, *J* = 7.5 Hz, 2H), 2.09 – 1.70 (m, 3H), 1.38 (s, 9H), 1.17 (t, *J* = 7.1 Hz, 3H), 0.86 – 0.77 (m, 6H).

6.19 (9*S*,12*R*)-12-(ethoxycarbonyl)-9-isopropyl-2,2-dimethyl-4,7,10-trioxo-3-oxa-5,8,11-triazapentadecan-15-oic acid (36)

A solution of compound **35** (450 mg, 0.863 mmol) in ethanol (20 mL) was hydrogenated over 10% palladium-on-carbon for 2 hours at room temperature and atmospheric pressure. The catalyst was removed by filtration and the filtrate concentrated *in vacuo* to produce the title compound **36** as a white solid (370 mg, 99 %). ^{1}H NMR (400 MHz, DMSO-d$_6$) δ 12.20 (s, 1H), 8.42 (d, *J* = 7.5 Hz, 1H), 7.57 (d, *J* = 8.9 Hz, 1H), 7.04 (t, *J* = 5.7 Hz, 1H), 4.30 – 4.20 (m, 2H), 4.13 – 4.03 (m, 2H), 3.57 (d, *J* = 6.1 Hz, 2H), 2.28 (t, *J* = 7.4 Hz, 2H), 2.02 – 1.89 (m, 2H), 1.87 – 1.64 (m, 1H), 1.38 (s, 9H), 1.18 (t, *J* = 7.1 Hz, 3H), 0.87 – 0.77 (m, 6H).

6.20 (*S*)-6-((((9H-fluoren-9-yl)methoxy)carbonyl)amino)-1-methoxy-1-oxohexan-2-aminium chloride (37)

To an ice-chilled stirring solution of Boc-lysine(Fmoc)-OH (1.516 g, 3.236 mmol) in methanol (20 mL), thionyl chloride (0.47 mL, 6.472 mmol) was added dropwise. The mixture was allowed to warm to room temperature, and the stirring was continued overnight. The solvent was evaporated *in vacuo* and the residue was washed twice with diethyl ether to produce the title compound **37** as a white solid (1.438 g, 100 %). ^{1}H NMR (400 MHz, DMSO-d$_6$) δ 8.61 (s, 3H), 7.90 (d, *J* = 7.8 Hz, 2H), 7.69 (d, *J* = 7.3 Hz, 2H), 7.42 (t, *J* = 7.5 Hz, 2H), 7.36 – 7.28 (m, 3H), 4.30 (d, *J* = 6.9 Hz, 2H), 4.21 (t, *J* = 6.8 Hz, 1H), 3.98 (t, *J* = 6.2 Hz, 1H), 3.73 (s, 3H), 2.97 (q, *J* = 6.4 Hz, 2H), 1.80 (q, *J* = 7.1 Hz, 2H), 1.46 – 1.34 (m, 3H), 1.32 – 1.25 (m, 1H).

6.21 Methyl N^6-(((9H-fluoren-9-yl)methoxy)carbonyl)-N^2-((*R*)-4-((*S*)-2-(2-((tert-butoxycarbonyl)amino)acetamido)-3-methylbutanamido)-5-ethoxy-5-oxopentanoyl)-L-lysinate (38)

To an ice-chilled stirring solution of compound **36** (542 mg, 1.233 mmol) and compound **37** (620 mg, 1.480 mmol) in DMF (5 mL), DIPEA (1.074 mL, 6.165 mmol) and COMU (634 mg, 1.480 mmol) were added. The mixture was allowed to warm to room temperature, after which the stirring was continued for 3 h. The mixture was diluted with EtOAc (80 mL) and washed with 1 M HCl (2 × 30 mL), saturated $NaHCO_3$ solution (3 × 30 mL) and brine (30 mL). The organic layer was dried over anhydrous Na_2SO_4 and concentrated *in vacuo* to produce the title compound **38** (905 mg, 62 %). ^1H NMR (400 MHz, DMSO-d_6) δ 8.44 (d, *J* = 7.2 Hz, 1H), 8.22 (d, *J* = 7.5 Hz, 1H), 7.93 – 7.86 (m, 2H), 7.68 (d, *J* = 7.5 Hz, 2H), 7.57 (d, *J* = 9.1 Hz, 1H), 7.46 – 7.37 (m, 2H), 7.36 – 7.24 (m, 3H), 7.04 (t, *J* = 6.1 Hz, 1H), 4.34 – 4.25 (m, 3H), 4.24 – 4.14 (m, 3H), 4.12 – 4.02 (m, 2H), 3.73 – 3.68 (m, 2H), 3.59 (s, 3H), 3.01 – 2.90 (m, 2H), 2.20 (t, *J* = 7.2 Hz, 2H), 1.99 – 1.86 (m, 2H), 1.86 – 1.73 (m, 2H), 1.69 – 1.53 (m, 1H), 1.46 – 1.24 (m, 13H), 1.17 (t, *J* = 7.1 Hz, 3H), 0.87 – 0.78 (m, 6H).

6.22 2,5-dioxopyrrolidin-1-yl (*E*)-3-(4-hydroxy-3-methoxyphenyl)acrylate (39)

Compound **39** was prepared from *trans*-ferulic acid according to a reported procedure.[3]

^1H NMR (400 MHz, DMSO-d_6) δ 9.91 (s, 1H), 7.84 (d, *J* = 15.9 Hz, 1H), 7.49 (d, *J* = 2.0 Hz, 1H), 7.27 (dd, *J* = 8.3, 2.0 Hz, 1H), 6.85 – 6.77 (m, 2H), 3.84 (s, 3H), 2.86 (d, *J* = 7.5 Hz, 4H).

6.23 Methyl N^6-(((9H-fluoren-9-yl)methoxy)carbonyl)-N^2-((*R*)-5-ethoxy-4-((*S*)-2-(2-((*E*)-3-(4-hydroxy-3-methoxyphenyl)acrylamido)acetamido)-3-methylbutanamido)-5-oxopentanoyl)-L-lysinate (40)

Compound **38** (227 mg, 0.285 mmol) was deprotected using General procedure A. It was then dissolved in THF (10 mL) and water (10 mL), before the addition of $NaHCO_3$ (96 mg, 1.140 mmol) and **39** (100 mg, 0.342 mmol). The stirring was continued overnight at room temperature in the dark. THF was evaporated *in vacuo*, after which the mixture was diluted with EtOAc (40 mL) and washed with 1 M HCl (2 × 20 mL), saturated $NaHCO_3$ solution (2 × 20 mL) and brine (20 mL). The organic layer was

dried over anhydrous Na_2SO_4 and concentrated *in vacuo*. Purification by flash chromatography (6% MeOH in DCM) produced the title compound **40** as a white solid (78 mg, 31 %). ^{1}H NMR (400 MHz, $CDCl_3$) δ 7.80 – 7.67 (m, 3H), 7.62 – 7.43 (m, 4H), 7.43 – 7.35 (m, 2H), 7.35 – 7.24 (m, 3H), 7.12 (d, *J* = 8.2 Hz, 1H), 7.04 – 6.94 (m, 3H), 6.84 (t, *J* = 7.0 Hz, 1H), 6.27 (d, *J* = 15.6 Hz, 1H), 6.06 (s, 1H), 5.36 (t, *J* = 6.0 Hz, 1H), 4.52 – 4.37 (m, 3H), 4.35 – 4.06 (m, 5H), 3.98 – 3.88 (m, 1H), 3.85 (s, 3H), 3.72 (s, 3H), 3.30 – 3.00 (m, 2H), 2.33 – 2.10 (m, 5H), 1.78 – 1.68 (m, 2H), 1.53 – 1.33 (m, 4H), 1.28 – 1.18 (m, 3H), 1.03 – 0.91 (m, 6H).

6.24 Di-tert-butyl (tert-butoxycarbonyl)-D-glutamate (47)

To a stirring solution of Boc-D-glutamic acid 1-tert-butyl ester (303 mg, 1.00 mmol) in DCM (5 mL), a solution of tert-butyl 2,2,2-trichloroacetimidate (0.437 mg, 2.00 mmol) in cyclohexane (5 mL) was added. Boron trifluoride diethyl etherate (41 µL, 0.33 mmol) was added and the stirring was continued overnight at room temperature. Solid $NaHCO_3$ was added, before the mixture was filtered. The filtrate was concentrated *in vacuo*, after which the residue was dissolved in EtOAc (20 mL) and washed with a saturated $NaHCO_3$ solution (2 × 10 mL). The organic layer was dried over anhydrous Na_2SO_4 and concentrated *in vacuo*. Purification by flash chromatography (20% EtOAc in hexanes) produced the title compound **47** as a white solid (197 mg, 55 %). ^{1}H NMR (400 MHz, DMSO-d_6) δ 7.12 (d, J = 8.0 Hz, 1H), 3.85 – 3.79 (m, 1H), 2.29 – 2.19 (m, 2H), 1.91 – 1.82 (m, 1H), 1.74 – 1.64 (m, 1H), 1.39 – 1.34 (m, 27H). HRMS calcd for $C_{14}H_{33}NO_6Na$ m/z: 382.2206 $(M + Na)^+$, found 382.2216.

6.25 Tert-butyl (*R*)-(1,5-bis(ethylamino)-1,5-dioxopentan-2-yl)carbamate (48)

Synthesized from Boc-D-glutamic acid (432 mg, 1.75 mmol) and ethylammonium chloride (428 mg, 5.25 mmol) using General procedure C. White solid (108 mg, 20 %). ^{1}H NMR (400 MHz, DMSO-d_6) δ 7.79 – 7.77 (m, 2H), 6.77 (d, J = 8.0 Hz, 1H), 3.84 – 3.78 (m, 1H), 3.11 – 3.00 (m, 4H), 2.07 – 2.02 (m, 2H), 1.84 – 1.75 (m, 1H), 1.72 – 1.61 (m, 1H), 1.38 (s, 9H), 1.01 – 0.96 (m, 6H).

6.26 Tert-butyl (*R*)-(1,5-bis(butylamino)-1,5-dioxopentan-2-yl)carbamate (49)

Synthesized from Boc-D-glutamic acid (247 mg, 1.00 mmol) and butylamine (0.295 mL, 3.00 mmol) using General procedure C. Orange solid (356 mg, 99 %). ^{1}H NMR (400 MHz, DMSO-d_6) δ 7.77 – 7.72

(m, 2H), 6.79 (d, J = 8.0 Hz, 1H), 3.85 – 3.80 (m, 1H), 3.11 – 2.99 (m, 4H), 2.09 – 2.04 (m, 2H), 1.85 – 1.76 (m, 1H), 1.72 – 1.62 (m, 1H), 1.41 – 1.32 (m, 13H), 1.31 – 1.24 (m, 4H), 0.88 – 0.85 (m, J = 7.6 Hz, 6H). HRMS calcd for $C_{18}H_{36}N_3O_4$ m/z: 358.2706 $(M + H)^+$, found 358.2699.

6.27 Tert-butyl *N*²-(tert-butoxycarbonyl)-*N*⁵-butyl-D-glutaminate (50)

Synthesized from Boc-D-glutamic acid 1-tert-butyl ester (100 mg, 0.330 mmol) and butylamine (49 µL, 0.495 mmol) using General procedure C. Colourless oil (96 mg, 81 %). ^1H NMR (400 MHz, DMSO-d_6) δ 7.75 (t, J = 5.6 Hz, 1H), 7.11 (d, J = 7.6 Hz, 1H), 3.78 – 3.72 (m, 1H), 3.03 – 2.98 (m, 2H), 2.11 (t, J = 8.0 Hz, 2H), 1.91 – 1.82 (m, 1H), 1.74 – 1.64 (m, 1H), 1.41 – 1.34 (m, 20H), 1.28 – 1.23 (m, 2H), 0.86 (t, J = 7.6 Hz, 3H). HRMS calcd for $C_{18}H_{35}N_2O_5$ m/z: 359.2546 $(M + H)^+$, found 359.2554.

6.28 Tert-butyl *N*²-(tert-butoxycarbonyl)-*N*⁵-methoxy-D-glutaminate (51)

Synthesized from Boc-D-glutamic acid 1-tert-butyl ester (100 mg, 0.330 mmol) and methoxyamine hydrochloride (41 mg, 0.495 mmol) using General procedure C. Colourless oil (78 mg, 71 %). ^1H NMR (400 MHz, DMSO-d_6) δ 10.97 (s, 1H), 7.13 (d, J = 8.0 Hz, 1H), 3.79 – 3.74 (m, 1H), 3.56 (s, 3H), 2.01 (t, J = 7.6 Hz, 2H), 1.92 – 1.84 (m, 1H), 1.75 – 1.65 (m, 1H), 1.41 – 1.34 (m, 18H). HRMS calcd for $C_{15}H_{28}N_2O_6Na$ m/z: 355.1845 $(M + Na)^+$, found 355.1857.

6.29 Tert-butyl *N*²-(tert-butoxycarbonyl)-*N*⁵-methoxy-*N*⁵-methyl-D-glutaminate (52)

Synthesized from Boc-D-glutamic acid 1-tert-butyl ester (100 mg, 0.330 mmol) and *N*-methoxy-*N*-methylamine hydrochloride (48 mg, 0.495 mmol) using General procedure C. Yellow oil (107 mg, 94 %). ^1H NMR (400 MHz, DMSO-d_6) δ 7.16 (d, J = 7.6 Hz, 1H), 3.84 – 3.78 (m, 1H), 3.63 (s, 3H), 3.07 (s, 3H), 2.47 – 2.43 (m, 2H), 1.93 – 1.84 (m, 1H), 1.78 – 1.67 (m, 1H), 1.42 – 1.35 (m, 18H). HRMS calcd for $C_{16}H_{31}N_2O_6$ m/z: 347.2182 $(M + H)^+$, found 247.2190.

6.30 Tert-butyl (*R*)-2-((tert-butoxycarbonyl)amino)-5-oxo-5-(pyrrolidin-1-yl)pentanoate (53)

Synthesized from Boc-D-glutamic acid 1-tert-butyl ester (100 mg, 0.330 mmol) and pyrrolidine (41 µL, 0.495 mmol) using General procedure C. Off-white solid (106 mg, 90 %). ^1H NMR (400 MHz, DMSO-d_6) δ 7.16 (d, J = 7.6 Hz, 1H), 3.83 – 3.77 (m, 1H), 3.34 (t, J = 6.8 Hz, 2H), 3.26 (t, J = 6.8 Hz, 2H), 2.32 –

2.25 (m, 2H), 1.93 – 1.82 (m, 3H), 1.78 – 1.73 (m, 3H), 1.41 – 1.34 (m, 18H). HRMS calcd for $C_{18}H_{33}N_2O_5$ m/z: 357.2389 $(M + H)^+$, found 357.2391.

6.31 Tert-butyl (*R*)-4-((tert-butoxycarbonyl)amino)-5-(methoxy(methyl)amino)-5-oxopentanoate (54)

Synthesized from Boc-D-glutamic acid 1-tert-butyl ester (100 mg, 0.330 mmol) and *N*-methoxy-*N*-methylamine hydrochloride (48 mg, 0.495 mmol) using General procedure C. Colourless oil (79 mg, 69 %). ^{1}H NMR (400 MHz, DMSO-d_6) δ 7.04 (d, J = 8.0 Hz, 1H), 4.45 – 4.38 (m, 1H), 3.71 (s, 3H), 3.10 (s, 3H), 2.23 (t, J = 7.2 Hz, 2H), 1.83 – 1.74 (m, 1H), 1.69 – 1.60 (m, 1H), 1.39 (s, 9H), 1.37 (s, 9H). HRMS calcd for $C_{16}H_{31}N_2O_6Na$ m/z: 369.2002 $(M + Na)^+$, found 369.1996.

6.32 Tert-butyl (*R*)-4-((tert-butoxycarbonyl)amino)-5-oxo-5-(pyrrolidin-1-yl)pentanoate (55)

Synthesized from Boc-D-glutamic acid 1-tert-butyl ester (100 mg, 0.330 mmol) and pyrrolidine (41 µL, 0.495 mmol) using General procedure C. Yellow oil (114 mg, 97 %). ^{1}H NMR (400 MHz, DMSO-d_6) δ 6.94 (d, J = 8.4 Hz, 1H), 4.22 – 4.17 (m, 1H), 3.53 – 3.44 (m, 2H), 3.34 – 3.22 (m, 2H), 2.24 (t, J = 7.2 Hz, 2H), 1.92 – 1.86 (m, 2H), 1.80 – 1.75 (m, 3H), 1.68 – 1.59 (m, 1H), 1.40 – 1.37 (m, 18H). HRMS calcd for $C_{18}H_{33}N_2O_5$ m/z: 357.2389 $(M + H)^+$, found 357.2394.

6.33 Dicyclopentyl (tert-butoxycarbonyl)-D-glutamate (65)

To an ice-chilled stirring solution of *N*-Boc-D-glutamic acid (495 mg, 2.0 mmol) in DCM (15 ml), cyclopentanol (0.727 mL, 8 mmol), EDC (844 mg, 4.4 mmol), and DMAP (65 mg, 0.53 mmol) were added. The mixture was allowed to warm to room temperature, and the stirring was continued overnight. The solvent was evaporated off *in vacuo*. The residue was dissolved in EtOAc (25 mL) and washed with 1 M HCl (2 × 25 mL), saturated $NaHCO_3$ solution (2 × 25 mL) and brine (25 mL). The organic layer was dried over anhydrous Na_2SO_4 and concentrated *in vacuo* to produce the title compound **SG22** as a colorless oil (625 mg, 82 %). ^{1}H NMR (400 MHz, $CDCl_3$) δ 5.25 – 5.13 (m, 2H), 5.09 (d, J = 8.4 Hz, 1H), 4.29 – 4.20 (m, 1H), 2.44 – 2.25 (m, 2H), 2.20 – 2.06 (m, 1H), 1.95 – 1.82 (m, 5H), 1.79 – 1.64 (m, 8H), 1.64 – 1.53 (m, 4H), 1.44 (s, 9H).

6.34 Dicyclopentyl (tert-butoxycarbonyl)-L-valyl-D-glutamate (66)

Compound **65** (591 mg, 1.54 mmol) was deprotected using General procedure A and coupled to Boc-L-valine (368 mg, 1.70 mmol) using General procedure B. Yellow oil (494 mg, 67 %). ^{1}H NMR (400 MHz, $CDCl_3$) δ 6.69 (d, J = 7.7 Hz, 1H), 5.25 – 5.11 (m, 2H), 5.00 (s, 1H), 4.58 – 4.49 (m, 1H), 3.99 (s, 1H), 2.43 – 2.25 (m, 2H), 2.23 – 2.10 (m, 2H), 2.02 – 1.89 (m, 1H), 1.88 – 1.78 (m, 4H), 1.75 – 1.64 (m, 8H), 1.63 – 1.54 (m, 4H), 1.45 (s, 9H), 0.97 (d, J = 6.8 Hz, 3H), 0.91 (d, J = 6.9 Hz, 3H).

6.35 Dicyclopentyl (tert-butoxycarbonyl)glycyl-L-valyl-D-glutamate (67)

Compound **66** (462 mg, 0.960 mmol) was deprotected using General procedure A and coupled to Boc-glycine (184 mg, 1.05 mmol) using General procedure B. White solid (330 mg, 64 %). ^{1}H NMR (400 MHz, $CDCl_3$) δ 6.99 (d, J = 7.1 Hz, 1H), 6.61 (d, J = 8.8 Hz, 1H), 5.27 – 5.09 (m, 3H), 4.46 (td, J = 7.8, 4.7 Hz, 1H), 4.36 (dd, J = 8.7, 5.3 Hz, 1H), 3.90 (dd, J = 16.9, 6.2 Hz, 1H), 3.79 (dd, J = 16.8, 5.4 Hz, 1H), 2.35 (q, J = 7.0 Hz, 2H), 2.31 – 2.22 (m, 1H), 2.20 – 2.07 (m, 1H), 2.06 – 1.92 (m, 1H), 1.90 – 1.79 (m, 4H), 1.77 – 1.54 (m, 12H), 1.46 (s, 9H), 0.97 (d, J = 6.8 Hz, 3H), 0.93 (d, J = 6.8 Hz, 3H).

6.36 Dioctadecyl D-glutamate (69)

Compound **69** was prepared from D-glutamic acid according to a reported procedure.[4]

^{1}H NMR (400 MHz, $CDCl_3$) δ 4.11 (t, *J* = 6.8 Hz, 2H), 4.07 (t, *J* = 6.8 Hz, 2H), 3.52 – 3.40 (m, 1H), 2.46 (t, *J* = 7.6 Hz, 2H), 2.16 – 2.00 (m, 1H), 1.91 – 1.77 (m, 1H), 1.68 – 1.57 (m, 4H), 1.37 – 1.17 (m, 60H), 0.88 (t, *J* = 6.8 Hz, 6H).

6.37 Dioctadecyl (tert-butoxycarbonyl)-L-valyl-D-glutamate (70)

To an ice-chilled stirring solution of compound **69** (727 mg, 1.12 mmol) in DCM (25 mL), DIPEA (0.583 mL, 3.35 mmol), Boc-L-valine (291 mg, 1.34 mmol), HOBt (181 mg, 1.34 mmol), a catalytic amount of DMAP, and EDC (257 mg, 1.34 mmol) were added. The mixture was allowed to warm to room temperature, and the stirring was continued overnight. The mixture was diluted with DCM (25 mL) and washed with 1 M HCl (2 × 20 mL), saturated $NaHCO_3$ solution (2 × 20 mL) and brine (20 mL). The organic layer was dried over anhydrous Na_2SO_4 and concentrated *in vacuo* to produce the title compound **70** as a white solid (850 mg, 90 %). ^{1}H NMR (400 MHz, $CDCl_3$) δ 6.67 (d, *J* = 7.7 Hz, 1H),

5.08 – 4.88 (m, 1H), 4.66 – 4.53 (m, 1H), 4.12 (t, *J* = 6.8 Hz, 2H), 4.05 (t, *J* = 6.8 Hz, 2H), 4.00 (s, 1H), 2.48 – 2.28 (m, 2H), 2.27 – 2.14 (m, 2H), 2.06 – 1.93 (m, 1H), 1.67 – 1.56 (m, 4H), 1.45 (s, 9H), 1.25 (s, 60H), 0.98 (d, *J* = 6.8 Hz, 3H), 0.93 – 0.84 (m, 9H).

6.38 Dioctadecyl (tert-butoxycarbonyl)glycyl-L-valyl-D-glutamate (71)

Compound **70** (802 mg, 0.940 mmol) was deprotected using General procedure A. It was then dissolved in DCM (25 mL), before the addition of DIPEA (0.82 mL, 4.71 mmol), Boc-glycine (198 mg, 1.13 mmol), HOBt (153 mg, 1.13 mmol), a catalytic amount of DMAP, and EDC (217 mg, 1.13 mmol) at 0 °C. The mixture was allowed to warm to room temperature, and the stirring was continued overnight. The mixture was diluted with DCM (25 mL) and washed with 1 M HCl (2 × 25 mL), saturated $NaHCO_3$ solution (2 × 25 mL) and brine (25 mL). The organic layer was dried over anhydrous Na_2SO_4 and concentrated *in vacuo* to produce the title compound **71** as a white solid (804 mg, 94 %). ^1H NMR (400 MHz, $CDCl_3$) δ 7.00 (d, *J* = 7.3 Hz, 1H), 6.61 (d, *J* = 8.7 Hz, 1H), 5.25 (s, 1H), 4.56 – 4.48 (m, 1H), 4.37 (dd, *J* = 8.7, 5.2 Hz, 1H), 4.11 (t, *J* = 6.9 Hz, 2H), 4.06 (t, *J* = 6.8 Hz, 2H), 3.94 – 3.79 (m, 2H), 2.46 – 2.33 (m, 2H), 2.32 – 2.12 (m, 2H), 2.09 – 1.97 (m, 1H), 1.65 – 1.57 (m, 4H), 1.46 (s, 9H), 1.25 (s, 60H), 0.97 (d, *J* = 6.9 Hz, 3H), 0.92 (d, *J* = 6.9 Hz, 3H), 0.91 – 0.84 (m, 6H).

7 ^{1}H and ^{13}C NMR spectra of representative tested compounds

Compound **20**: ^{1}H, 400 MHz, MeOD

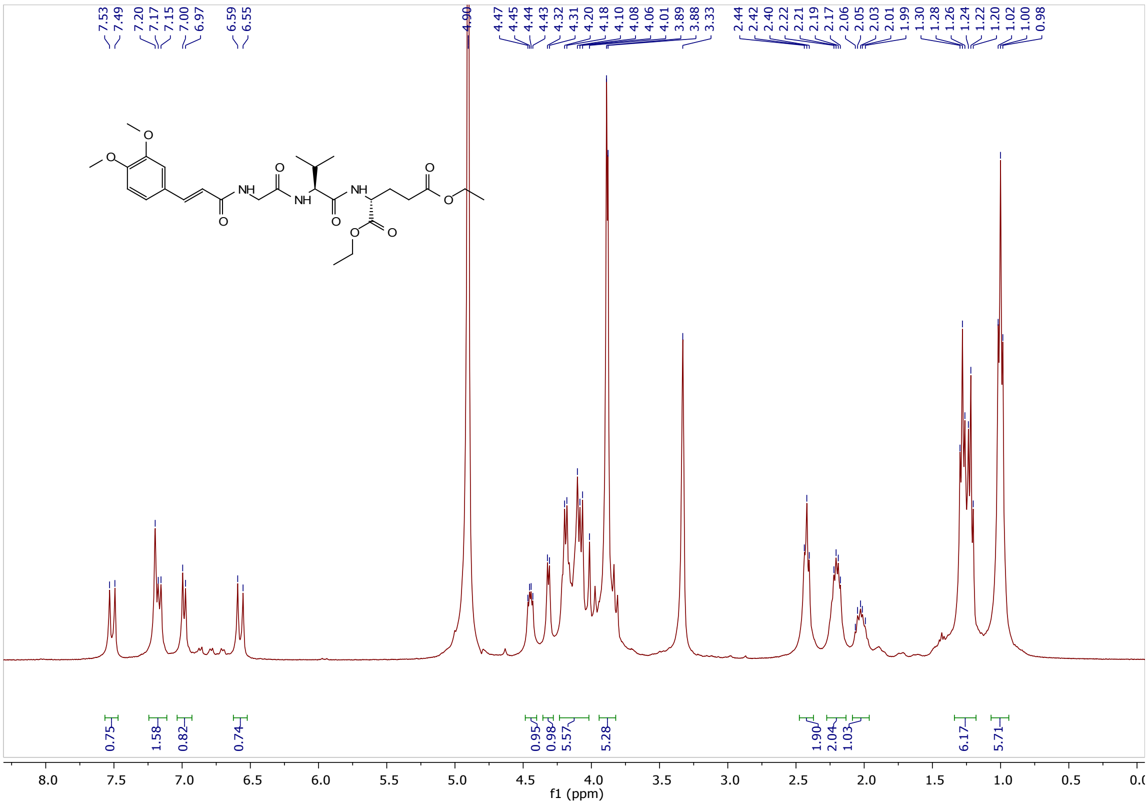

Compound **20**: ^{13}C, 100 MHz, MeOD

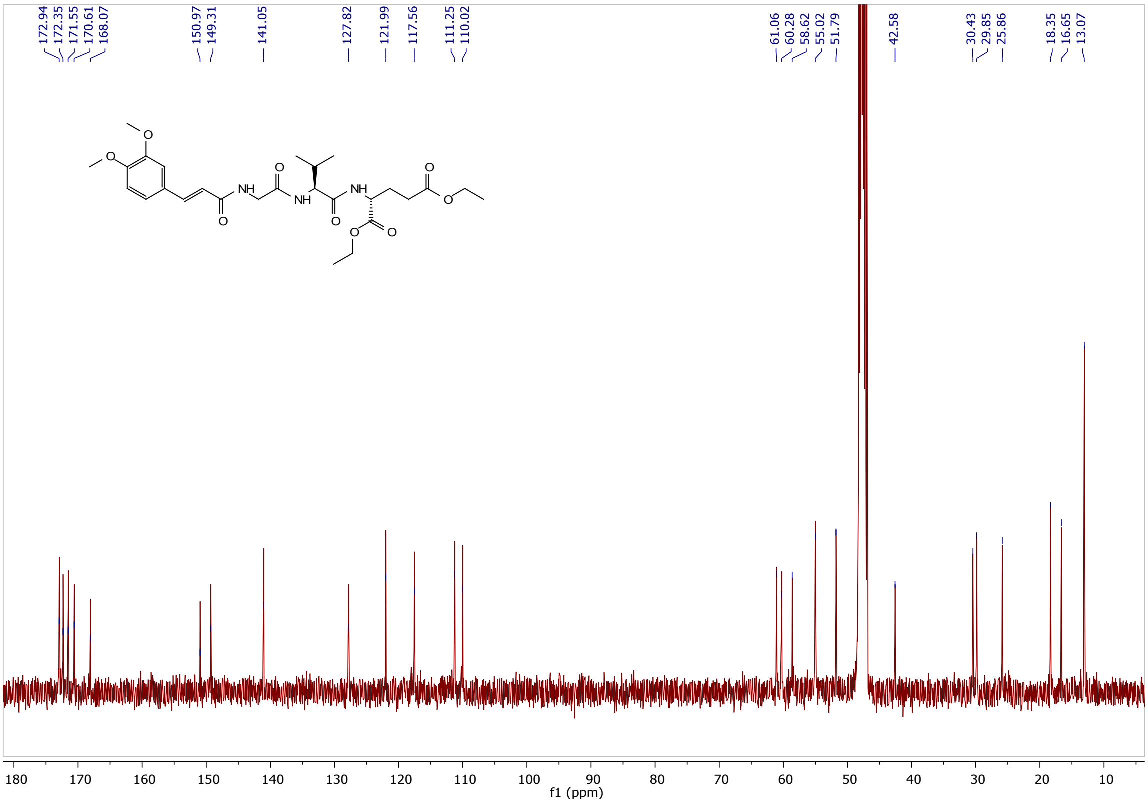

Compound **21**: ^{1}H, 400 MHz, MeOD

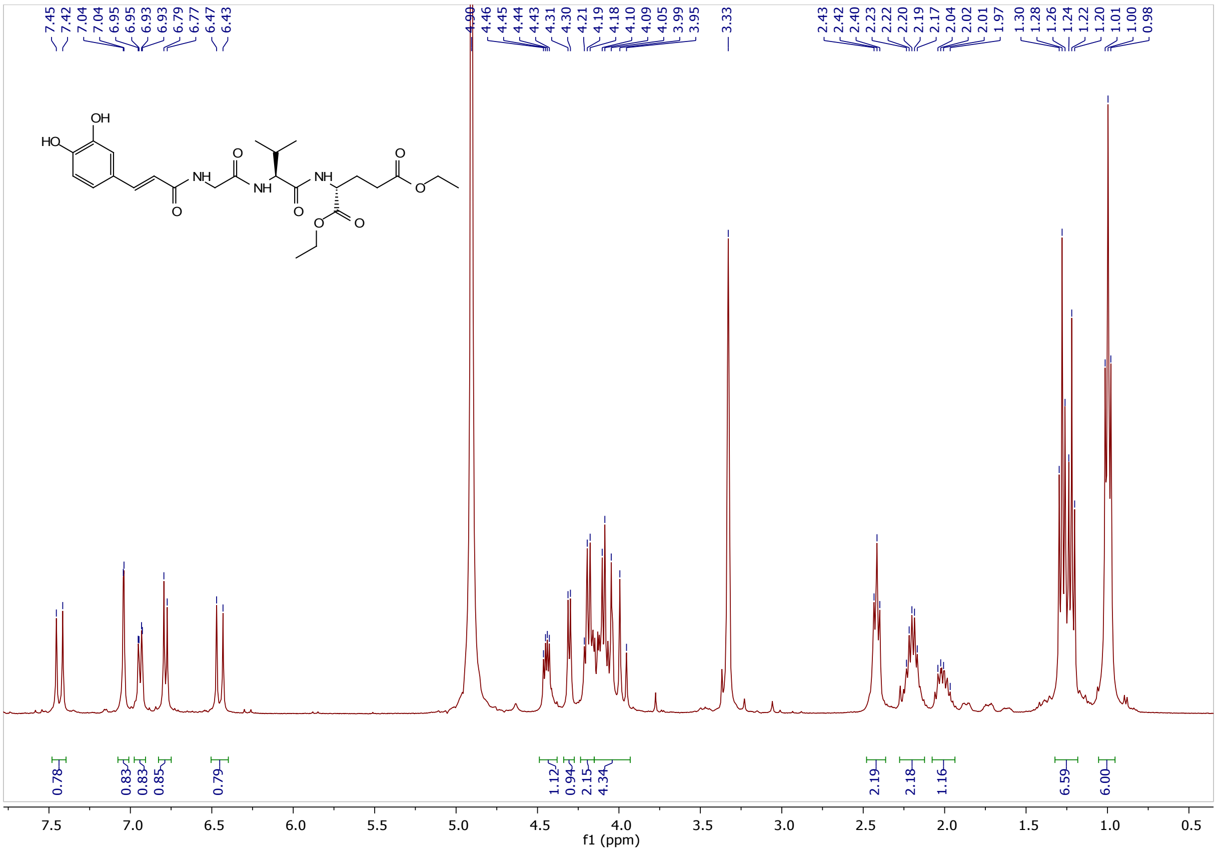

Compound **21**: ^{13}C, 100 MHz, MeOD

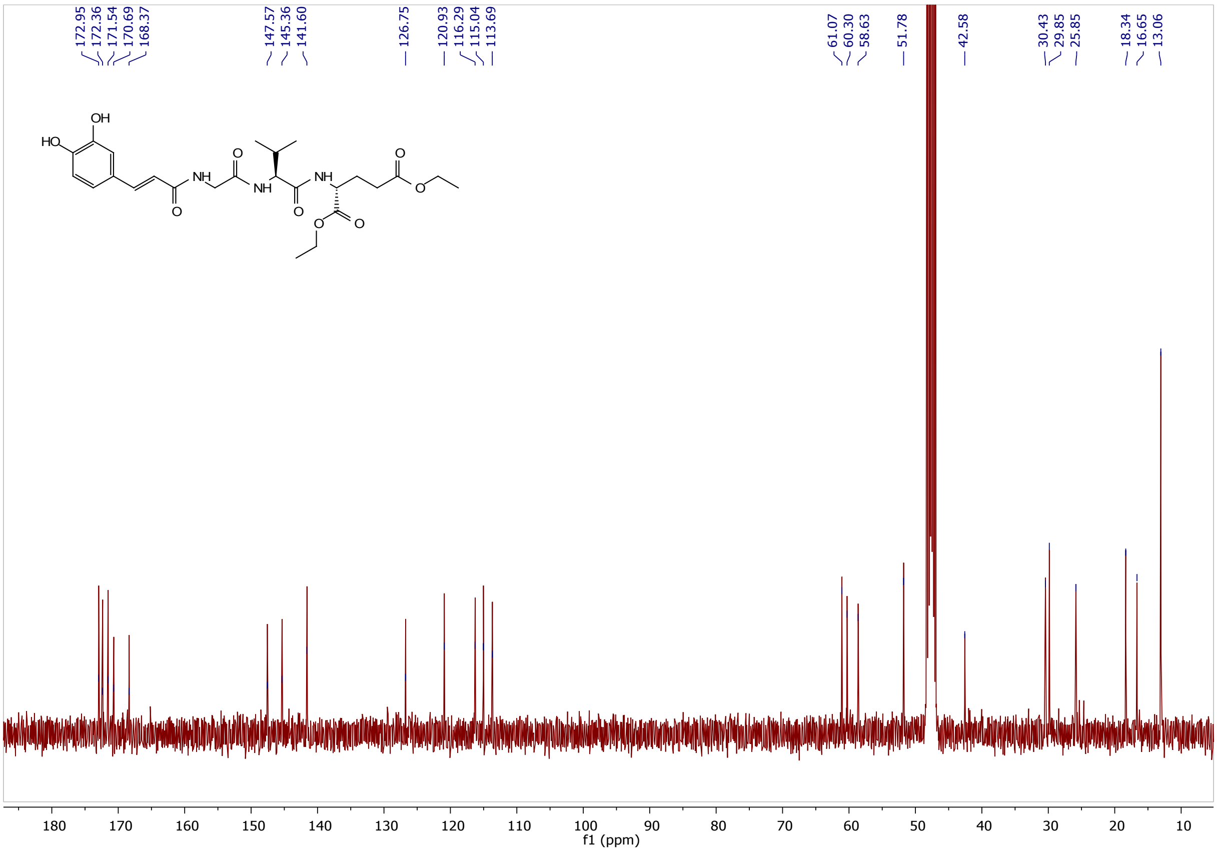

Compound **24**: ^{1}H, 400 MHz, MeOD

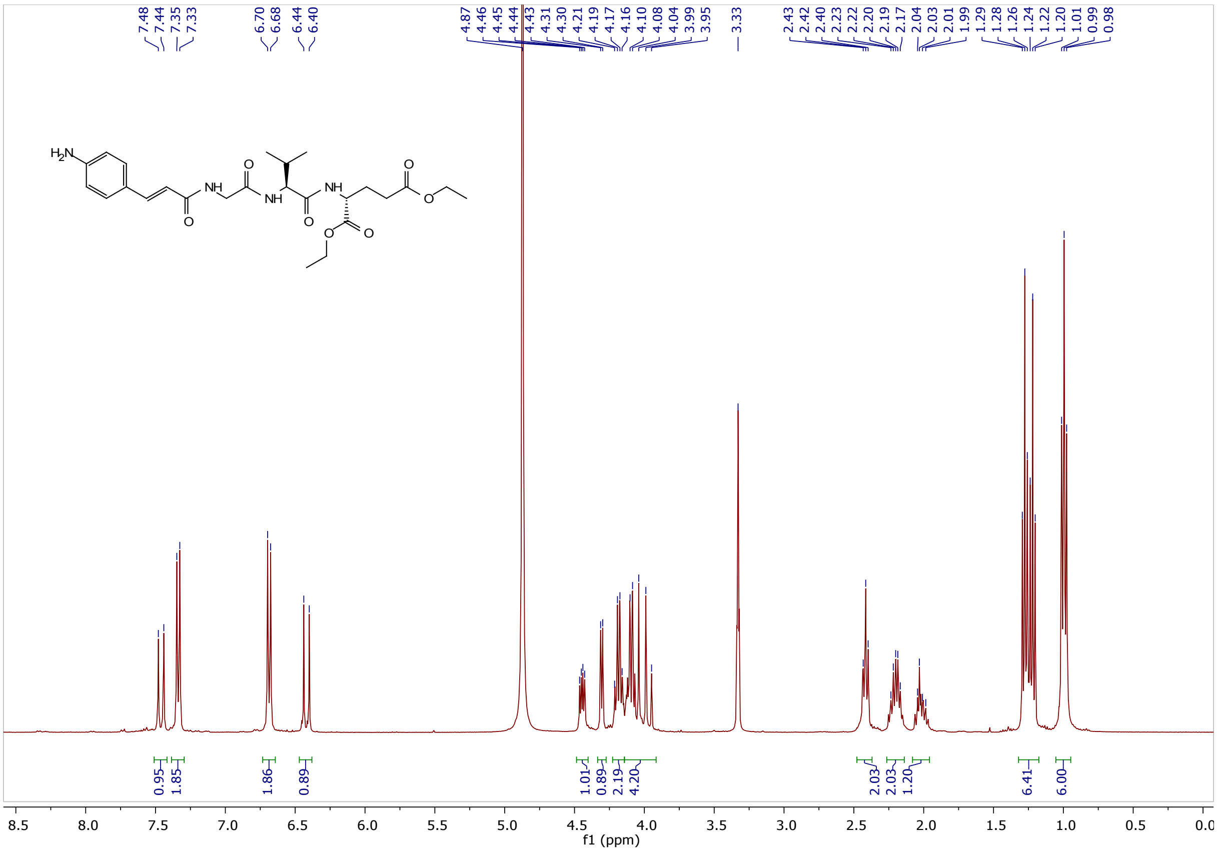

Compound **24**: ^{13}C, 100 MHz, MeOD

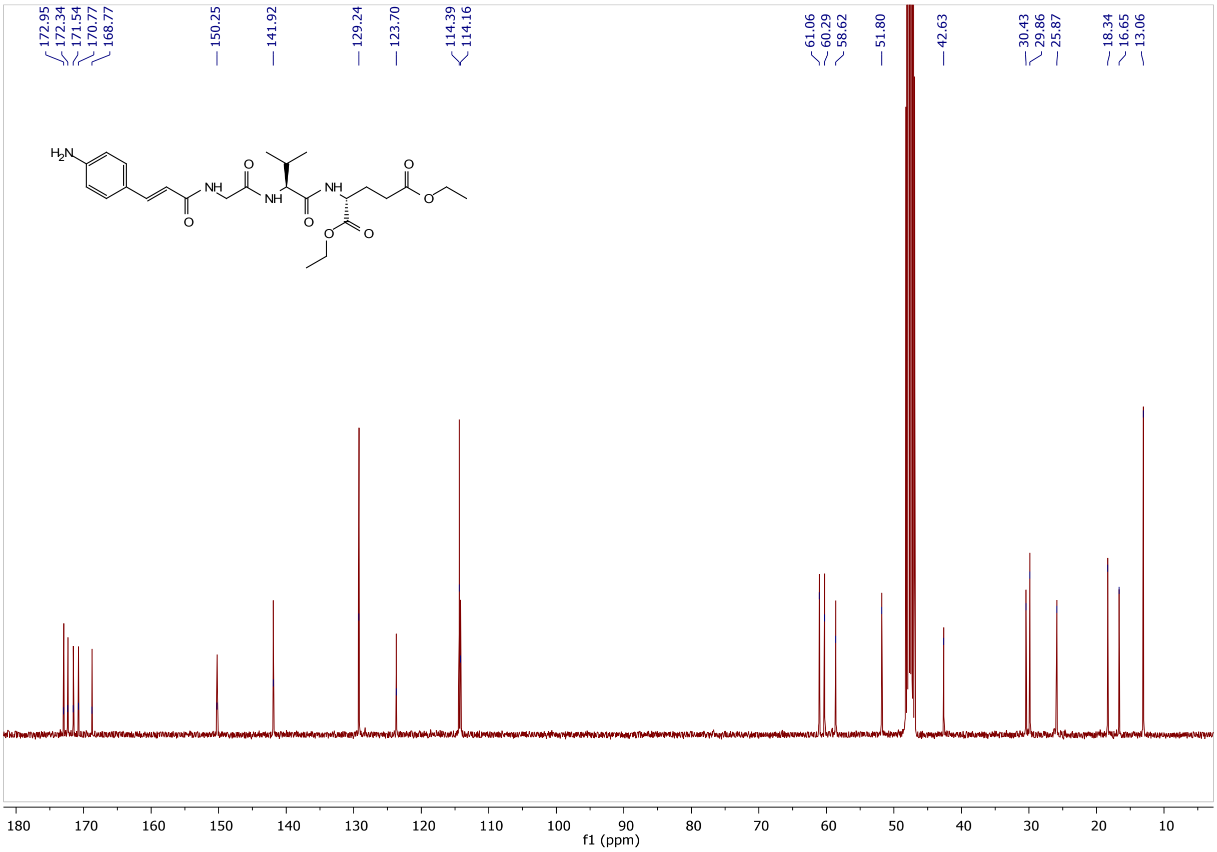

Compound **25**: ^{1}H, 400 MHz, DMSO-d$_6$

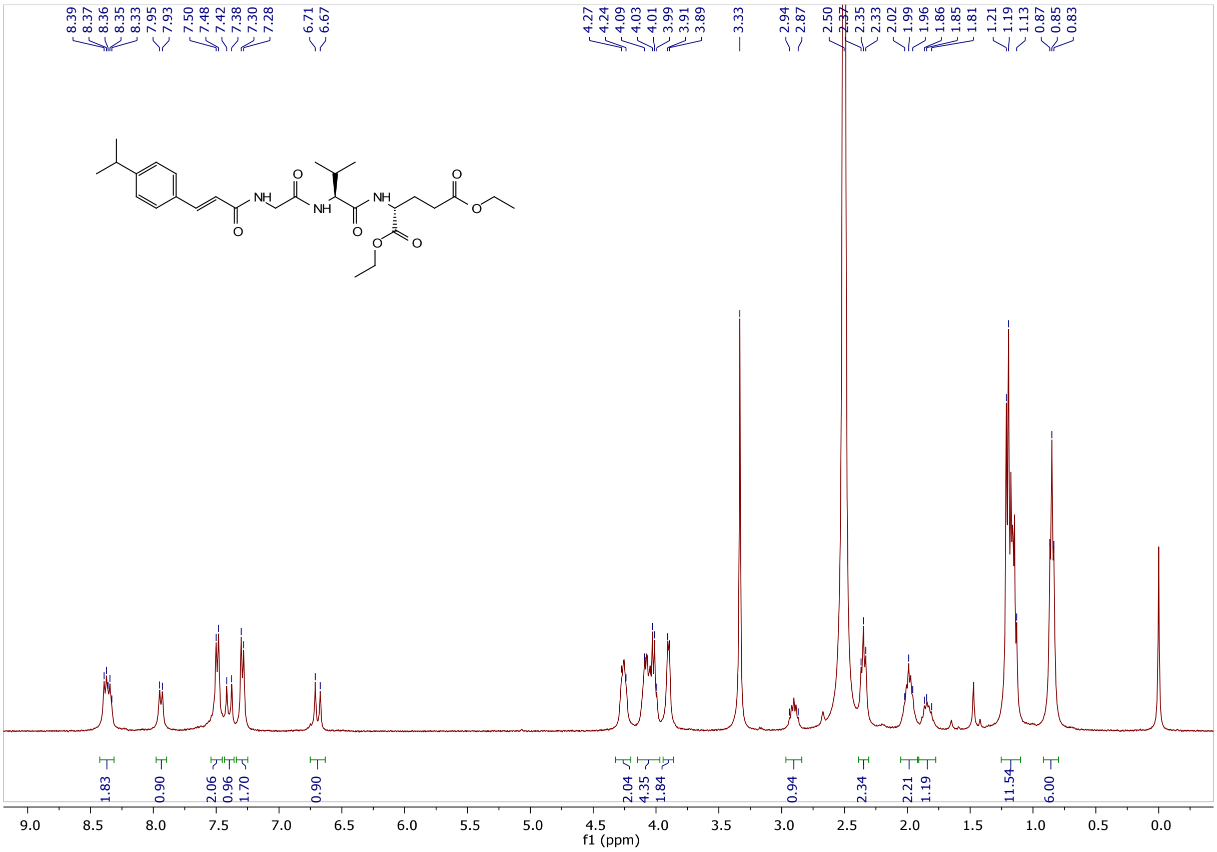

Compound **25**: ^{13}C, 100 MHz, DMSO-d$_6$

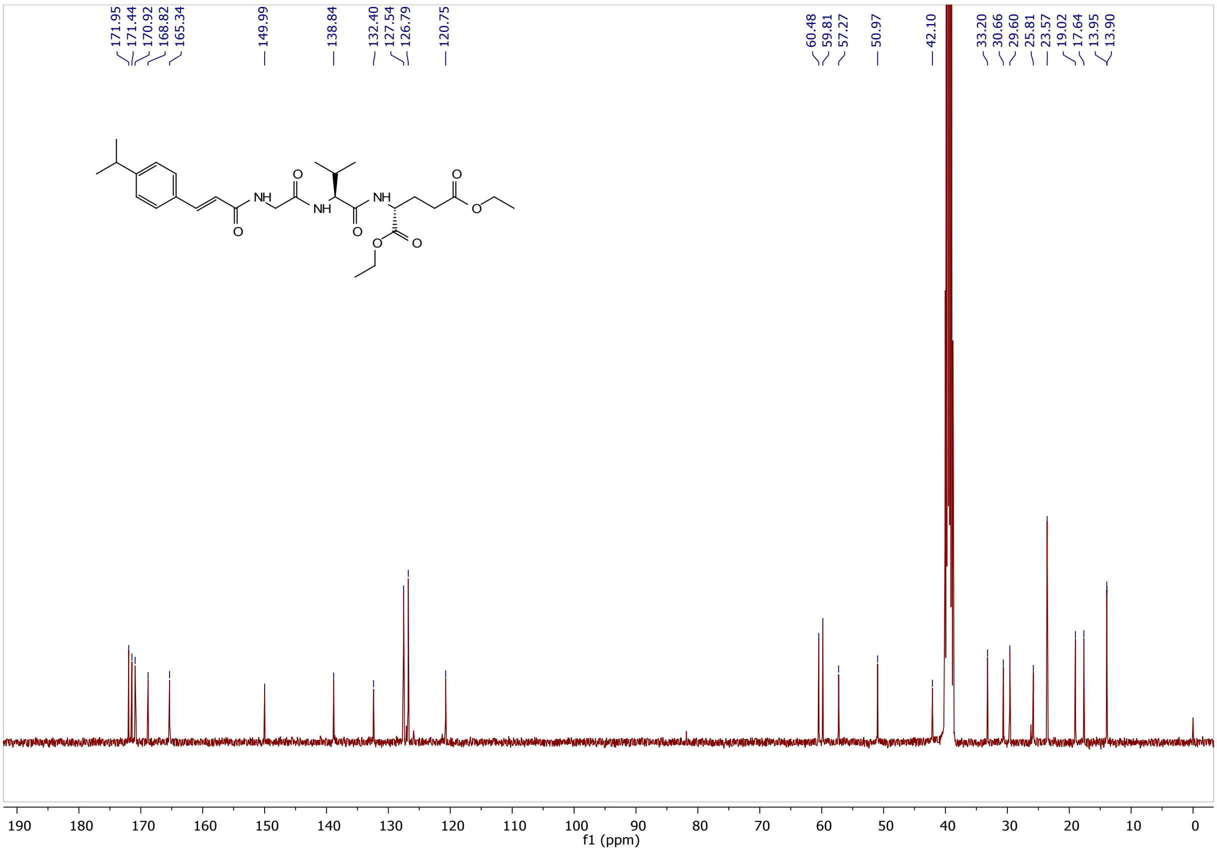

Compound **26**: ^{1}H, 400 MHz, DMSO-d$_6$

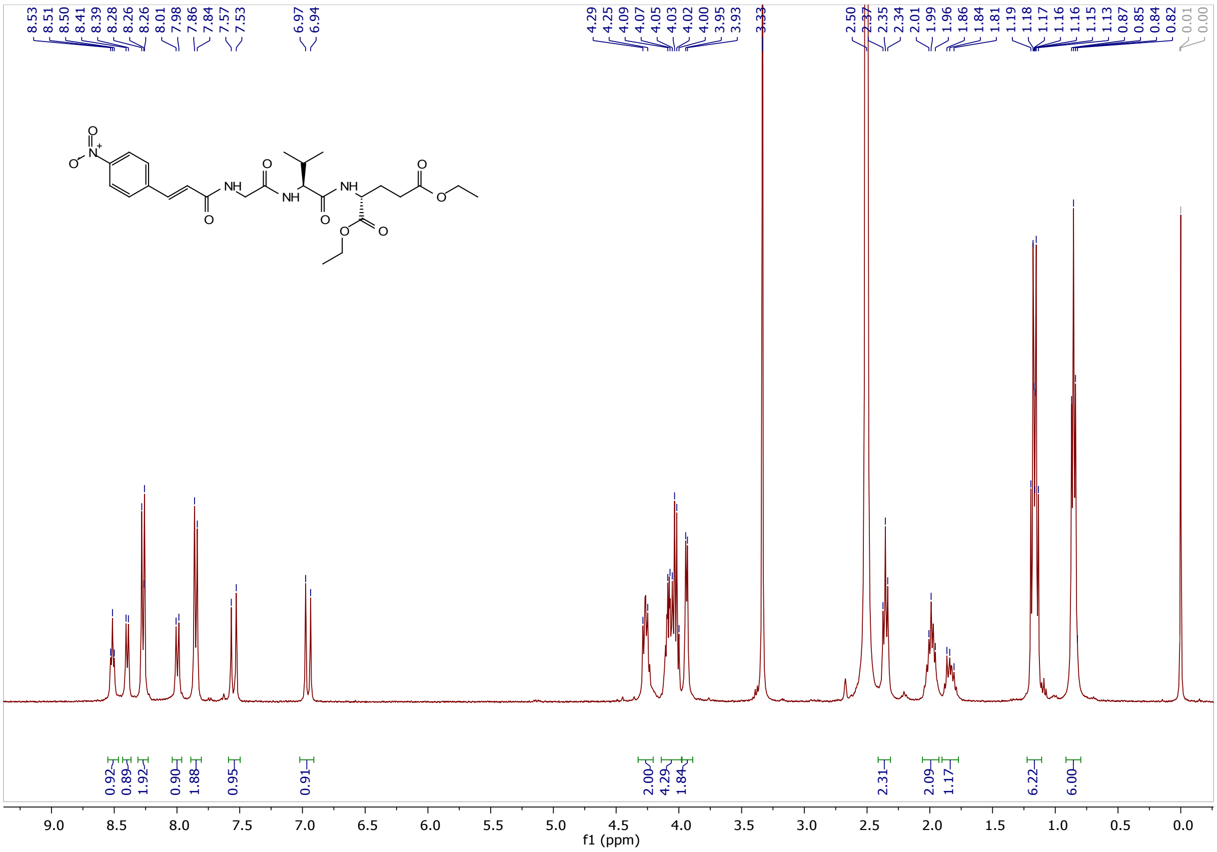

Compound **26**: ^{13}C, 100 MHz, DMSO-d$_6$

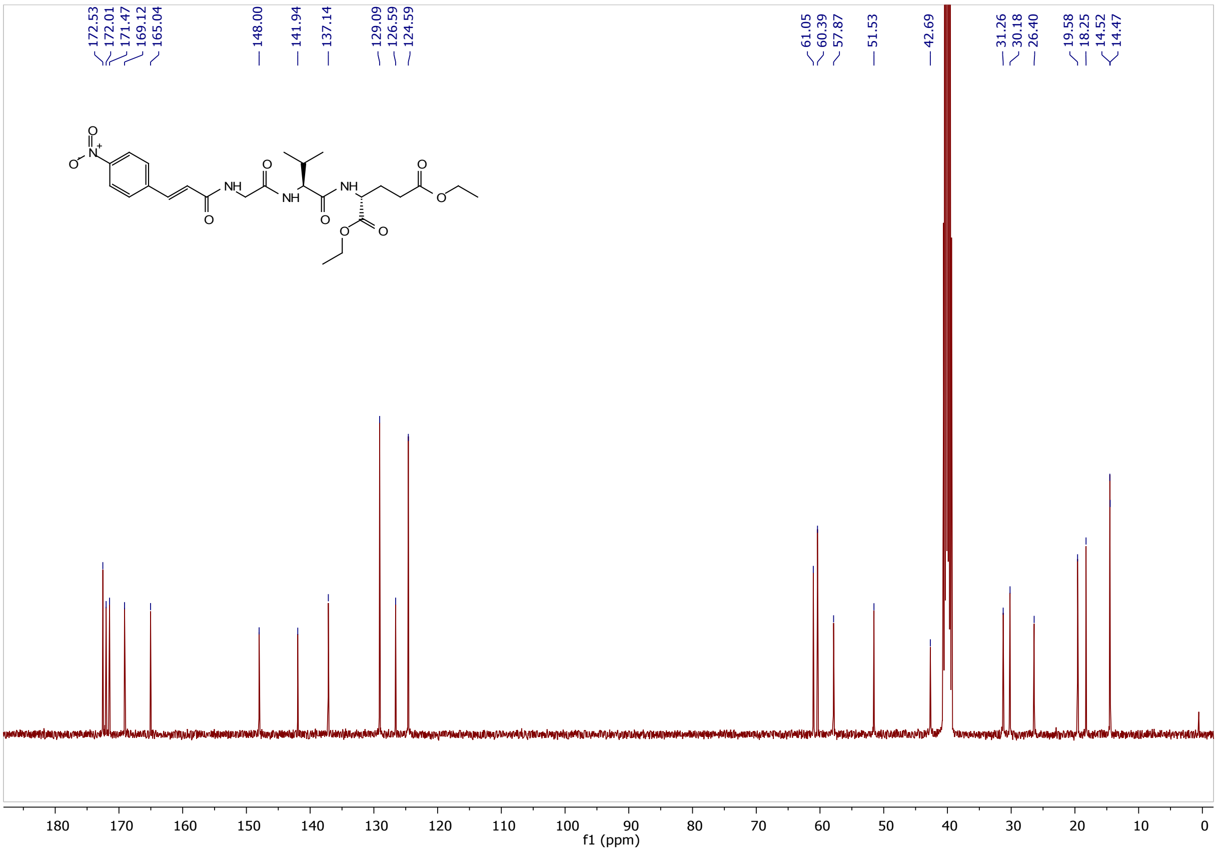

Compound **27**: ^{1}H, 400 MHz, DMSO-d_6

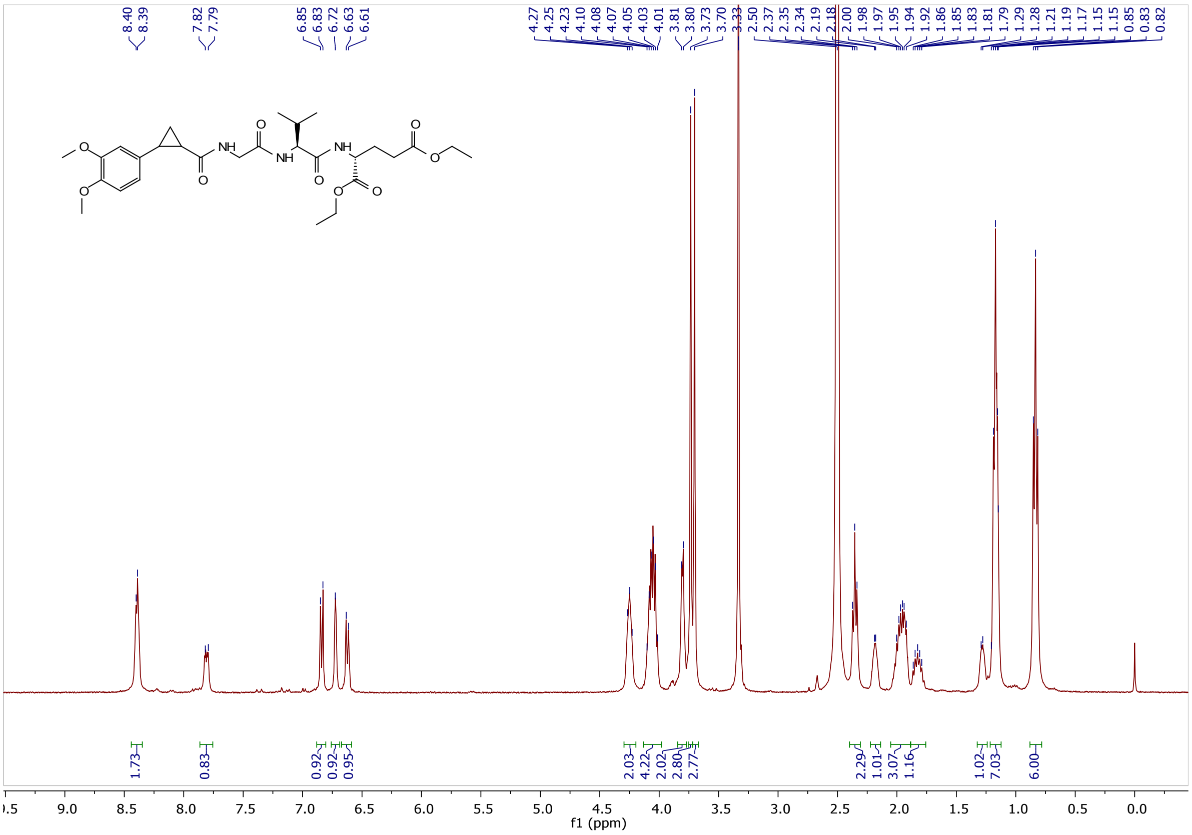

Compound **27**: ^{13}C, 100 MHz, DMSO-d_6

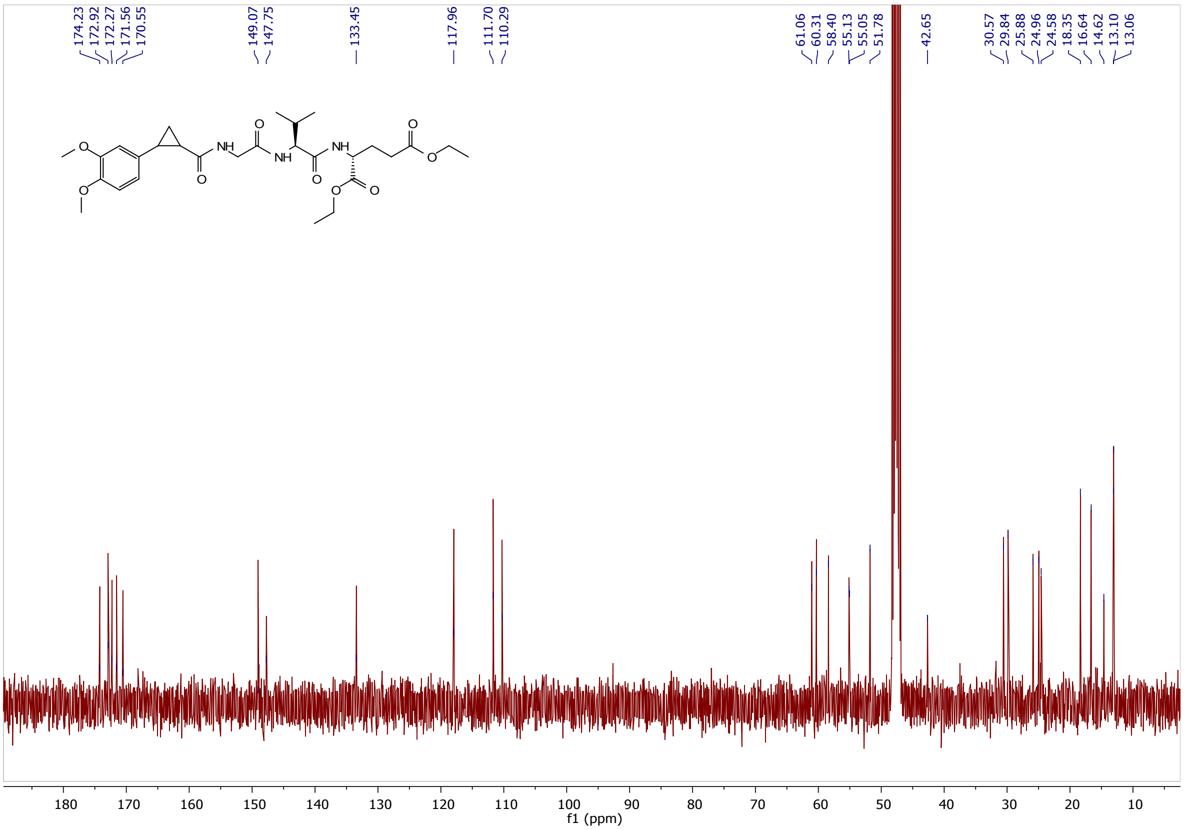

Compound **28**: ^{1}H, 400 MHz, DMSO-d$_6$

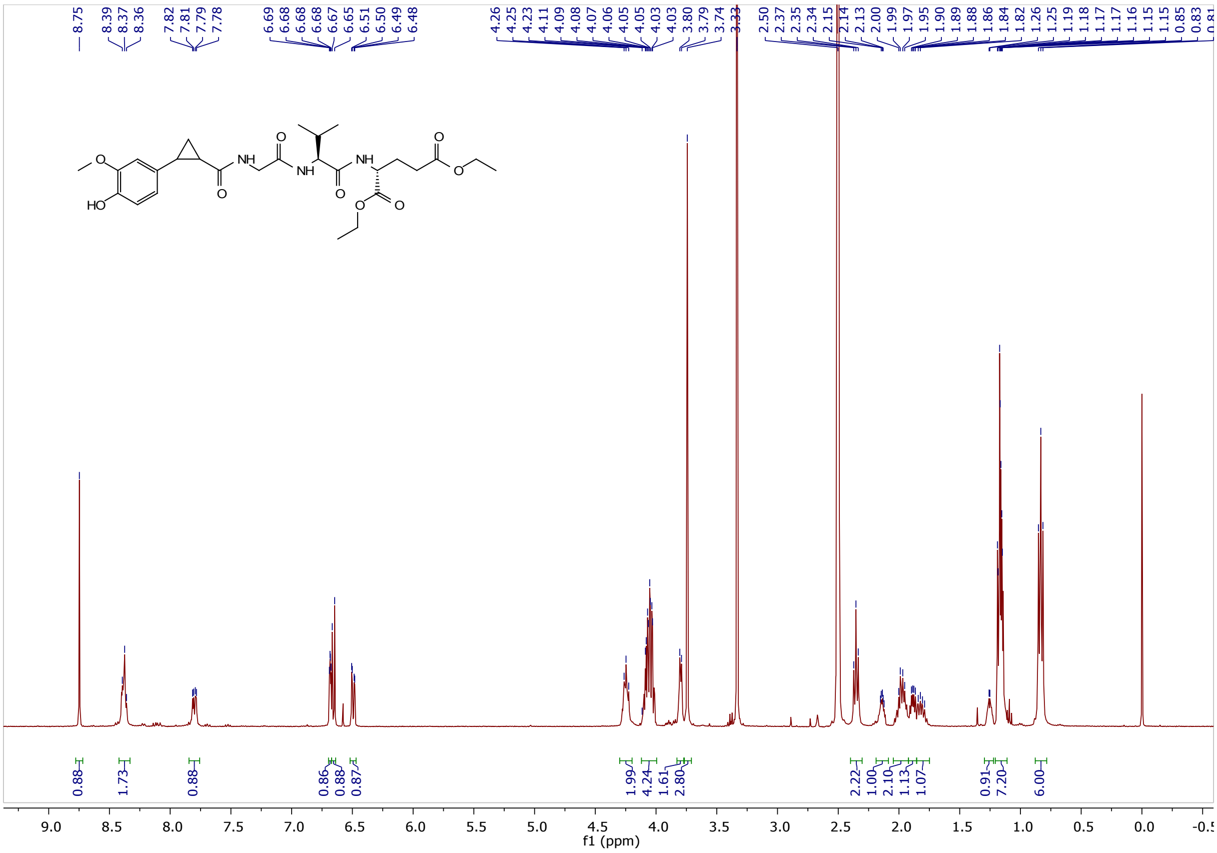

Compound **28**: ^{13}C, 100 MHz, DMSO-d$_6$

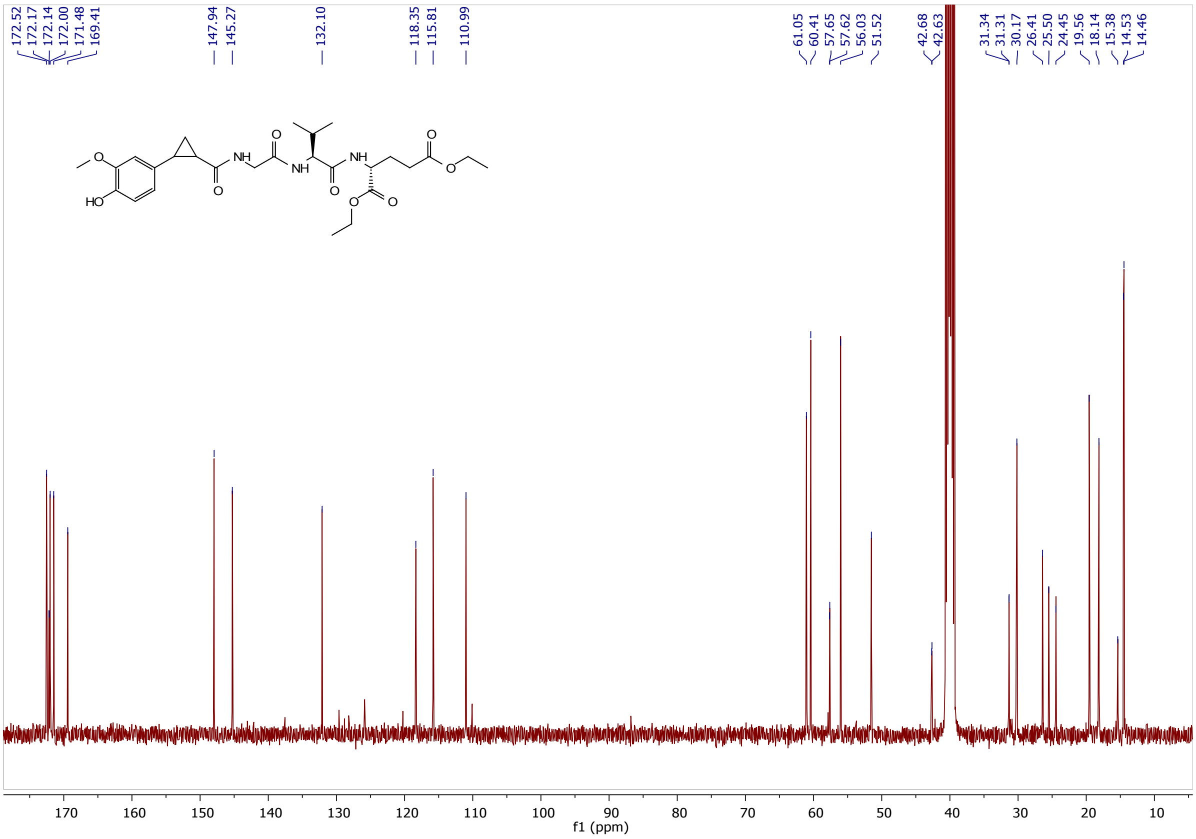

Compound **29**: ^{1}H, 400 MHz, DMSO-d$_6$

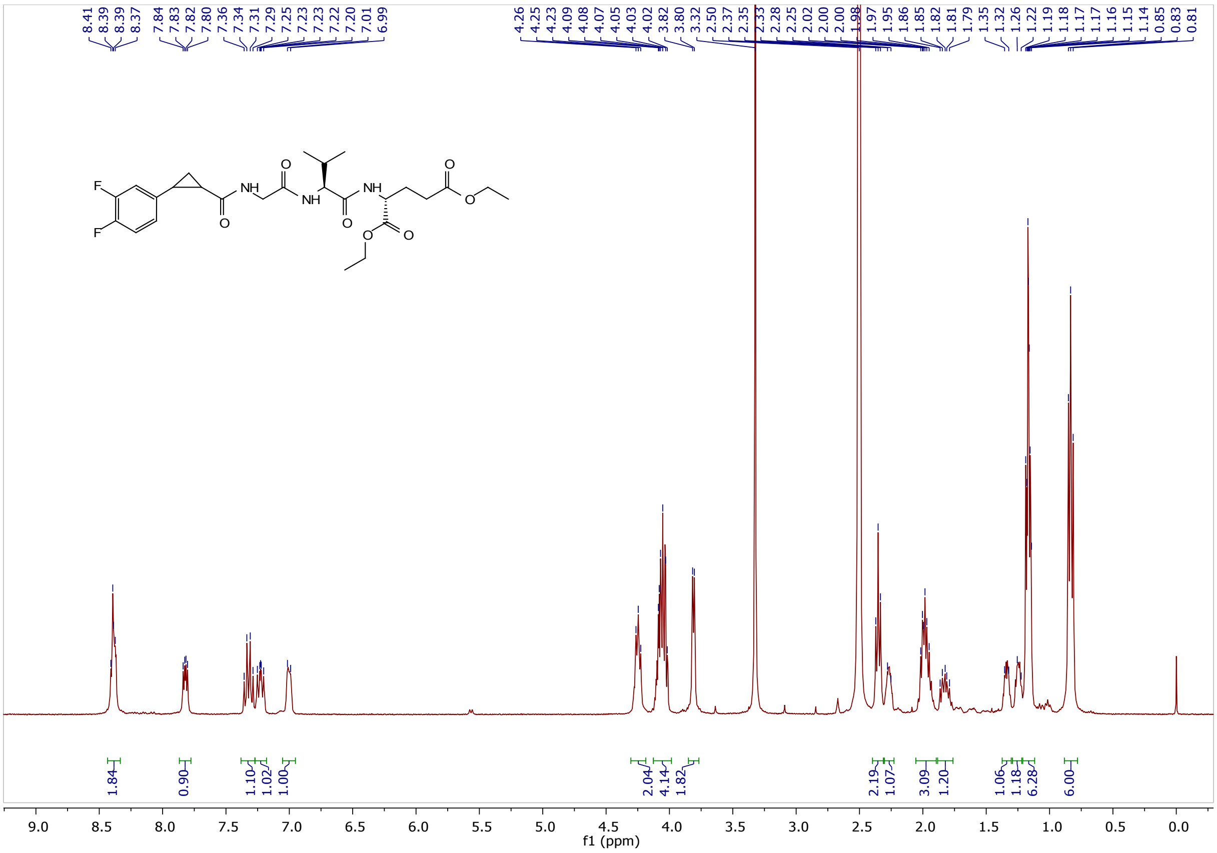

Compound **29**: ^{13}C, 100 MHz, DMSO-d$_6$

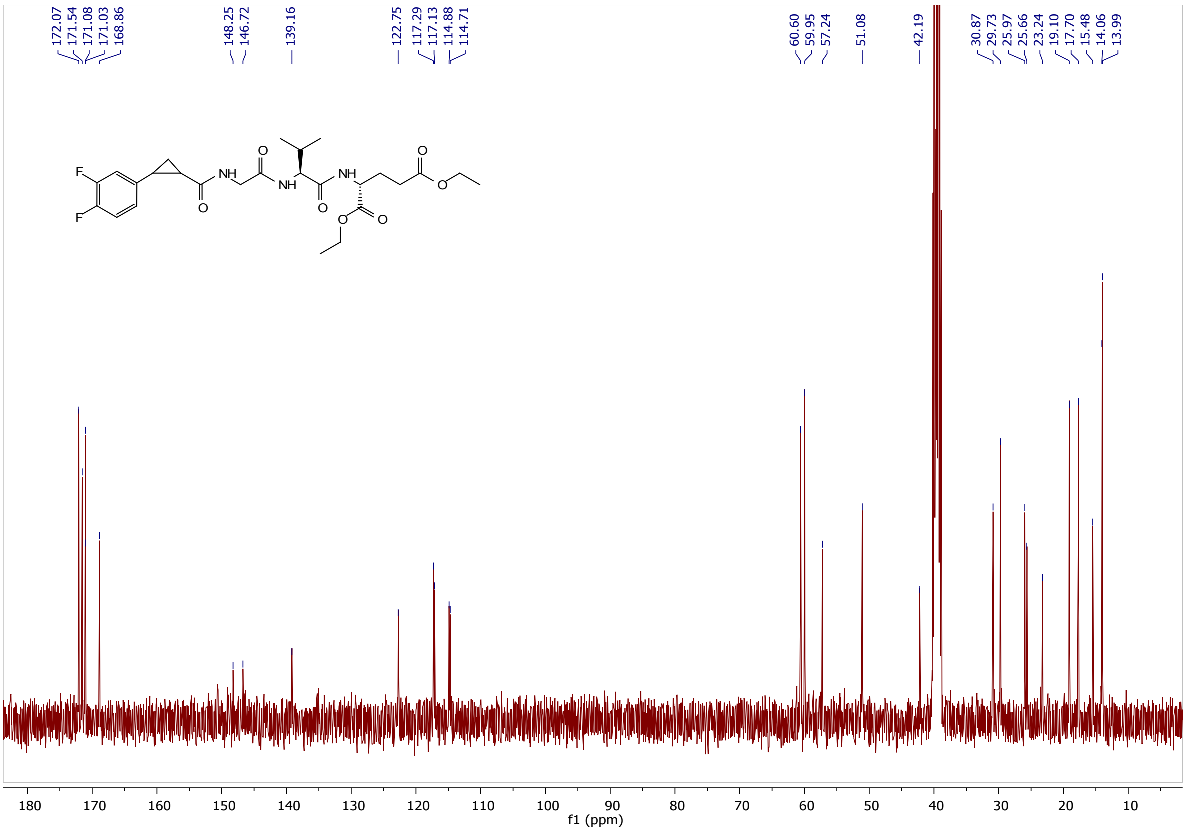

Compound **31**: ^{1}H, 400 MHz, MeOD

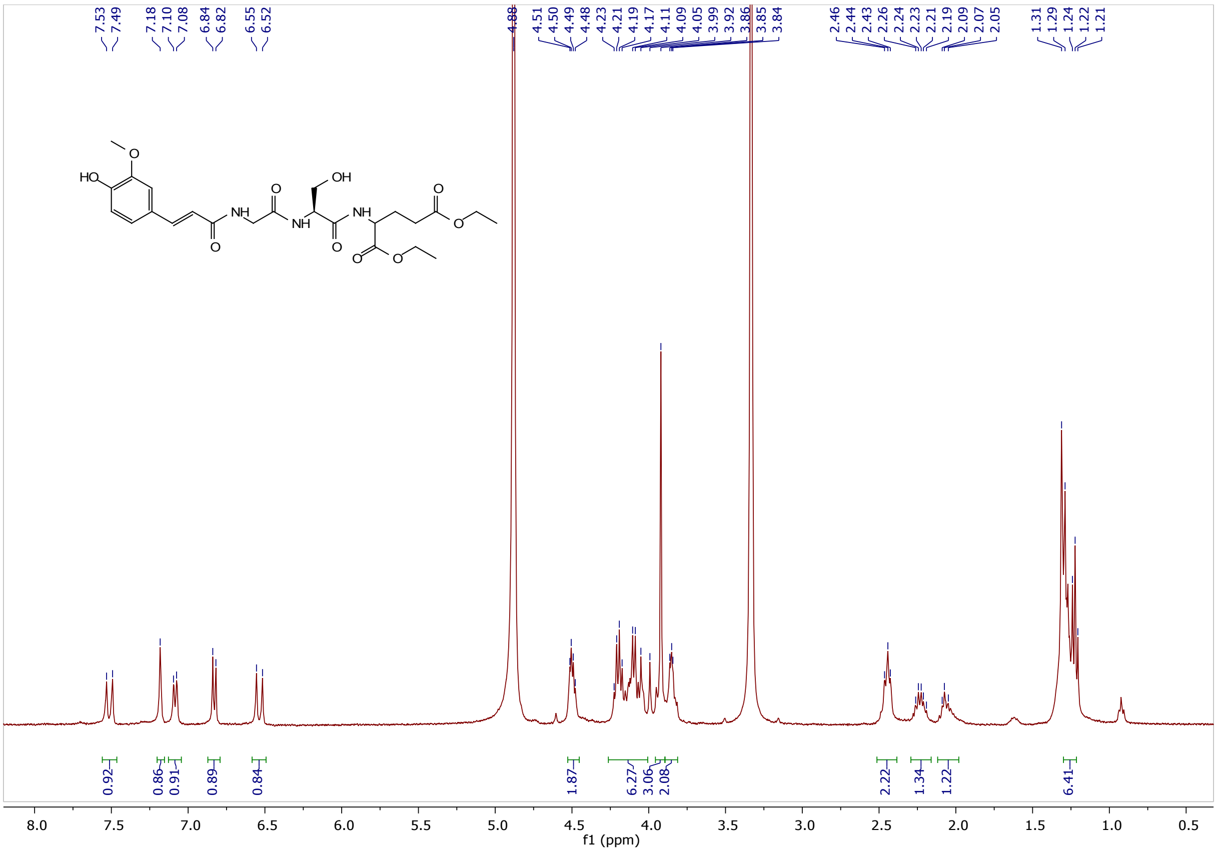

Compound **31**: ^{13}C, 100 MHz, MeOD

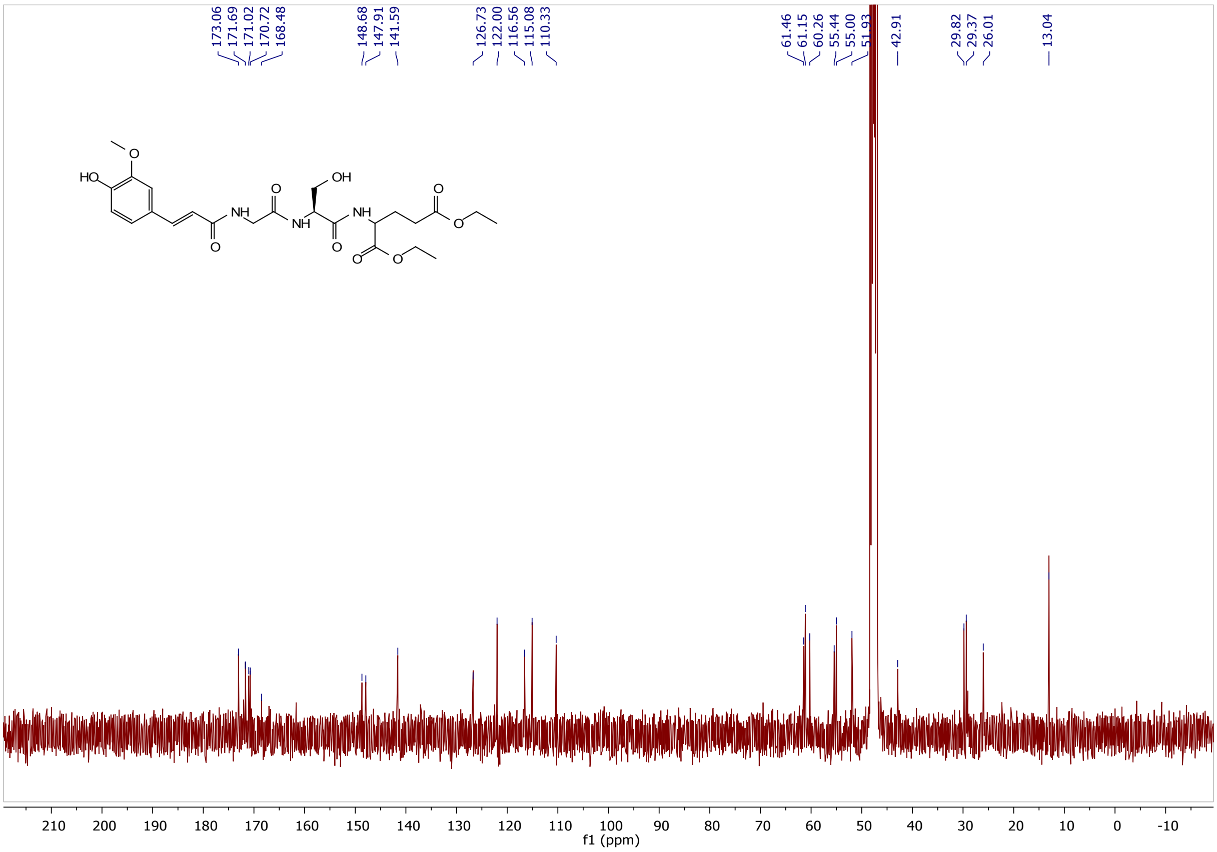

Compound **32**: ^{1}H, 400 MHz, $CDCl_3$

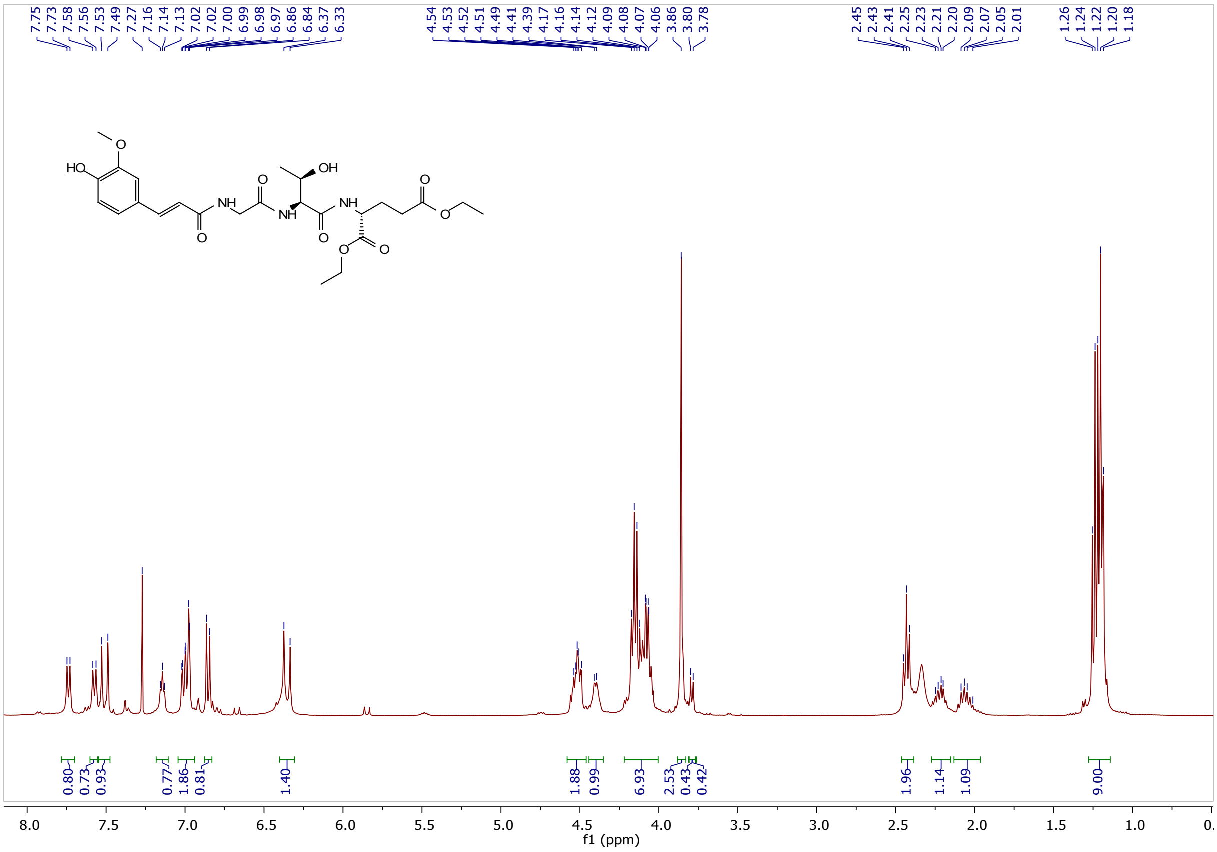

Compound **32**: ^{13}C, 100 MHz, $CDCl_3$

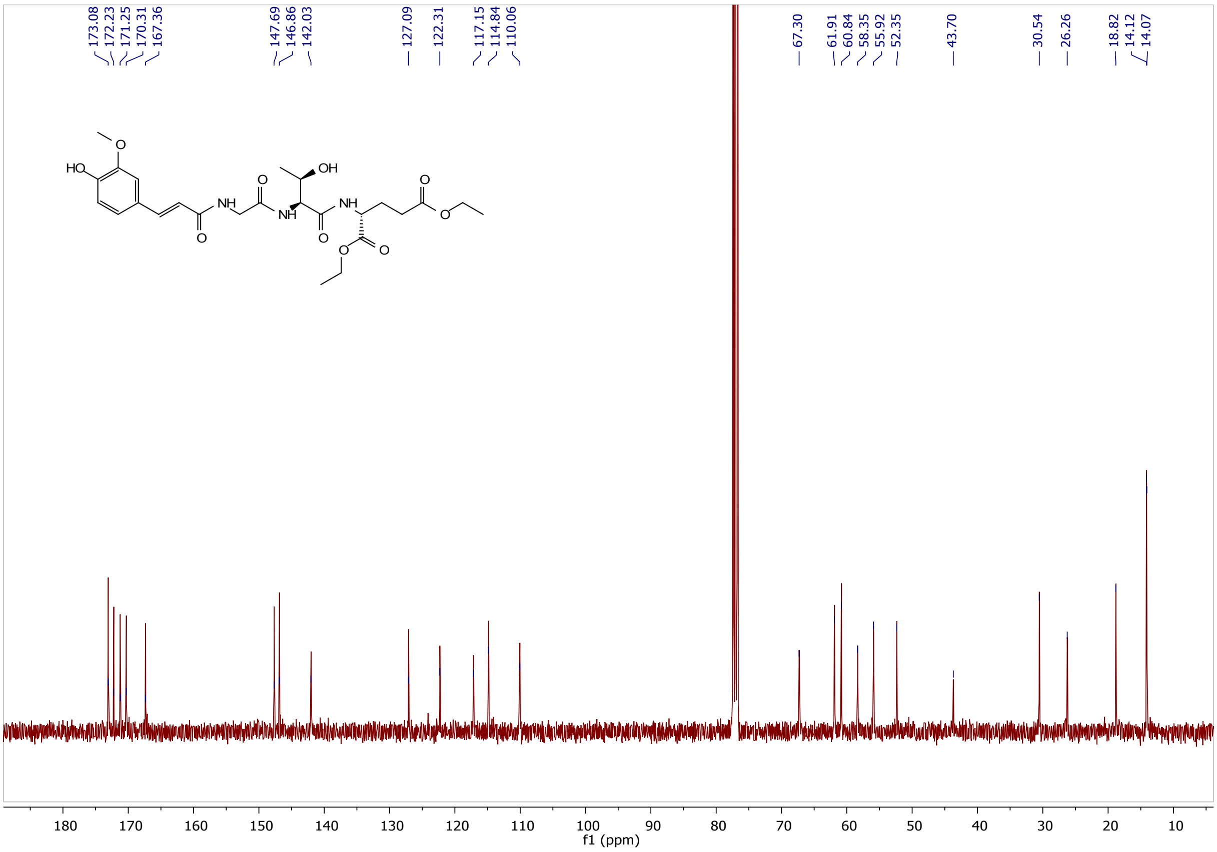

Compound **68**: ^{1}H, 400 MHz, $CDCl_3$

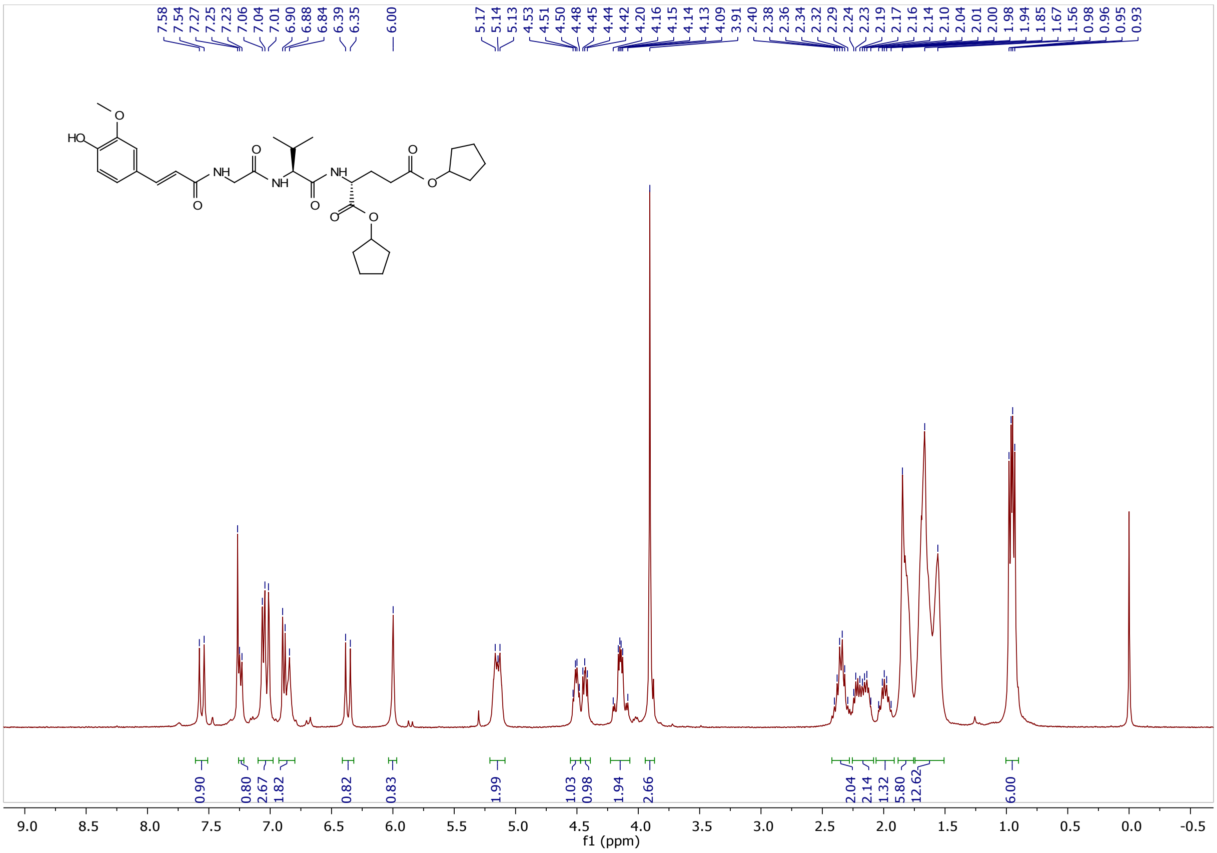

Compound **68**: ^{13}C, 100 MHz, $CDCl_3$

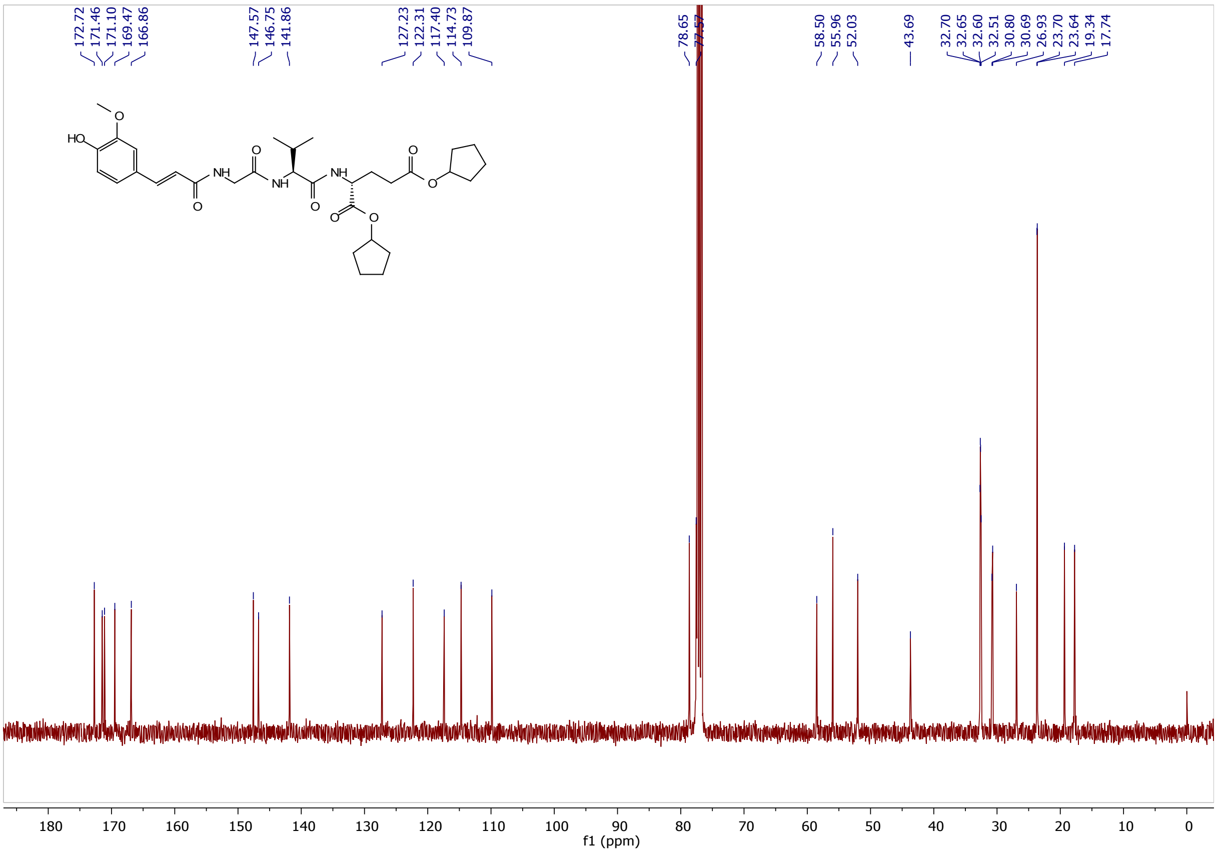

Compound **73**: ^{1}H, 400 MHz, DMSO-d_6

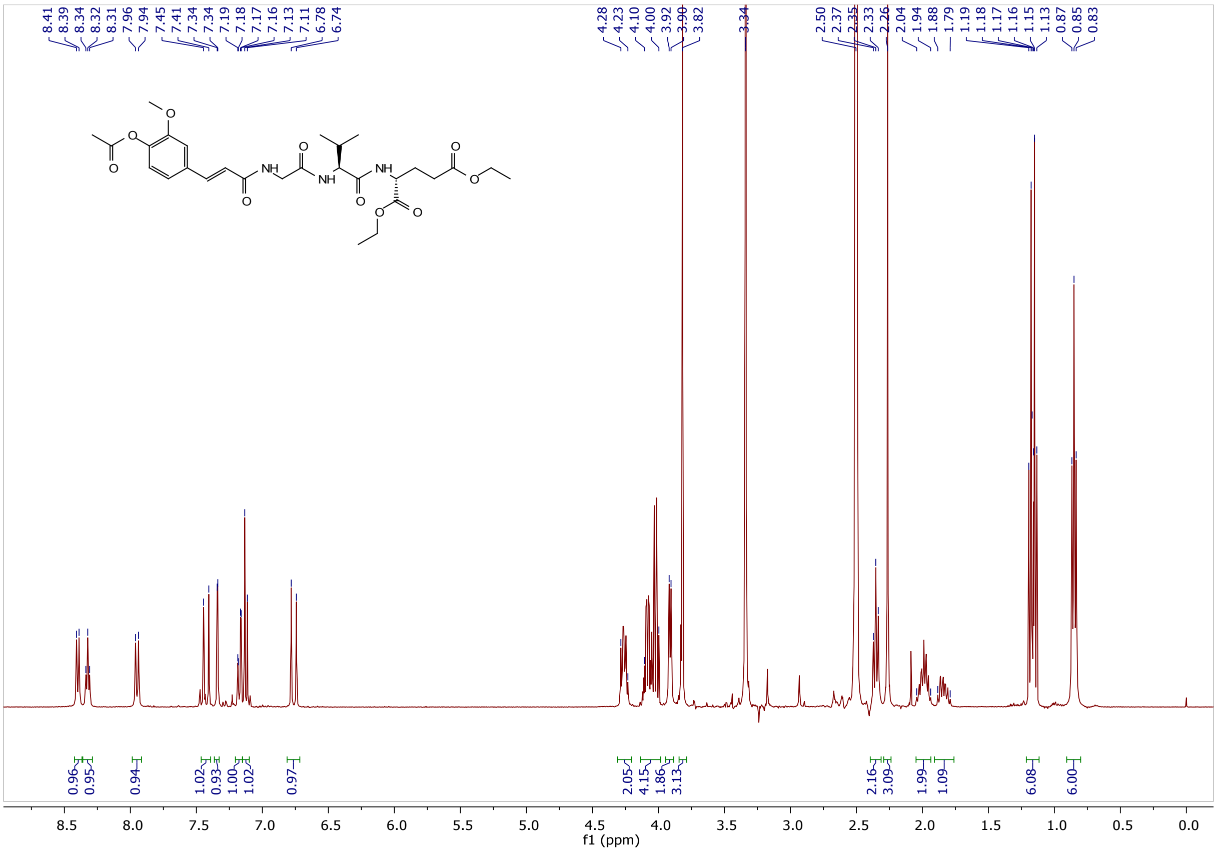

Compound **73**: ^{13}C, 100 MHz, DMSO-d_6

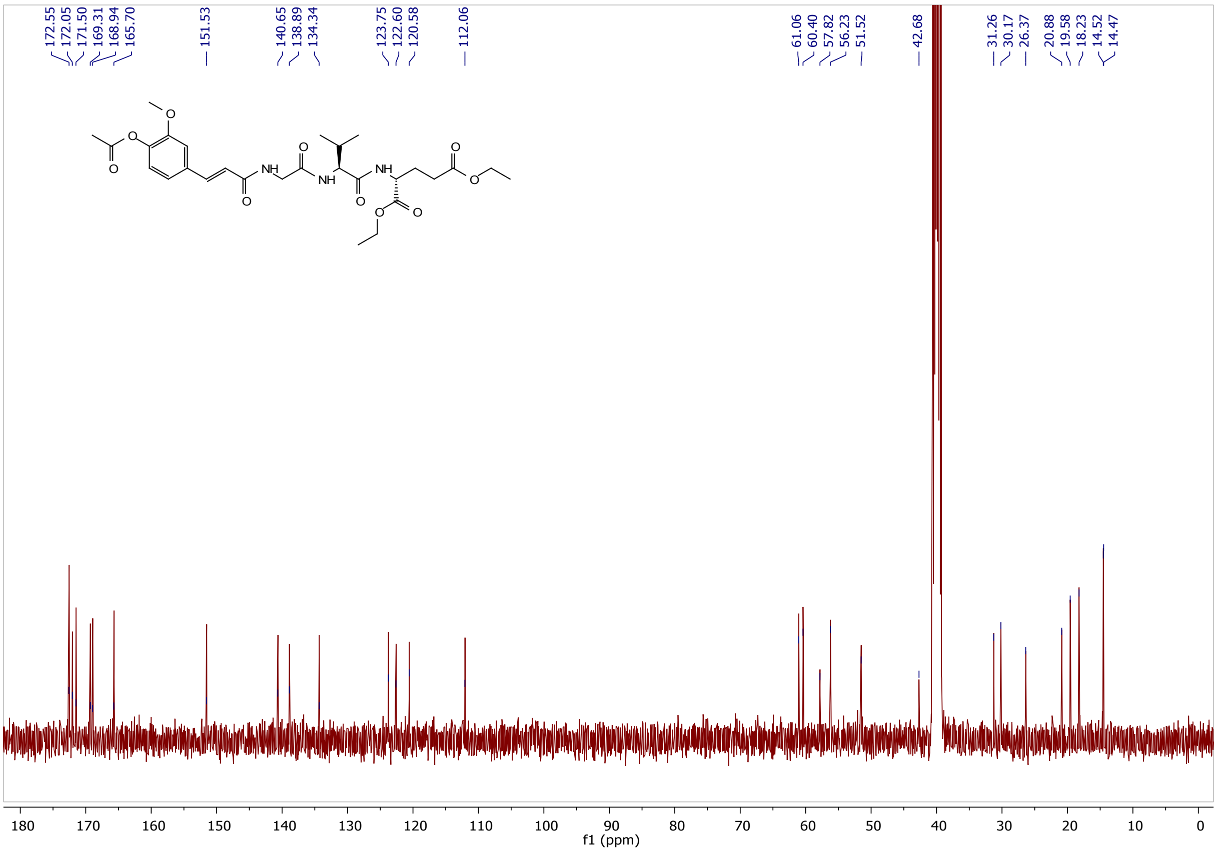

Compound **74**: ^{1}H, 400 MHz, $CDCl_3$

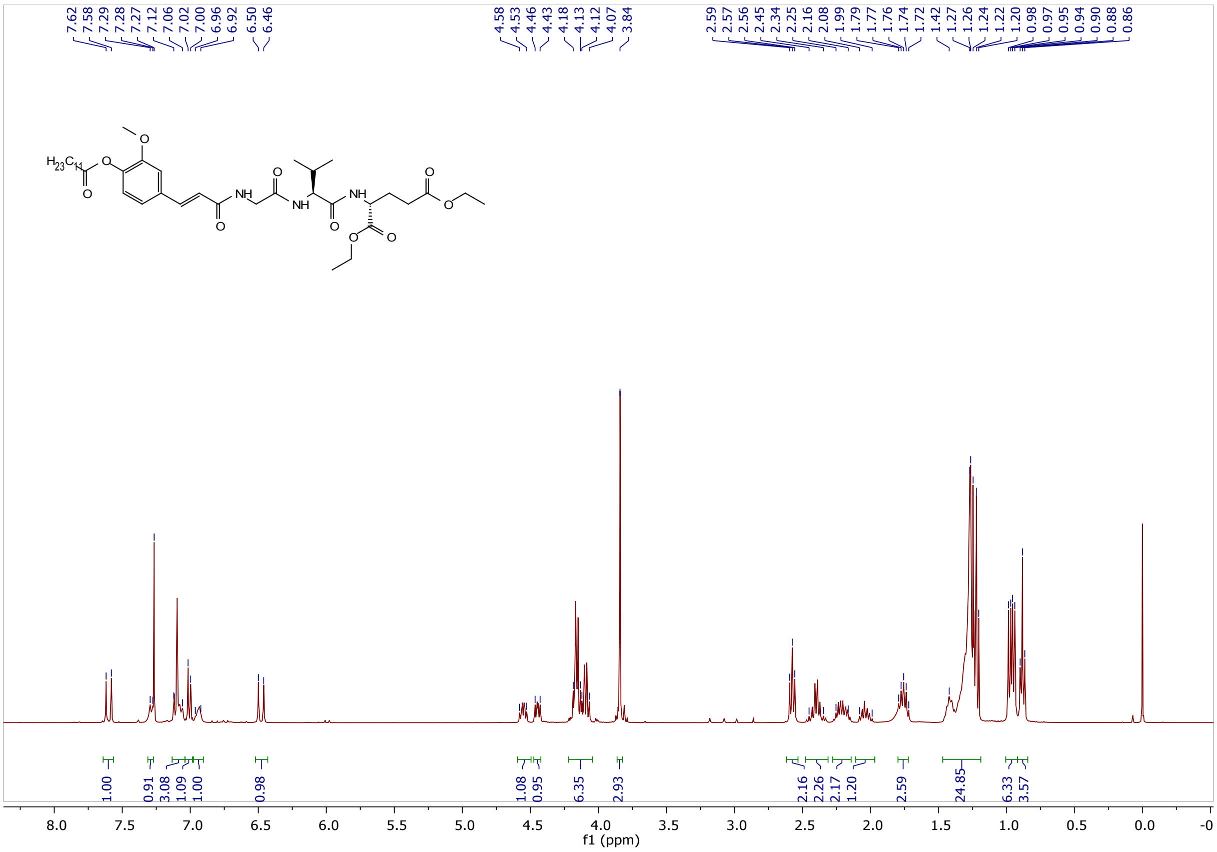

Compound **74**: ^{13}C, 100 MHz, $CDCl_3$

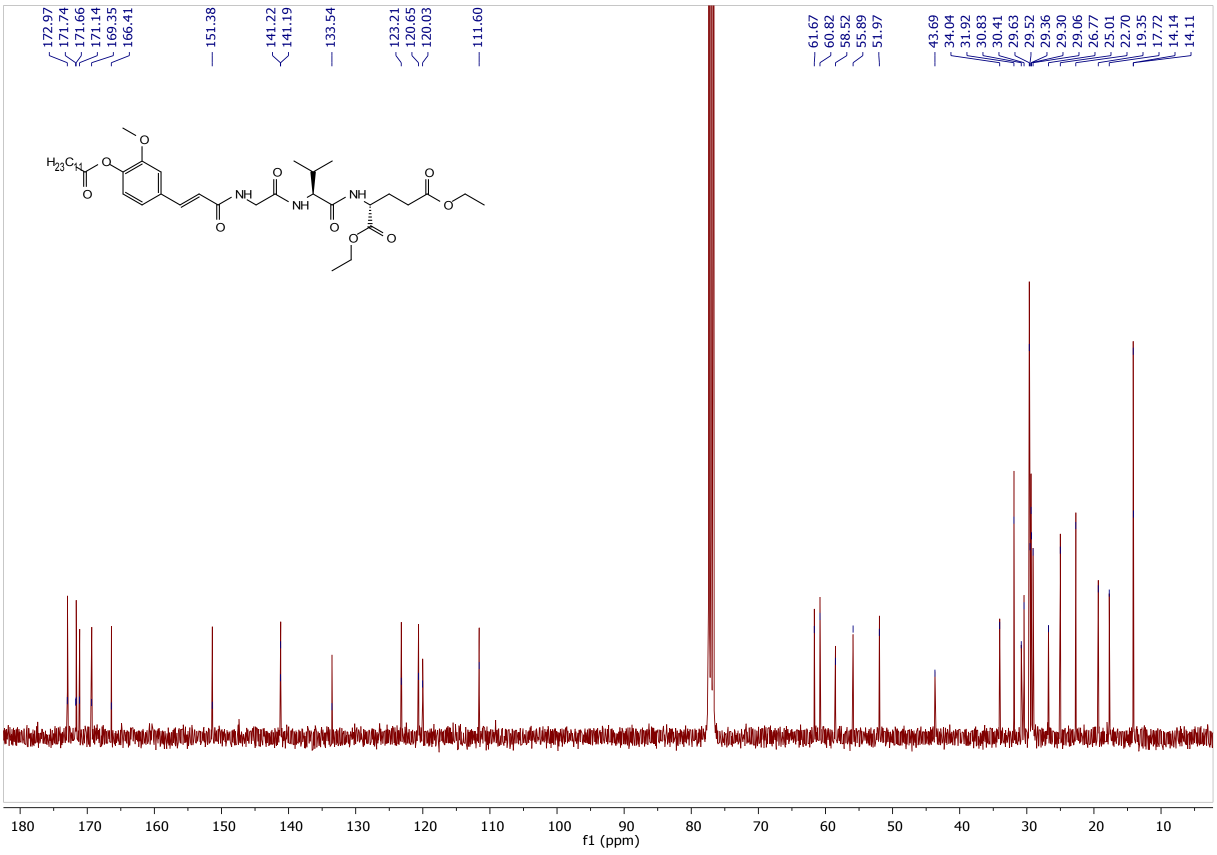

Compound **75**: ^{1}H, 400 MHz, $CDCl_3$

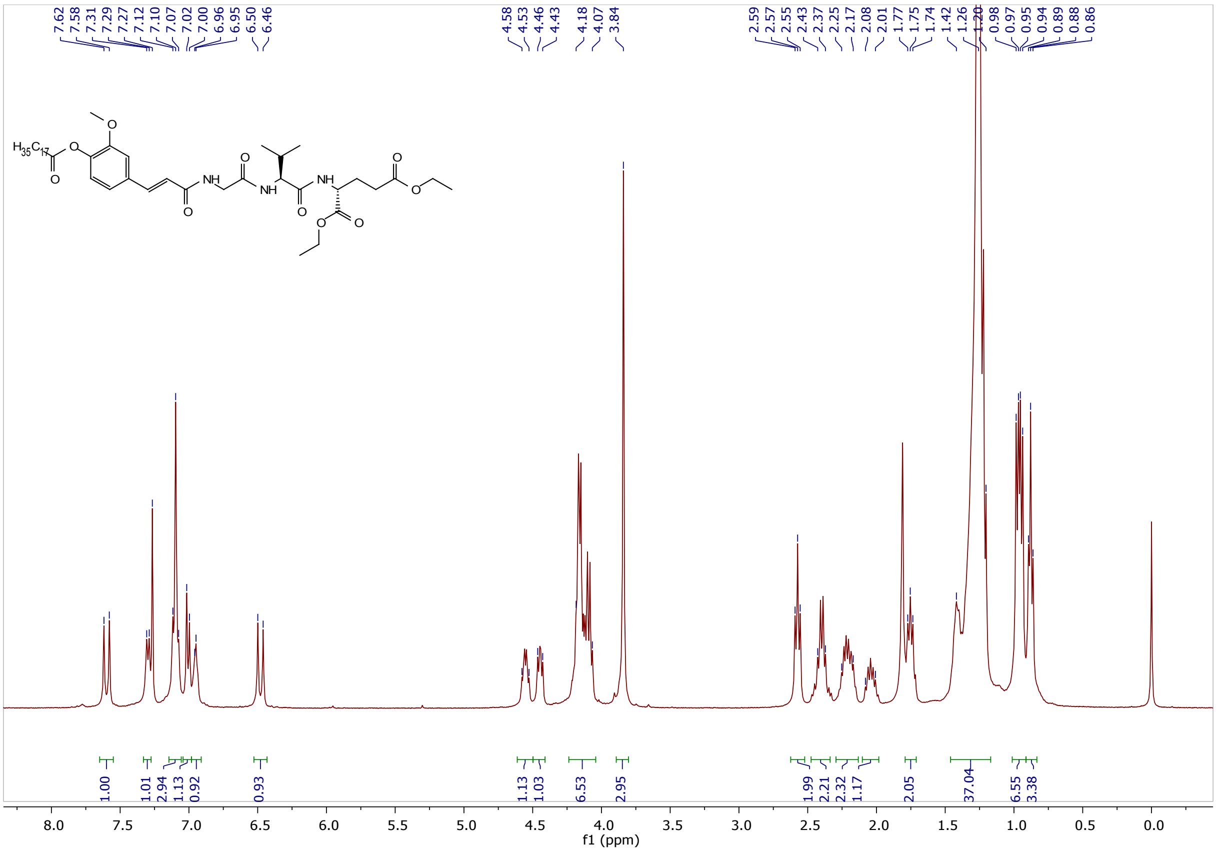

Compound **75**: ^{13}C, 100 MHz, $CDCl_3$

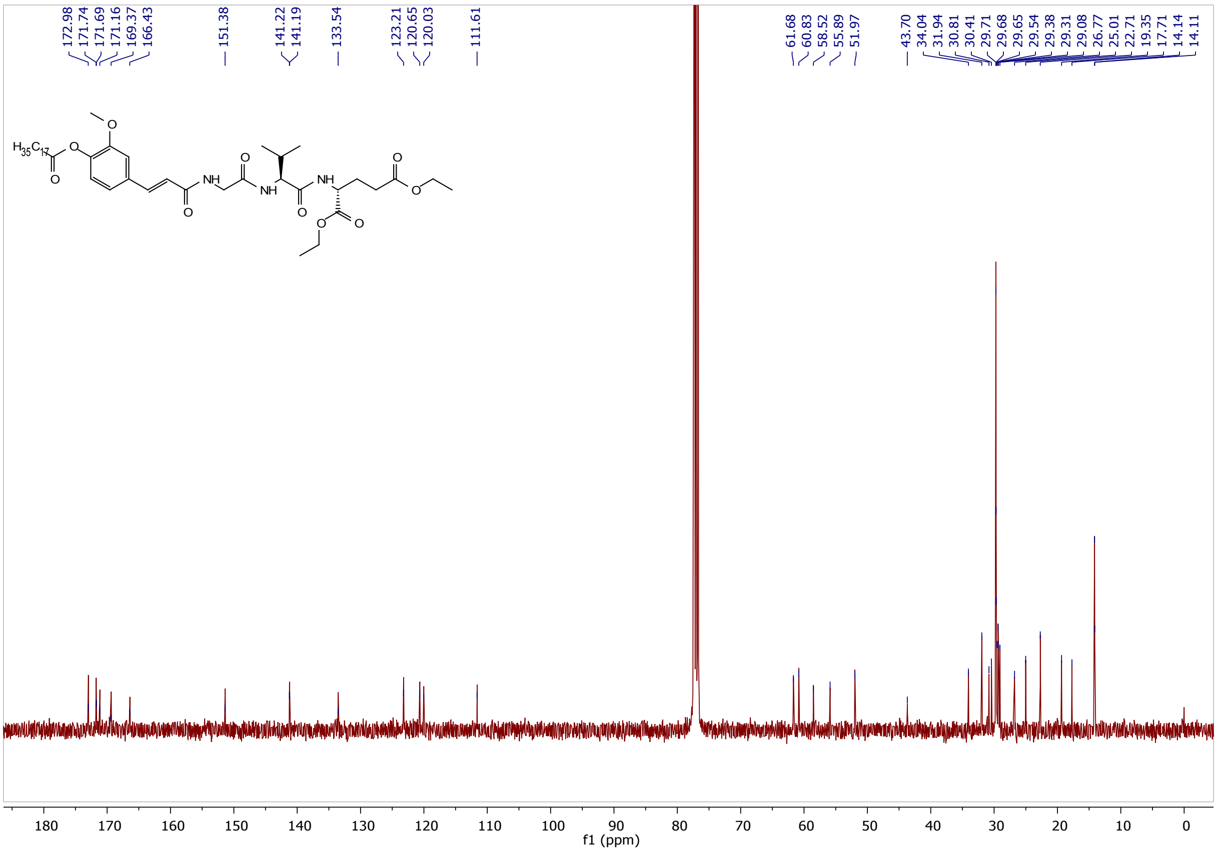

Compound **81**: ^{1}H, 400 MHz, $CDCl_3$

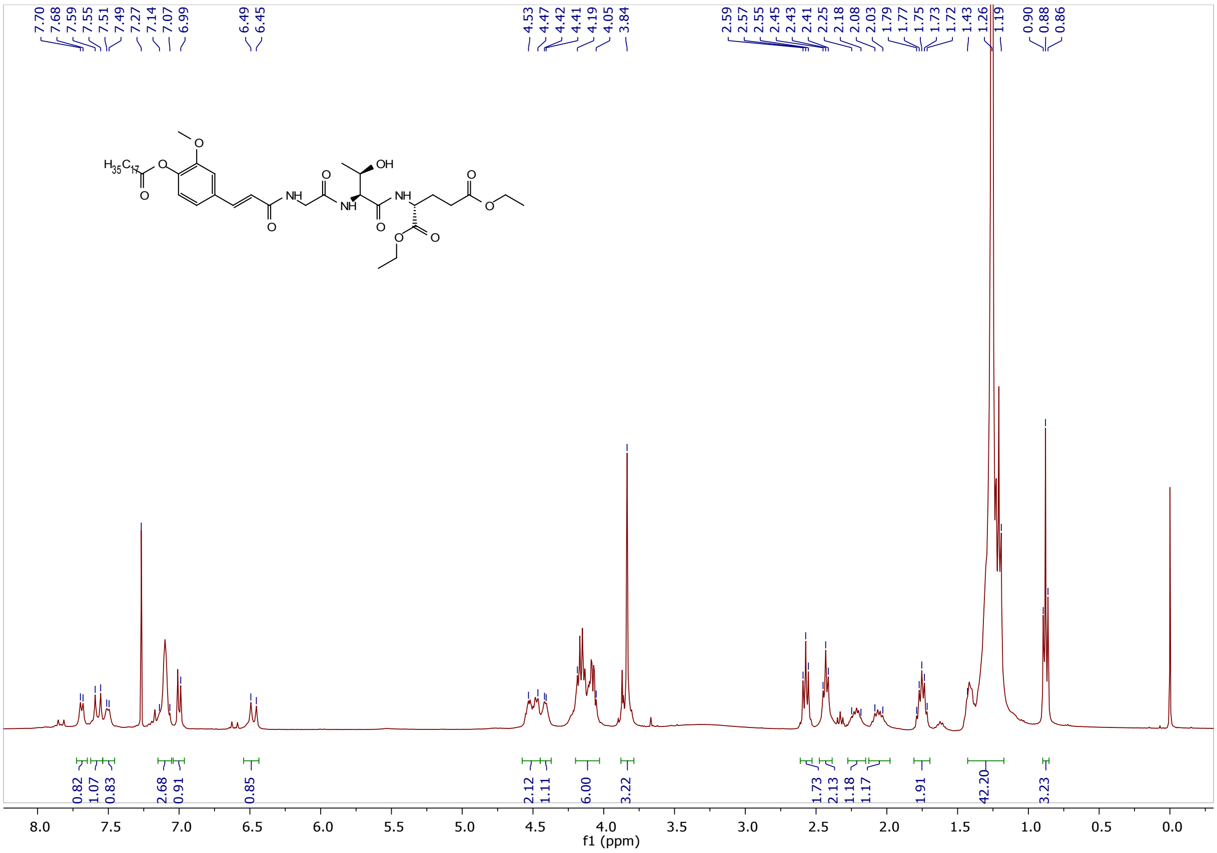

Compound **81**: ^{13}C, 100 MHz, $CDCl_3$

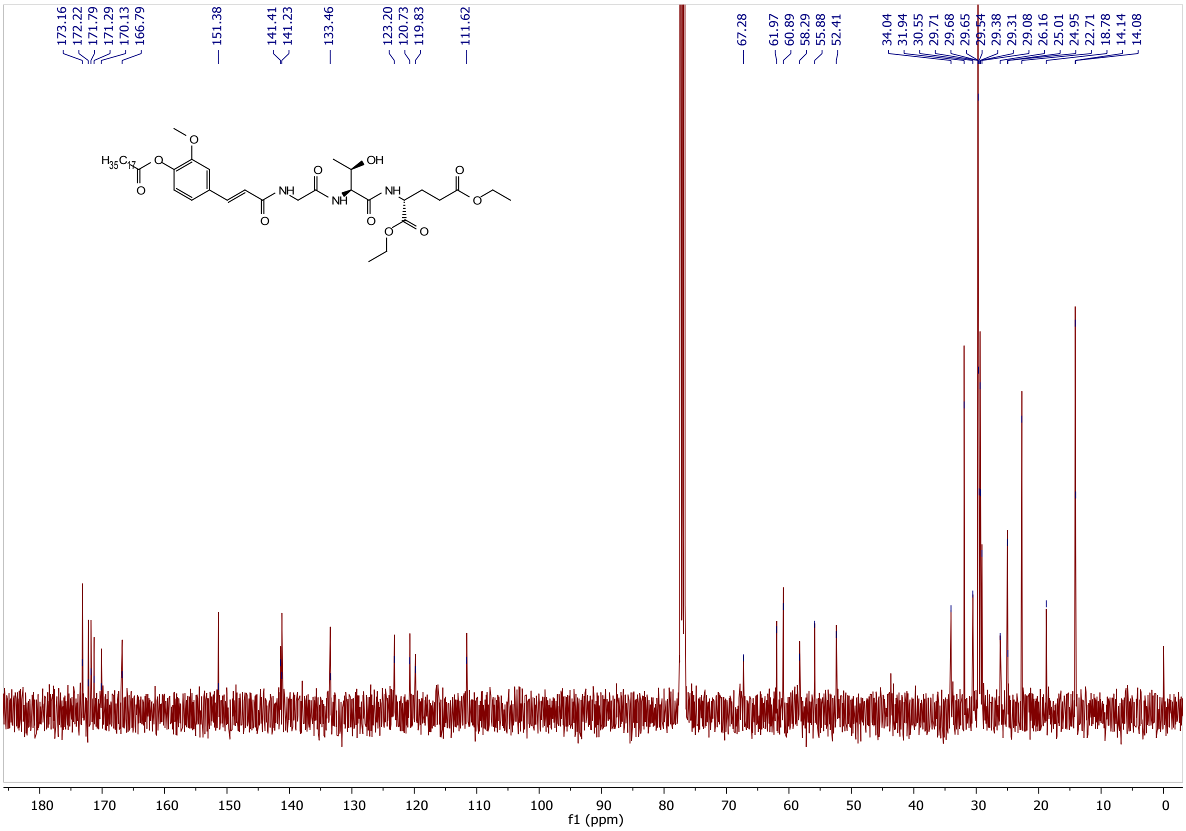

8 Select UHPLC traces

Compound **68**

Chromatogram and Results

Injection Details

Injection Name:	**Compound 68**	*Run Time (min):*	**12,50**
Processing Method:	**Quantitative**	*Injection Volume:*	**5,00**
Injection Date/Time:	**10.apr..19 13:10**	*Wavelength:*	**254**

Chromatogram

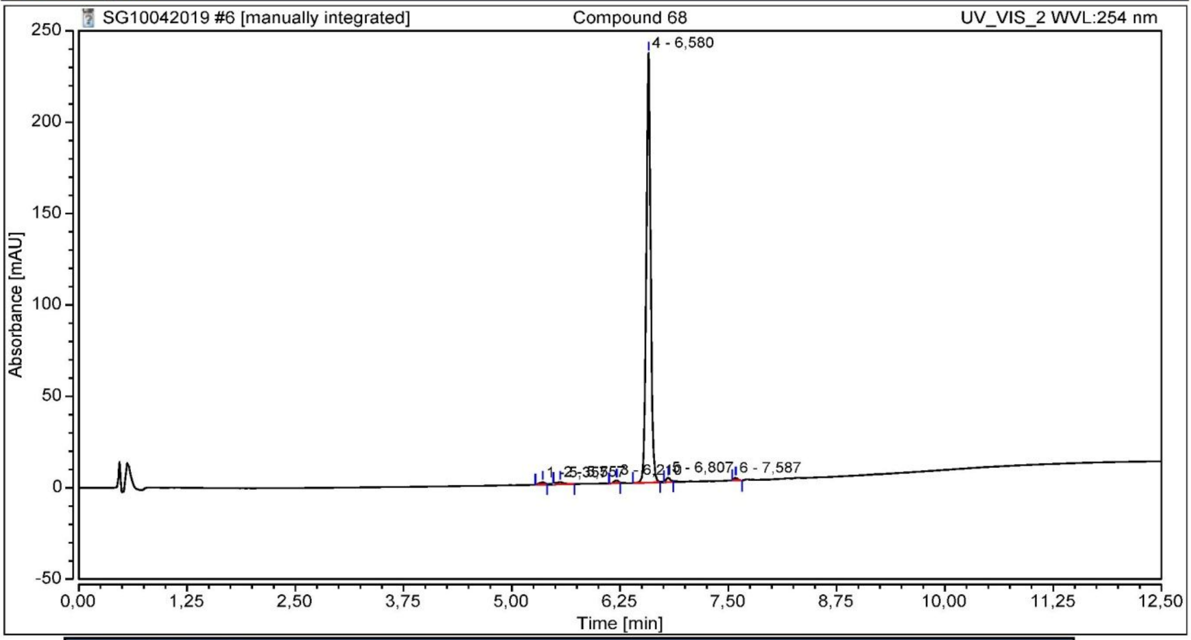

Integration Results

No.	Retention Time min	Area mAU*min	Height mAU	Relative Area %	Relative Height %
1	5,357	0,095	1,185	0,66	0,49
2	5,557	0,134	1,259	0,94	0,52
3	6,210	0,084	1,604	0,59	0,66
4	6,580	13,722	235,154	96,39	96,75
5	6,807	0,143	2,482	1,01	1,02
6	7,587	0,058	1,362	0,41	0,56
**Total:**		**14,236**	**243,046**	**100,00**	**100,00**

Compound **74**

Chromatogram and Results

Injection Details

Injection Name:	**Compound 74**	*Run Time (min):*	**12,50**
Processing Method:	**Quantitative**	*Injection Volume:*	**5,00**
Injection Date/Time:	**10.apr..19 14:58**	*Wavelength:*	**254**

Chromatogram

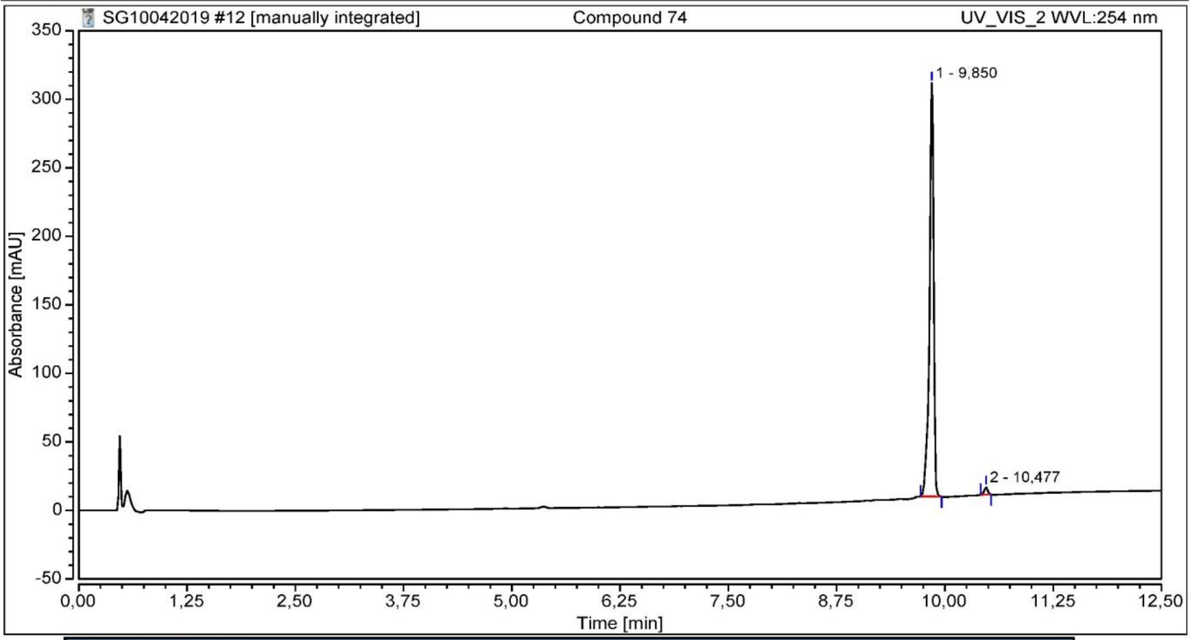

Integration Results

No.	Retention Time min	Area mAU*min	Height mAU	Relative Area %	Relative Height %
1	9,850	16,830	301,701	98,54	98,26
2	10,477	0,249	5,346	1,46	1,74
**Total:**		**17,079**	**307,047**	**100,00**	**100,00**

Compound **75**

Chromatogram and Results

Injection Details

Injection Name:	**Compound 75**	*Run Time (min):*	**12,50**
Processing Method:	**Quantitative**	*Injection Volume:*	**5,00**
Injection Date/Time:	**10.apr..19 16:27**	*Wavelength:*	**254**

Chromatogram

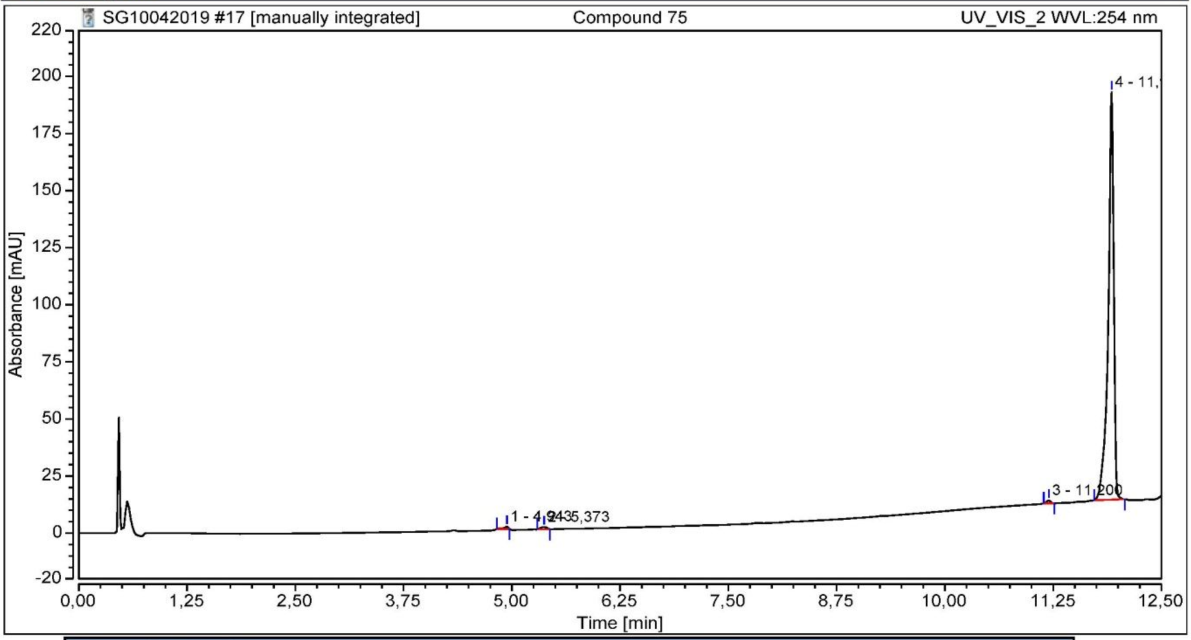

Integration Results

No.	Retention Time min	Area mAU*min	Height mAU	Relative Area %	Relative Height %
1	4,943	0,072	1,130	0,53	0,62
2	5,373	0,080	1,011	0,59	0,56
3	11,200	0,068	1,383	0,51	0,76
4	11,927	13,263	178,463	98,37	98,06
**Total:**		**13,483**	**181,987**	**100,00**	**100,00**

Compound **81**

Chromatogram and Results

Injection Details

Injection Name:	**Compound 81**	*Run Time (min):*	**12,50**
Processing Method:	**Quantitative**	*Injection Volume:*	**3,00**
Injection Date/Time:	**28.sep..20 17:07**	*Wavelength:*	**254**

Chromatogram

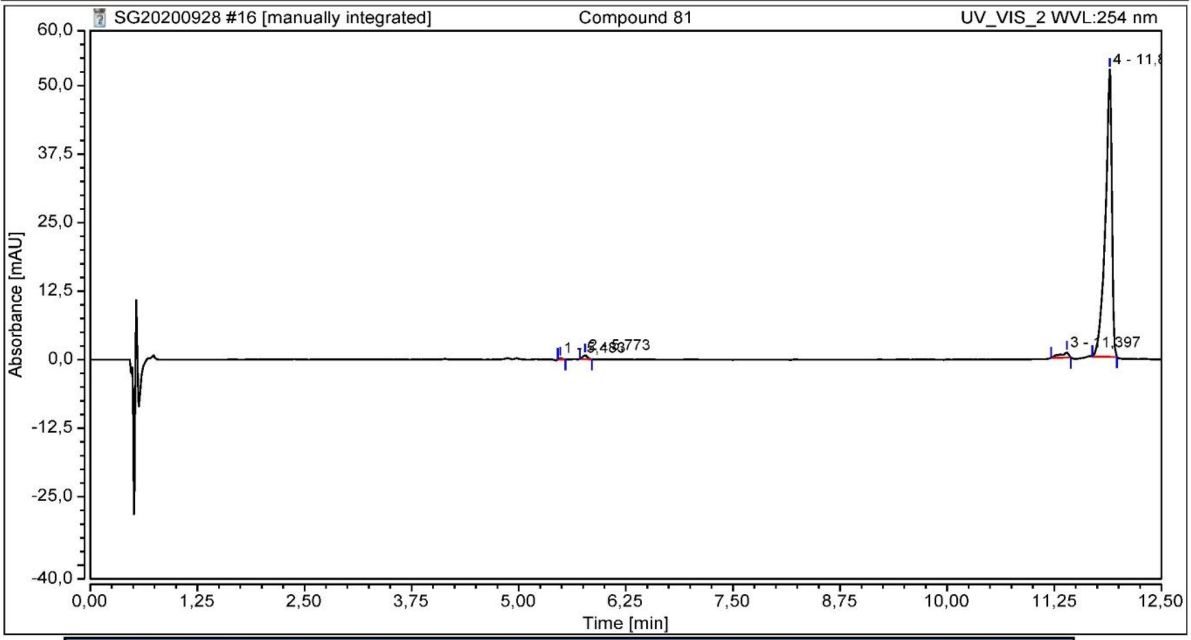

Integration Results

No.	Retention Time min	Area mAU*min	Height mAU	Relative Area %	Relative Height %
1	5,483	0,011	0,254	0,24	0,47
2	5,773	0,047	0,755	1,00	1,39
3	11,397	0,111	0,938	2,34	1,73
4	11,897	4,565	52,419	96,42	96,42
**Total:**		**4,734**	**54,366**	**100,00**	**100,00**

9 References

(1) Leschot, A.; Tapia, R. A.; Eyzaguirre, J. Efficient Synthesis of 4-Methylumbelliferyl Dihydroferulate. *Synth. Commun.* **2002**, *32*, 3219–3223.

(2) Min, T.; Yi, B.; Zhang, P.; Liu, J.; Zhang, C.; Zhou, H. Novel Furoxan NO-Donor Pemetrexed Derivatives: Design, Synthesis, and Preliminary Biological Evaluation. *Med. Chem. Res.* **2009**, *18*, 495–510.

(3) Salmela, M.; Sanmark, H.; Efimova, E.; Efimov, A.; Hytönen, V. P.; Lamminmäki, U.; Santala, S.; Santala, V. Molecular Tools for Selective Recovery and Detection of Lignin-Derived Molecules. *Green Chem.* **2018**, *20*, 2829–2839.

(4) Mo, R.; Sun, Q.; Xue, J.; Li, N.; Li, W.; Zhang, C.; Ping, Q. Multistage pH-Responsive Liposomes for Mitochondrial-Targeted Anticancer Drug Delivery. *Adv. Mater.* **2012**, *24*, 3659–3665.
